# Comparative evaluation of machine learning algorithms for Maize Lethal Necrosis Disease detection under class imbalance

**DOI:** 10.1371/journal.pone.0349738

**Published:** 2026-10-23

**Authors:** Maurice Wanyonyi

**Affiliations:** 1 Department of Mathematics and Statistics, University of Embu, Embu, Kenya; 2 African Institute for Capacity Development (AICAD), Nairobi, Kenya; Covenant University, NIGERIA

## Abstract

Maize Lethal Necrosis Disease (MLND) remains a major threat to maize production in sub-Saharan Africa, creating a need for accurate, interpretable, and computationally efficient diagnostic tools. This study presents a comprehensive comparative evaluation of nine machine learning algorithms for MLND detection using a structured feature-based dataset comprising 7,892 maize leaf observations obtained from the Kenya Agricultural and Livestock Research Organization (KALRO). The extracted features captured color, texture, lesion, and morphological characteristics associated with MLND symptoms. To address moderate class imbalance, the Synthetic Minority Over-sampling Technique (SMOTE) and class weighting were incorporated within the model development pipeline, and their contribution was quantified through an ablation study. Model performance was evaluated using repeated stratified 5-fold cross-validation, an independent 80/20 test set, and leave-one-county-out cross-validation (LOCO-CV) to assess geographic generalisability. Across repeated cross-validation, the leading models demonstrated consistent predictive performance with narrow 95% confidence intervals. CatBoost achieved the highest mean accuracy (0.753; 95% CI: 0.730–0.773), while Logistic Regression attained the highest mean area under the receiver operating characteristic curve (AUC = 0.808; 95% CI: 0.787–0.830). The overlapping confidence intervals among the best-performing models indicate broadly comparable predictive performance, highlighting the importance of considering complementary criteria beyond accuracy alone. LOCO-CV demonstrated minimal performance variation across counties, indicating good internal geographic generalisability. Calibration analysis using Expected Calibration Error (ECE) showed that post-hoc probability calibration substantially improved the reliability of predicted probabilities. At the same time, explainable artificial intelligence techniques consistently identified Chlorosis Intensity, Green–Yellow Ratio, and Necrotic Area as the most influential predictors. Considering predictive performance alongside computational efficiency, model size, and inference latency, LightGBM provided the most favorable balance for resource-constrained environments. In contrast, Logistic Regression offered a highly interpretable alternative with minimal computational requirements. Although the achieved performance indicates that approximately one in four samples may be misclassified and further external validation is required before operational deployment, the findings demonstrate that structured feature-based machine learning provides a transparent, computationally efficient, and interpretable framework for MLND detection and establishes a strong foundation for future field-based decision-support systems.

## Introduction

Maize is one of the most important staple crops globally and plays a critical role in food security, particularly in sub-Saharan Africa. However, its production is significantly threatened by plant diseases, among which Maize Lethal Necrosis Disease (MLND) remains one of the most devastating. The synergistic interaction of multiple viruses causes MLND and has led to substantial yield losses across East Africa, including Kenya, where maize is a primary food crop [1,2]. The rapid spread and severity of MLND necessitate timely and accurate detection methods to mitigate its impact and support sustainable agricultural practices.

Traditional methods for disease identification rely heavily on visual inspection by experts, which is often subjective, time-consuming, and not scalable in resource-constrained settings. Consequently, there has been growing interest in applying machine learning and deep learning techniques for automated plant disease detection [3,4]. Recent advances have demonstrated the potential of convolutional neural networks and related architectures to accurately classify maize diseases from leaf images [5–7]. Several studies have further explored transfer learning and advanced feature extraction techniques to improve classification accuracy [8–10]. These approaches have shown promising results in controlled experimental environments and benchmark datasets.

Despite these advancements, several critical challenges remain insufficiently addressed in the existing literature. First, many studies focus primarily on deep learning models without providing a comprehensive comparison with classical machine learning algorithms, which may offer competitive performance with lower computational cost [11–13]. Second, most existing works assume balanced datasets, whereas real-world agricultural data are often inherently imbalanced, with healthy samples significantly outnumbering diseased cases [3]. This imbalance can bias predictive models toward the majority class, resulting in poor detection of diseased plants, which is the class of greatest interest. Third, while some studies have addressed noise and annotation errors in datasets [14,15], the combined effect of data imbalance on model performance remains underexplored, particularly in the context of MLND detection.

In addition, existing MLND studies have largely focused on either remote sensing approaches [1,2] or deep learning-based image classification [16–18], with limited attention to structured feature-based datasets that capture interpretable visual characteristics such as chlorosis intensity, necrotic area, and lesion distribution. Such feature-based representations are particularly important for developing lightweight and interpretable models suitable for deployment in low-resource agricultural settings. Furthermore, available datasets are often limited in size or scope [19,20], which restricts the generalizability and robustness of developed models.

To address these gaps, this study proposes a comprehensive comparative evaluation of machine learning algorithms for MLND detection under class imbalance conditions using a structured feature-based dataset. Unlike prior studies that rely predominantly on image-based deep learning models, this work leverages interpretable visual and texture features to enable both accurate and explainable classification. The study systematically evaluates multiple machine learning algorithms and examines their robustness in the presence of imbalanced data, thereby providing insights into model suitability for real-world agricultural applications.

To address these gaps, this study presents a comprehensive evaluation of machine learning algorithms for MLND detection using a structured feature-based dataset derived from field observations obtained from the Kenya Agricultural and Livestock Research Organization (KALRO). Unlike previous studies that predominantly employ end-to-end deep learning models trained on raw RGB images, this study utilizes engineered color, texture, lesion, and morphological descriptors to develop interpretable machine learning models suitable for resource-constrained agricultural environments. In addition to comparative model evaluation, the proposed framework incorporates class imbalance handling, probability calibration, explainable artificial intelligence (XAI), geographic validation, and computational scalability assessment to provide a comprehensive evaluation of model performance under realistic agricultural conditions.

The novelty of this study lies not in the individual machine learning algorithms or explainability techniques themselves, but in their integrated application within a unified framework for MLND detection using structured visual descriptors derived from real field observations. Specifically, this study:

- provides a comprehensive comparison of classical linear, kernel-based, tree-based, ensemble, and neural network classifiers for MLND detection using engineered visual and texture features rather than raw image pixels;
- systematically quantifies the impact of class imbalance mitigation through ablation experiments and evaluates model calibration, extending model assessment beyond conventional accuracy-based comparisons;
- integrates complementary explainability techniques, including SHAP, LIME, and Partial Dependence Plots (PDPs), to verify that biologically meaningful disease characteristics drive model predictions;
- evaluates geographic generalisability using Leave-One-County-Out Cross-Validation (LOCO-CV), providing evidence of model consistency across different maize-growing regions in Kenya; and
- assesses computational efficiency, memory requirements, and inference latency to identify models suitable for deployment in resource-constrained agricultural settings.

The principal contributions of this study are therefore fourfold. First, it demonstrates that carefully engineered visual and texture features extracted from real field observations provide sufficient discriminatory information for effective MLND detection using conventional machine learning algorithms. Second, it establishes a comprehensive evaluation framework that jointly considers predictive performance, class imbalance handling, calibration, explainability, scalability, and geographic generalisability, rather than relying solely on classification accuracy. Third, it identifies the trade-offs between simple interpretable models and more complex ensemble methods, providing practical guidance for selecting appropriate models under different deployment constraints. Finally, the study provides reproducible evidence supporting the use of interpretable machine learning as a computationally efficient alternative to end-to-end deep learning approaches for agricultural disease detection.

Overall, this work advances current MLND research by presenting a comprehensive and practically oriented evaluation framework that integrates predictive performance, interpretability, calibration, scalability, and geographic validation using real field-derived data. The findings provide practical guidance for developing transparent, computationally efficient, and reliable decision-support systems for precision agriculture and resource-constrained farming environments.

## Materials and methods

### Study design and data source

This study adopted a quantitative, comparative machine learning framework to evaluate the performance of multiple supervised learning algorithms for detecting MLND under class-imbalance conditions. The analysis was conducted using a secondary dataset obtained from the Kenya Agricultural and Livestock Research Organization (KALRO). The original images were acquired from farmers’ fields across multiple maize-growing counties in Kenya under varying field conditions. KALRO plant pathologists visually confirmed MLND infection status based on established symptom criteria, and the structured features were subsequently extracted from the images using KALRO’s proprietary image analysis pipeline. The resulting dataset was provided to the authors in an anonymized, tabular format containing only the engineered features and associated labels, with no raw images or identifiable farmer information included. This secondary dataset formed the basis for all analyses in the present study.

The dataset comprised 7,892 observations collected from maize-growing counties in Kenya and consisted of structured numerical features derived from maize leaf images. These features describe the visual characteristics of maize leaves relevant to MLND diagnosis, including color, texture, lesion morphology, and spatial distribution. As the study utilized a secondary dataset, the original image acquisition and feature generation procedures were undertaken by KALRO before data acquisition. Consequently, the present study focuses on the comparative evaluation of machine learning algorithms using the provided structured features.

### Variables and feature description

The response variable was *Disease_Label*, a binary outcome defined as


$$\begin{array}{c} Y\in \{0,1\}, \end{array}$$

where *Y* = 0 denotes a healthy maize leaf and *Y* = 1 denotes an MLND-infected maize leaf.

The predictor variables consisted of one categorical variable (*County*) and eleven continuous variables normalized to the interval [0,1]. These variables quantify different visual characteristics associated with MLND symptoms and can be grouped into four categories:

- **Color features:** Chlorosis Intensity, Green–Yellow Ratio, Greenness Intensity, and Color Uniformity, which describe changes in leaf pigmentation caused by disease progression.
- **Lesion features:** Necrotic Area, Lesion Distribution, and Infected Distribution, which quantify the extent and spatial distribution of diseased tissue.
- **Texture features:** Texture Roughness and Transition Smoothness, which characterize variations in leaf surface appearance and symptom progression.
- **Morphological and edge features:** Boundary Sharpness and Leaf Symmetry, which capture changes in lesion boundaries and overall leaf morphology associated with MLND infection.

Scaling all continuous variables to the interval [0,1] ensured numerical comparability across features and facilitated consistent model training and optimization.

### Data preprocessing

Before model development, the dataset underwent several preprocessing steps. The categorical variable County was one-hot encoded to transform it into a numerical representation suitable for machine learning algorithms. Continuous variables were standardized using z-score normalization defined as:


$$\begin{array}{c} x^{*}=\frac{x-\mu }{\sigma } \end{array}$$ (1)

where μ and σ denote the mean and standard deviation of each feature, respectively; standardization is particularly important for distance-based models such as support vector machines and k-nearest neighbors.

### Handling class imbalance

The dataset exhibits class imbalance, with a higher proportion of healthy samples than MLND-infected samples. To mitigate potential bias toward the majority class, both data-level and algorithm-level imbalance-handling strategies were employed.

### Data-level strategy: SMOTE

At the data level, the Synthetic Minority Over-sampling Technique (SMOTE) was applied using the implementation provided by the imbalanced-learn library [21]. The standard SMOTE algorithm generates synthetic minority-class samples by interpolating between a minority-class instance and one of its *k*-nearest neighbors [3,22]. The oversampling ratio was set to 1:1, such that the minority class (MLND) was oversampled to match the number of majority-class (Healthy) samples. The number of nearest neighbors used to generate synthetic samples was set to the default value of *k* = 5. No Edited Nearest Neighbors (ENN) undersampling or SMOTE-ENN hybrid resampling was performed in this study.

Importantly, SMOTE was applied only after the train–test split and exclusively within each cross-validation training fold to prevent data leakage. Specifically, SMOTE was incorporated into an imblearn.pipeline.Pipeline, ensuring that oversampling was performed solely on the training data during both the initial 80:20 train–test split and all cross-validation procedures. Consequently, the test and validation sets remained completely untouched by the resampling process, providing unbiased estimates of model performance on unseen data.

### Algorithm-level strategy: Class weighting

In addition to data-level resampling, algorithm-level class weighting was employed for models that support this mechanism. For classifiers such as Logistic Regression, Support Vector Machines, and tree-based ensemble methods, class weights were set inversely proportional to class frequencies, thereby penalizing misclassification of the minority class more heavily. The weighted loss function is given by


$$\begin{array}{c} L=-\sum_{i=1}^{n}w_{y_{i}}\left[y_{i}\log(p_{i})+(1-y_{i})\log(1-p_{i})\right], \end{array}$$ (2)

where $w_{y_{i}}$ denotes the class-specific weight, calculated as


$$\begin{array}{c} w_{y_{i}}=\frac{n_{samples}}{n_{classes}\times n_{y_{i}}}, \end{array}$$ (3)

with *n*_samples_ representing the total number of samples, *n*_classes_ = 2 the number of classes, and $n_{y_{i}}$ the number of observations belonging to class $y_{i}$. For models that did not natively support class weighting (e.g., XGBoost), the scale_pos_weight parameter was set to the ratio of negative to positive samples to achieve a similar weighting effect.

### Validation strategy

To minimize the risk of resampling-associated overfitting, all hyperparameter optimization and model selection were performed using stratified cross-validation after resampling. For each cross-validation fold:

1. The training subset was resampled using SMOTE.
2. The Model was trained on the resampled training data.
3. Performance was evaluated on the corresponding held-out validation fold, which was not resampled.

This procedure ensured that model evaluation was always performed on unmodified data, thereby providing realistic estimates of predictive performance and improving the generalisability of the reported results.

### Machine learning model implementation

Before model training, a structured feature engineering pipeline was applied to transform the original maize leaf images into quantitative descriptors suitable for machine learning. The extracted features captured complementary characteristics of MLND symptoms, including color, texture, lesion morphology, and spatial distribution. This feature engineering stage reduced the dimensionality of the original image data while preserving biologically meaningful information relevant to disease discrimination.

To provide a comprehensive comparison, nine supervised machine learning algorithms were evaluated. These algorithms were selected because they represent different learning paradigms commonly applied in agricultural disease detection, including linear models (Logistic Regression), kernel-based learning (Support Vector Machine), tree-based learning (Decision Tree), distance-based learning (k-Nearest Neighbors), ensemble bagging (Random Forest), gradient boosting (XGBoost, LightGBM, and CatBoost), and neural networks (Multi-Layer Perceptron). Evaluating diverse algorithms using the same engineered feature set enabled assessment of whether increased model complexity translated into improved predictive performance while keeping the input representation constant.

All models were optimized using grid search with cross-validation to identify the best-performing hyperparameter combinations.

### Logistic regression

Logistic Regression was included as a baseline linear classifier because of its simplicity, interpretability, and widespread use in binary classification problems. It estimates the probability of class membership using the sigmoid function.


$$\begin{array}{c} P(Y=1|X)=\frac{1}{1+e^{-(\beta_{0}+\beta^{T}X)}}. \end{array}$$ (4)

### Support vector machine

Support Vector Machines (SVM) were selected because of their ability to model complex non-linear decision boundaries through kernel functions while maintaining good generalization performance in high-dimensional feature spaces. The decision function is


$$\begin{array}{c} f(x)=\text{sign}\left(\sum_{i=1}^{n}\alpha_{i}y_{i}K(x_{i},x)+b\right), \end{array}$$ (5)

where K(·) denotes the kernel function.

### Decision tree

Decision Trees were included because they provide highly interpretable rule-based classification and serve as the foundation for many ensemble learning methods. The tree recursively partitions the feature space by maximizing information gain based on entropy,


$$\begin{array}{c} H(Y)=-\sum_{i}p_{i}\log p_{i}. \end{array}$$ (6)

### k-nearest neighbours

The k-Nearest Neighbors (KNN) algorithm was evaluated as a representative instance-based learning method that classifies a sample according to the majority class among its nearest neighbors using Euclidean distance,


$$\begin{array}{c} d(x_{i},x_{j})=\sqrt{\sum_{k=1}^{p}(x_{ik}-x_{jk}{)}^{2}}. \end{array}$$ (7)

### Random forest

Random Forest was selected because bootstrap aggregation reduces the variance associated with individual decision trees and generally improves robustness to noisy agricultural datasets. Predictions are obtained by averaging the outputs of multiple trees,


$$\begin{array}{c} \hat{f}(x)=\frac{1}{B}\sum_{b=1}^{B}f_{b}(x), \end{array}$$ (8)

where *B* is the number of trees.

### XGBoost

XGBoost was included as a widely adopted gradient boosting algorithm capable of modeling complex feature interactions while incorporating regularisation to reduce overfitting. The objective function is


$$\begin{array}{c} ℒ=\sum_{i=1}^{n}l(y_{i},{\hat{y}}_{i})+\sum_{k=1}^{K}\Omega (f_{k}), \end{array}$$ (9)

where l(·) is the loss function and Ω(·) represents the regularisation term.

### LightGBM

LightGBM was evaluated because it is designed for efficient gradient boosting on structured datasets through histogram-based learning and leaf-wise tree growth, which reduce training time while maintaining predictive accuracy. At each iteration, the Model updates predictions by adding a new decision tree,


$$\begin{array}{c} F_{m}(x)=F_{m-1}(x)+\eta h_{m}(x), \end{array}$$ (10)

where $F_{m}(x)$ is the updated prediction, $h_{m}(x)$ is the newly fitted tree, and η is the learning rate. The leaf-wise growth strategy enables LightGBM to achieve high predictive performance with relatively low computational cost, making it well suited for resource-constrained applications.

### CatBoost

CatBoost was included because it addresses prediction shift during boosting through ordered boosting and uses symmetric (oblivious) decision trees that improve generalization and computational efficiency. Similar to other gradient boosting methods, CatBoost updates the Model iteratively according to


$$\begin{array}{c} F_{m}(x)=F_{m-1}(x)+\eta h_{m}(x), \end{array}$$ (11)

where $h_{m}(x)$ represents the tree fitted to the residual errors at iteration *m*. The ordered boosting strategy reduces target leakage during training, while symmetric tree construction improves inference speed and model stability.

### Multi-layer perceptron

A Multi-Layer Perceptron (MLP) was included as a representative feed-forward neural network capable of learning complex non-linear relationships between engineered features. The hidden layer transformation is


$$\begin{array}{c} h=\sigma (Wx+b), \end{array}$$ (12)

where σ(·) denotes the activation function, *W* the weight matrix, and *b* the bias vector.

### Data splitting and model training

The dataset was initially partitioned into training and testing sets using an 80:20 stratified split to preserve the original class distribution in both subsets. All preprocessing steps, including feature scaling, class imbalance handling (SMOTE and class weighting), and hyperparameter optimization, were performed using the training data only to prevent information leakage. Hyperparameters for each classifier were optimized through grid search with stratified 5-fold cross-validation on the training set. The final models were subsequently evaluated on the independent test set, which remained unseen throughout model development.

To further assess model stability and generalisability beyond a single train-test split, repeated stratified 5-fold cross-validation (10 repetitions) was conducted on the complete dataset, and performance was summarised using the mean and standard deviation of the evaluation metrics. In addition, leave-one-county-out cross-validation (LOCO-CV) was performed to evaluate the ability of the models to generalize to unseen geographic regions by sequentially holding out data from one County for testing while training on the remaining counties.

### Hyperparameter optimisation and reproducibility

All machine learning models were trained within a unified pipeline that included standard scaling for numerical features, one‑hot encoding for the categorical variable (County), and Synthetic Minority Over‑sampling Technique (SMOTE) to address class imbalance. To avoid data leakage, SMOTE was applied inside cross‑validation folds using an imbalanced-learn pipeline.

Hyperparameter tuning was performed using GridSearchCV with 3‑fold stratified cross‑validation on the training set (80% of the data). The optimization metric was the Area Under the Receiver Operating Characteristic curve (ROC–AUC). The search space for each Model is detailed in Table 1.

**Table 1:** Hyperparameter search ranges for each classifier used in this study.

Model	Hyperparameter	Values Searched
Logistic Regression	C (reg. strength) penalty solver	0.01, 0.1, 1, 10, 100 l1, l2 liblinear, saga
SVM	C gamma kernel	0.1, 1, 10, 100 scale, auto, 0.01, 0.1 rbf, poly
Decision Tree	max_depth min_samples_split min_samples_leaf	3, 5, 10, None 2, 5, 10 1, 2, 4
K-Nearest Neighbours	n_neighbors weights p (distance)	3, 5, 7, 11, 15 uniform, distance 1, 2
Random Forest	n_estimators max_depth min_samples_split	50, 100, 200 None, 10, 20 2, 5, 10
XGBoost	learning_rate max_depth n_estimators subsample colsample_bytree	0.01, 0.1, 0.3 3, 5, 7 50, 100, 200 0.8, 1.0 0.8, 1.0
LightGBM	learning_rate max_depth n_estimators num_leaves	0.01, 0.1, 0.3 **−1, 5, 6, 10** 50, 100, 200 15, 31, 63
CatBoost	learning_rate depth iterations	0.01, 0.1, 0.3 3, 5, 7 50, 100, 200
Multi-layer Perceptron	hidden_layer_sizes activation alpha (L2 penalty)	(50,), (100,), (50,50) relu, tanh 0.0001, 0.001, 0.01

To guarantee reproducibility, a fixed random seed of 42 was set for all stochastic operations, including:

- train–test splitting,
- cross‑validation fold generation,
- SMOTE oversampling,
- model initialisation (e.g., random_state in Random Forest, XGBoost, etc.).

Stopping criteria were configured as follows:

- **Logistic Regression**: max_iter = 1000.
- **MLP**: max_iter = 500.
- **XGBoost, LightGBM and CatBoost**: the number of boosting rounds (n_estimators/iterations) was directly optimized via the grid search; no early‑stopping was used during tuning to maintain a consistent evaluation framework across all models.

### Model evaluation metrics

Given the class imbalance inherent in the dataset, a single metric such as accuracy is insufficient for reliable model assessment. Therefore, a suite of complementary metrics was employed.

Accuracy, defined as the proportion of correctly classified instances, serves as a baseline measure:


$$\begin{array}{c} \text{Accuracy}=\frac{TP+TN}{TP+TN+FP+FN}. \end{array}$$ (13)

However, because accuracy can be misleading under imbalance, precision and recall were also computed. Precision quantifies the proportion of positive predictions that are truly positive, while recall (sensitivity) measures the proportion of actual positives correctly identified:


$$\begin{array}{c} \text{Precision}=\frac{TP}{TP+FP} \end{array}$$ (14)

$$\begin{array}{c} \text{Recall}=\frac{TP}{TP+FN}. \end{array}$$ (15)

These two metrics were combined into the harmonic mean known as the F1 score, which provides a balanced assessment:


$$\begin{array}{c} F1=2\cdot \frac{\text{Precision}\cdot \text{Recall}}{\text{Precision}+\text{Recall}}. \end{array}$$ (16)

Finally, the Area Under the Receiver Operating Characteristic Curve (AUC) was used to evaluate the Model’s discrimination capability under class imbalance. The AUC summarizes the trade‑off between the true positive rate and the false positive rate across all classification thresholds, with higher values indicating better separation between healthy and infected leaves.

### Internal validation and generalisability assessment

To evaluate the robustness and generalisability of the developed models beyond the simple 80/20 train–test split, we employed a comprehensive internal validation framework. First, we performed a repeated stratified 5‑fold cross‑validation (10 repeats) on the entire dataset to obtain a distribution of performance metrics (AUC, accuracy, precision, recall, F1) and their 95% confidence intervals. This approach reduces the variance associated with a single random split and provides a more stable estimate of model performance.

Second, to specifically assess the Model’s ability to generalize to unseen geographical regions, we conducted a leave‑one‑county‑out cross‑validation (LOCO‑CV) using the County variable as the grouping factor. In each fold, all data from one County were held out as the test set, while the remaining counties were used for training and hyperparameter tuning (with internal 3‑fold CV within the training folds). This simulates a realistic scenario where the Model is deployed in a new location and tests whether the learned patterns are consistent across different administrative regions.

### Scalability analysis

Beyond predictive performance, the practical utility of a machine learning model for field‑based disease detection depends on its computational efficiency and resource footprint. To assess scalability, four complementary metrics were evaluated for each algorithm.

**Training time** measures the total clock time required to fit the Model on the training set (80% of the data). This includes data preprocessing, hyperparameter optimization, and the final Model fitting. Shorter training times facilitate rapid model iteration and re‑training as new field data become available.

**Inference latency** is the time taken to classify a single unseen sample (i.e., one maize leaf record). Low latency is critical for real-time or near-real-time applications, such as mobile-based diagnostic tools used directly in the field. The average latency over 1,000 predictions was recorded for each algorithm.

**Model size** refers to the storage space required to serialize the trained Model (e.g., file size in megabytes). Compact models are easier to distribute, update, and deploy on edge devices with limited storage capacity.

### Explainable artificial intelligence

To enhance interpretability, three explainable AI techniques were employed

### SHAP (SHapley Additive exPlanations)

SHAP values quantify the contribution of each feature to the prediction based on cooperative game theory:


$$\begin{array}{c} \phi_{i}=\sum_{S\subseteq N⧵\{i\}}\frac{|S|!(|N|-|S|-1)!}{|N|!}[f(S\cup \{i\})-f(S)] \end{array}$$ (17)

### Partial dependence plots

Partial dependence plots illustrate the marginal effect of a feature on the predicted outcome:


$$\begin{array}{c} PD(x_{s})=\frac{1}{n}\sum_{i=1}^{n}f(x_{s},x_{C}) \end{array}$$ (18)

### LIME

Local Interpretable Model-agnostic Explanations approximates complex models locally using interpretable linear models.

### Implementation and computational environment

All analyses were implemented in Python (version 3.13.5) using a suite of open‑source machine learning libraries. Experiments were executed on a workstation equipped with an Intel(R) Core(TM) i5‑7Y54 CPU @ 1.20 GHz (1.61 GHz) processor and 8.00 GB (7.88 GB usable) of RAM, running the Windows 11 Pro operating system (Version 23H2, OS build 22631.6199). To ensure full reproducibility, a fixed random seed of 42 was set for all stochastic operations, including train–test splits, cross‑validation fold generation, SMOTE oversampling, and model initialization. The key Python packages and their exact versions were recorded as follows: scikit-learn (1.3.0), xgboost (2.0.0), lightgbm (4.1.0), catboost (1.2.0), imbalanced-learn (0.11.0), shap (0.42.0), lime (0.2.0.1), numpy (1.24.0), and pandas (2.0.0).

### Ethical statement

This study was conducted in accordance with ethical principles for research using secondary agricultural data. The dataset was obtained from the Kenya Agricultural and Livestock Research Organization (KALRO) under a formal data use agreement that permits analysis for scientific and educational purposes. All data were fully anonymized before receipt; no personally identifiable information, farmer identifiers, or precise geolocations were included. As the study involved no human subjects, live animals, or experimental interventions, institutional review board (IRB) approval was not required. The author declares no conflicts of interest, and the research adhered to the ethical guidelines of the collaborating institutions.

## Results

### Exploratory data analysis

Before model development, an exploratory data analysis was conducted to examine the structure, Distribution, and relationships among the variables in the dataset. The dataset comprised 7,892 observations collected from major maize-growing counties in Kenya, with each observation representing a maize leaf sample characterized by eleven continuous features and one categorical feature (County).

Fig 1 illustrates the Distribution of the target variable, Disease Label, where 0 denotes a healthy leaf, and 1 denotes a leaf infected with MLND. The healthy class consisted of 5,400 samples, while the infected class included 2,492 samples, indicating a moderate class imbalance with a healthy-to-infected ratio of approximately 2.17:1. This imbalance reflects real-world agricultural conditions, where healthy plants typically outnumber diseased ones.

**Fig 1:**
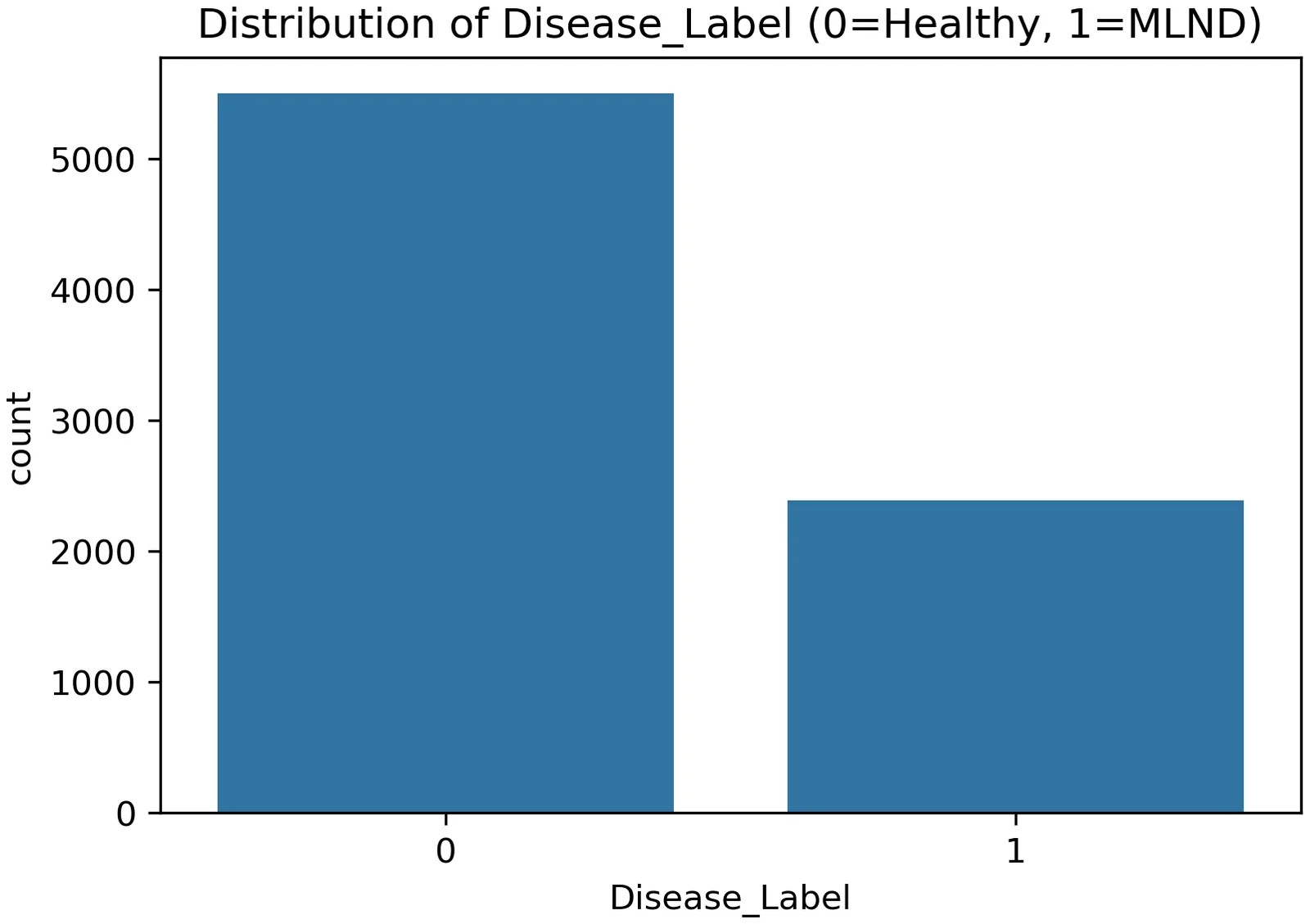
Class distribution of the target variable. Distribution of Disease Label in the dataset. Class 0 represents healthy maize leaves (5,400 samples), and class 1 represents MLND-infected leaves (2,492 samples). The imbalance ratio is approximately 2.17:1.

Fig 2 presents boxplots comparing the Distribution of selected features (Chlorosis Intensity, Green Yellow Ratio, Necrotic Area, and Texture Roughness) across healthy and MLND-infected classes. Infected leaves exhibit higher median values and greater variability for Chlorosis Intensity and Necrotic Area, indicating increased yellowing and tissue necrosis associated with disease progression. In contrast, the Green Yellow Ratio is lower among infected leaves, reflecting reduced healthy green tissue relative to chlorotic regions. Texture Roughness is also elevated in infected leaves, consistent with the presence of lesions and structural irregularities. These patterns demonstrate that the selected features effectively capture key symptomatic differences between healthy and diseased maize leaves.

**Fig 2:**
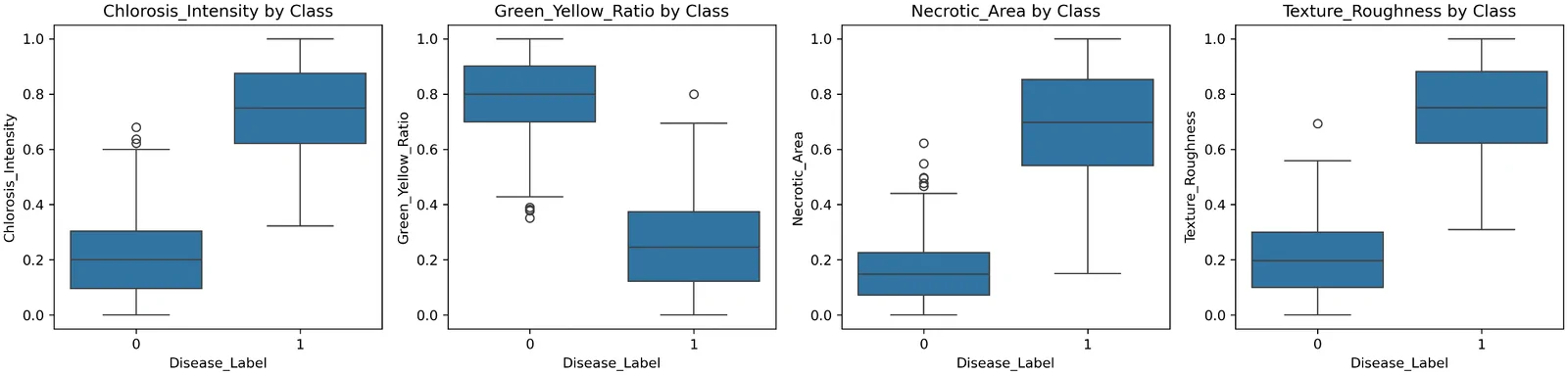
Boxplots of selected features by class. Comparison of representative features between healthy (label 0) and MLND-infected (label 1) leaves. Infected leaves show higher Chlorosis Intensity and Necrotic Area, lower Green Yellow Ratio, and higher Texture Roughness.

Overall, the exploratory analysis indicates that the dataset reflects realistic class imbalance and that the engineered visual and texture features exhibit meaningful differences between healthy and infected classes. These findings support the use of imbalance handling techniques such as SMOTE and provide a strong foundation for subsequent comparative machine learning analyses.

### Model performance evaluation

The predictive performance of the evaluated machine learning models is summarized in Table 2. Performance is reported together with 95% confidence intervals to quantify the uncertainty associated with each evaluation metric.

**Table 2:** Performance comparison of machine learning models for MLND classification. Values are reported as point estimates with 95% confidence intervals.

Model	Acc.	Prec.	Rec.	F1	AUC	Brier
LR	0.745 (0.724–0.766)	0.626 (0.591–0.660)	0.730 (0.694–0.763)	0.674 (0.644–0.701)	0.808 (0.787–0.830)	0.176 (0.166–0.186)
SVM	0.726 (0.704–0.747)	0.611 (0.576–0.649)	0.665 (0.624–0.703)	0.637 (0.606–0.666)	0.789 (0.765–0.810)	0.182 (0.171–0.193)
DT	0.648 (0.624–0.671)	0.511 (0.473–0.549)	0.582 (0.540–0.622)	0.544 (0.510–0.576)	0.634 (0.608–0.657)	0.352 (0.329–0.376)
KNN	0.672 (0.649–0.694)	0.537 (0.500–0.573)	0.665 (0.626–0.703)	0.594 (0.561–0.624)	0.707 (0.681–0.733)	0.236 (0.223–0.250)
RF	0.750 (0.730–0.770)	0.662 (0.625–0.699)	0.632 (0.591–0.670)	0.646 (0.615–0.676)	0.799 (0.777–0.821)	0.175 (0.167–0.183)
XGB	0.703 (0.680–0.725)	0.571 (0.536–0.606)	0.712 (0.674–0.746)	0.634 (0.604–0.664)	0.773 (0.746–0.796)	0.212 (0.198–0.226)
LGBM	0.750 (0.728–0.771)	0.654 (0.616–0.694)	0.656 (0.613–0.693)	0.655 (0.622–0.685)	0.803 (0.779–0.825)	0.171 (0.160–0.183)
CatB	0.753 (0.730–0.773)	0.664 (0.625–0.703)	0.639 (0.597–0.679)	0.651 (0.617–0.682)	0.804 (0.780–0.825)	0.170 (0.160–0.182)
MLP	0.691 (0.669–0.712)	0.571 (0.533–0.609)	0.575 (0.533–0.616)	0.573 (0.539–0.605)	0.728 (0.702–0.752)	0.229 (0.215–0.245)

**Note:** LR, Logistic Regression; SVM, Support Vector Machine; DT, Decision Tree; KNN, k-Nearest Neighbours; RF, Random Forest; XGB, Extreme Gradient Boosting (XGBoost); LGBM, Light Gradient Boosting Machine (LightGBM); CatB, CatBoost; MLP, Multilayer Perceptron. Acc., accuracy; Prec., precision; Rec., recall; F1, F1 score; AUC, area under the Receiver Operating Characteristic curve; Brier, Brier score.

Overall, CatBoost achieved the highest point estimate for accuracy (0.753; 95% CI: 0.730–0.773), closely followed by LightGBM (0.750; 95% CI: 0.728–0.771) and Random Forest (0.750; 95% CI: 0.730–0.770). The substantial overlap in the confidence intervals suggests that these ensemble models exhibited comparable predictive performance, and the observed differences in point estimates should therefore be interpreted with caution. Logistic Regression also performed competitively, achieving an accuracy of 0.745 (95% CI: 0.724–0.766) and the highest recall (0.730; 95% CI: 0.694–0.763), highlighting its strong ability to identify MLND-infected samples.

CatBoost (0.664; 95% CI: 0.625–0.703) and Random Forest (0.662; 95% CI: 0.625–0.699) achieved the highest precision, indicating fewer false-positive predictions. Logistic Regression produced the highest F1 score (0.674; 95% CI: 0.644–0.701), reflecting the best balance between precision and recall among the evaluated models. Logistic Regression also achieved the highest AUC (0.808; 95% CI: 0.787–0.830), closely followed by CatBoost (0.804; 95% CI: 0.780–0.825) and LightGBM (0.803; 95% CI: 0.779–0.825), demonstrating strong discriminative ability across these models.

In contrast, Decision Tree consistently exhibited the weakest performance across all evaluation metrics, achieving the lowest accuracy (0.648; 95% CI: 0.624–0.671), F1 score (0.544; 95% CI: 0.510–0.576), and AUC (0.634; 95% CI: 0.608–0.657). KNN and MLP also showed comparatively lower performance, suggesting limited ability to capture the complex relationships present in the feature space.

Brier scores further indicated differences in probabilistic prediction quality. CatBoost (0.170; 95% CI: 0.160–0.182), LightGBM (0.171; 95% CI: 0.160–0.183), Random Forest (0.175; 95% CI: 0.167–0.183), and Logistic Regression (0.176; 95% CI: 0.166–0.186) produced the lowest Brier scores, indicating good overall probabilistic accuracy. Decision Tree exhibited the highest Brier score (0.352; 95% CI: 0.329–0.376), reflecting comparatively poor probability estimation.

### Internal validation and generalisability assessment

To assess the robustness and generalisability of the developed classifiers beyond a single train–test split, we performed repeated stratified 5-fold cross-validation (10 repeats) and leave-one-county-out cross-validation (LOCO-CV). Repeated cross-validation was used to obtain stable estimates of predictive performance and corresponding 95% confidence intervals. At the same time, LOCO-CV evaluated the models’ ability to generalize to geographically unseen regions by holding out one County for testing and training on the remaining counties.

### Leave-one-county-out cross-validation results

Table 3 summarises the LOCO-CV performance of the nine machine learning classifiers. Values are reported as the mean and standard deviation across all counties.

**Table 3:** Leave-one-county-out cross-validation (LOCO-CV) performance (mean ± SD) across all counties.

Model	AUC	Acc.	Prec.	Rec.	F1
Logistic Regression	0.8163 ± 0.0064	0.7413 ± 0.0062	0.6165 ± 0.0233	0.7460 ± 0.0116	0.6748 ± 0.0144
SVM	0.8047 ± 0.0087	0.7394 ± 0.0095	0.6182 ± 0.0280	0.7240 ± 0.0086	0.6666 ± 0.0174
Decision Tree	0.6364 ± 0.0102	0.6536 ± 0.0053	0.5173 ± 0.0275	0.5744 ± 0.0263	0.5439 ± 0.0211
KNN	0.7185 ± 0.0097	0.6724 ± 0.0076	0.5362 ± 0.0272	0.6752 ± 0.0165	0.5973 ± 0.0187
Random Forest	0.8079 ± 0.0070	0.7600 ± 0.0101	0.6698 ± 0.0281	0.6578 ± 0.0193	0.6635 ± 0.0201
XGBoost	0.7921 ± 0.0046	0.6963 ± 0.0052	0.5576 ± 0.0221	0.7559 ± 0.0143	0.6417 ± 0.0183
LightGBM	0.8134 ± 0.0054	0.7534 ± 0.0078	0.6525 ± 0.0205	0.6757 ± 0.0147	0.6636 ± 0.0111
CatBoost	0.8188 ± 0.0065	0.7600 ± 0.0083	0.6602 ± 0.0209	0.6882 ± 0.0193	0.6736 ± 0.0140
MLP	0.7697 ± 0.0139	0.7119 ± 0.0167	0.5968 ± 0.0497	0.6536 ± 0.0716	0.6190 ± 0.0215

The LOCO-CV results demonstrate that the highest-performing classifiers retained strong predictive performance when evaluated on counties that were completely excluded from model training. CatBoost achieved the highest mean AUC (0.8188 ± 0.0065), followed closely by Logistic Regression (0.8163 ± 0.0064), LightGBM (0.8134 ± 0.0054), Random Forest (0.8079 ± 0.0070), and SVM (0.8047 ± 0.0087). These models also exhibited low standard deviations across counties (AUC SD < 0.01), indicating consistent predictive performance despite geographic variation.

Random Forest and CatBoost achieved the highest mean classification accuracy (0.7600), while Logistic Regression produced the highest recall (0.7460) among the top-performing models. XGBoost attained the greatest recall overall (0.7559), suggesting a greater ability to identify infected plants, although this was accompanied by lower precision (0.5576). In contrast, the Decision Tree consistently produced the weakest performance across all evaluation metrics, with the lowest AUC (0.6364) and F1-score (0.5439), indicating limited generalisability.

Overall, the LOCO-CV performance closely matched the results obtained using the conventional 80/20 train–test split (Table 2). Differences in AUC for the leading models were generally less than 0.02, indicating minimal performance degradation when predicting samples from previously unseen counties. The slightly higher AUC values observed during LOCO-CV likely reflect the larger effective training set available in each fold.

These findings indicate that the proposed models learned disease characteristics that generalize across geographic regions rather than county-specific patterns. The consistently strong performance of CatBoost, Logistic Regression, LightGBM, and Random Forest suggests that the extracted color and texture features capture stable visual manifestations of maize lethal necrosis disease that are largely invariant across the sampled counties.

### Statistical comparison of model performance

To determine whether the observed differences in predictive performance were statistically significant, multiple complementary statistical tests were performed. Pairwise Wilcoxon signed-rank tests were conducted on cross-validation AUC scores, bootstrap tests were used to compare AUC differences on the independent test set, McNemar’s test was applied to paired classification predictions, and a Friedman test followed by the Nemenyi post-hoc procedure was used to compare all classifiers simultaneously.

The Wilcoxon signed-rank tests in Table 4 indicated that Logistic Regression and CatBoost exhibited statistically indistinguishable AUC performance (*p* = 0.546), whereas most other pairwise comparisons were statistically significant (*p* < 0.05). However, bootstrap comparisons of AUC differences on the independent test set showed no statistically significant differences among the highest-performing models. In particular, CatBoost and LightGBM exhibited nearly identical AUC values (ΔAUC = −0.001, *p* = 0.822), while Logistic Regression did not differ significantly from either CatBoost (*p* = 0.558) or LightGBM (*p* = 0.539).

**Table 4:** Summary of statistical comparisons among the highest-performing machine learning models.

Statistical test	Key finding
Wilcoxon signed-rank test (AUC)	Logistic Regression and CatBoost showed no significant difference in AUC performance (*p* = 0.546).
Bootstrap comparison (AUC)	LightGBM and CatBoost exhibited nearly identical AUC values (ΔAUC=−0.001, *p* = 0.822).
McNemar’s test	Random Forest and LightGBM showed no significant difference in prediction errors ($\chi^{2}=0.008$, *p* = 0.931).
McNemar’s test	LightGBM and CatBoost also produced statistically indistinguishable prediction errors ($\chi^{2}=0.090$, *p* = 0.764).
Nemenyi post-hoc test	No significant difference was observed between Logistic Regression and CatBoost (adjusted *p* = 1.000) or between Random Forest and LightGBM (adjusted *p* = 1.000).

Similarly, McNemar’s test revealed no significant differences in classification errors between Random Forest and LightGBM (*p* = 0.931), Random Forest and CatBoost (*p* = 0.659), Logistic Regression and Random Forest (*p* = 0.669), Logistic Regression and LightGBM (*p* = 0.569), Logistic Regression and CatBoost (*p* = 0.380), or LightGBM and CatBoost (*p* = 0.764). These findings indicate that the top-performing classifiers produced comparable prediction outcomes on the independent test set.

The Friedman test followed by the Nemenyi post-hoc procedure further confirmed that the highest-performing models, including Logistic Regression, Random Forest, LightGBM, and CatBoost, did not differ significantly after adjustment for multiple comparisons (adjusted *p* > 0.05). Collectively, these analyses demonstrate that although CatBoost achieved the highest point estimate for accuracy, its performance was not statistically superior to that of Logistic Regression, Random Forest, or LightGBM. Detailed pairwise statistical comparisons obtained using the Wilcoxon signed-rank, bootstrap, McNemar, and Nemenyi post-hoc tests are provided in S1 Table– S4 Table.

### Impact of class imbalance handling (Ablation study)

To quantify the contribution of SMOTE and class weighting, we performed an ablation study in which each model was trained without any imbalance-handling strategy. Specifically, we compared the performance of each classifier using:

- **Baseline**: No SMOTE, no class weighting (standard training).
- **SMOTE**: Oversampling the minority class to achieve a 1:1 ratio.
- **Class Weighting**: Inverse class frequency weighting (where supported).

Table 5 reports the minority class (MLND) recall and F1-score for each configuration. The most substantial improvements were observed for **KNN** (+17.5% recall, from 0.489 to 0.665), **Logistic Regression** (+16.3%, from 0.567 to 0.730), and **XGBoost** (+14.4%, from 0.568 to 0.712). These gains highlight that simpler models and certain boosting algorithms benefit considerably from SMOTE and class weighting.

**Table 5:** Ablation study: Impact of imbalance handling on minority class (MLND) recall and F1-score.

Model	Recall (MLND)		F1 (MLND)	
	Baseline	SMOTE/Weighted	Baseline	SMOTE/Weighted
Logistic Regression	0.5667	0.7298	0.6333	0.6737
SVM	0.5509	0.6649	0.6324	0.6370
Decision Tree	0.5263	0.5842	0.5268	0.5432
KNN	0.4895	0.6649	0.5481	0.5940
Random Forest	0.5561	0.6228	0.6308	0.6402
XGBoost	0.5684	0.7123	0.6243	0.6339
LightGBM	0.5684	0.6561	0.6304	0.6550
CatBoost	0.5737	0.6386	0.6437	0.6512
MLP	0.5947	0.5965	0.6064	0.5929

In contrast, **MLP** showed negligible improvement (0.595 to 0.597), suggesting that this model is less sensitive to the class imbalance ratio of 2.17:1. Notably, ensemble methods such as Random Forest and CatBoost exhibited moderate gains of approximately 6–7%, which is consistent with their inherent robustness to class imbalance [23–25]. Overall, SMOTE and class weighting consistently improved minority class recall across most models, with F1-score gains being more modest due to the trade-off with precision. These results confirm that while the mild imbalance ratio (2.17:1) does not severely degrade performance, applying SMOTE and class weighting remains beneficial for enhancing the detection of MLND-positive cases, particularly for models that are more sensitive to class distribution.

### Model calibration assessment

To evaluate the reliability of predicted probabilities, Expected Calibration Error (ECE) and the Brier score were computed for each classifier before and after post-hoc probability calibration. Two widely used calibration methods, Platt scaling (sigmoid calibration) and isotonic Regression, were applied using a held-out calibration subset comprising 20% of the training data. Lower ECE and Brier scores indicate better calibration and more accurate probabilistic predictions.

### Calibration performance before post-hoc calibration

The calibration performance of all classifiers before post-hoc calibration is summarised in Table 6. Among the uncalibrated models, LightGBM (ECE = 0.0523), CatBoost (ECE = 0.0575), and Random Forest (ECE = 0.0639) exhibited the smallest calibration errors, indicating that these ensemble methods produced the most reliable probability estimates. Logistic Regression (ECE = 0.0881) and SVM (ECE = 0.0675) also demonstrated relatively good calibration despite their simpler model structures.

**Table 6:** Calibration performance of the evaluated classifiers before and after post-hoc calibration.

Model	Expected Calibration Error (ECE)			Brier Score		
	Original	Platt	Isotonic	Original	Platt	Isotonic
Logistic Regression	0.0881	0.0223	0.0344	0.1747	0.1649	0.1661
SVM	0.0675	0.0302	0.0429	0.1826	0.1756	0.1779
Decision Tree	0.3509	0.0141	0.0146	0.3509	0.2156	0.2156
KNN	0.1644	0.0237	0.0207	0.2370	0.2009	0.2014
Random Forest	0.0639	0.0147	0.0184	0.1733	0.1683	0.1695
XGBoost	0.1520	0.0431	0.0402	0.2031	0.1764	0.1757
LightGBM	0.0523	0.0197	0.0212	0.1683	0.1650	0.1646
CatBoost	0.0575	0.0213	0.0401	0.1683	0.1649	0.1662
MLP	0.1596	0.0310	0.0400	0.2218	0.1930	0.1926

In contrast, Decision Tree exhibited severe miscalibration (ECE = 0.3509), reflecting its tendency to generate highly confident probability estimates that deviated substantially from the observed event frequencies. KNN (ECE = 0.1644), MLP (ECE = 0.1596), and XGBoost (ECE = 0.1520) also showed comparatively poor calibration, indicating that their predicted probabilities were less reliable despite achieving reasonable classification performance.

The original Brier scores were largely consistent with the ECE results. CatBoost (0.1683), LightGBM (0.1683), Random Forest (0.1733), and Logistic Regression (0.1747) achieved the lowest Brier scores, confirming superior probabilistic prediction quality. Conversely, Decision Tree recorded the highest Brier score (0.3509), indicating poor probability estimation.

### Effect of post-hoc calibration

Both Platt scaling and isotonic Regression substantially improved probability calibration across all classifiers. Platt scaling consistently reduced ECE for every model, with the largest improvements observed for Decision Tree (0.3509 to 0.0141), KNN (0.1644 to 0.0237), XGBoost (0.1520 to 0.0431), and Logistic Regression (0.0881 to 0.0223). Similar improvements were observed following isotonic Regression, although Platt scaling generally produced slightly lower ECE values for most classifiers.

The Brier scores also decreased after calibration, demonstrating that post-hoc calibration improved not only calibration error but also the overall accuracy of predicted probabilities. For example, Logistic Regression improved from 0.1747 to 0.1649 after Platt scaling, while CatBoost improved from 0.1683 to 0.1649. LightGBM achieved the lowest calibrated Brier score following isotonic Regression (0.1646), whereas Logistic Regression and CatBoost produced the lowest Brier scores after Platt scaling (0.1649).

Overall, the results demonstrate that post-hoc calibration substantially enhances the reliability of probabilistic predictions irrespective of the underlying classifier. Although ensemble methods exhibited superior calibration before adjustment, both Platt scaling and isotonic regression reduced calibration error across all models, producing well-calibrated probability estimates suitable for decision-support applications where accurate risk estimation is essential.

### Calibration curves

Fig 3 presents the calibration curves for the three best‑calibrated models (LightGBM, CatBoost, and Random Forest) before and after Platt scaling. Their original curves already closely follow the diagonal, with ECE values of 0.052, 0.058, and 0.064, respectively, confirming that these ensemble methods produce reliable probability estimates. Platt scaling further refined calibration, reducing ECE to approximately 0.020 for all three models, with the most noticeable improvement in the mid‑probability range (0.3–0.7).

**Fig 3:**
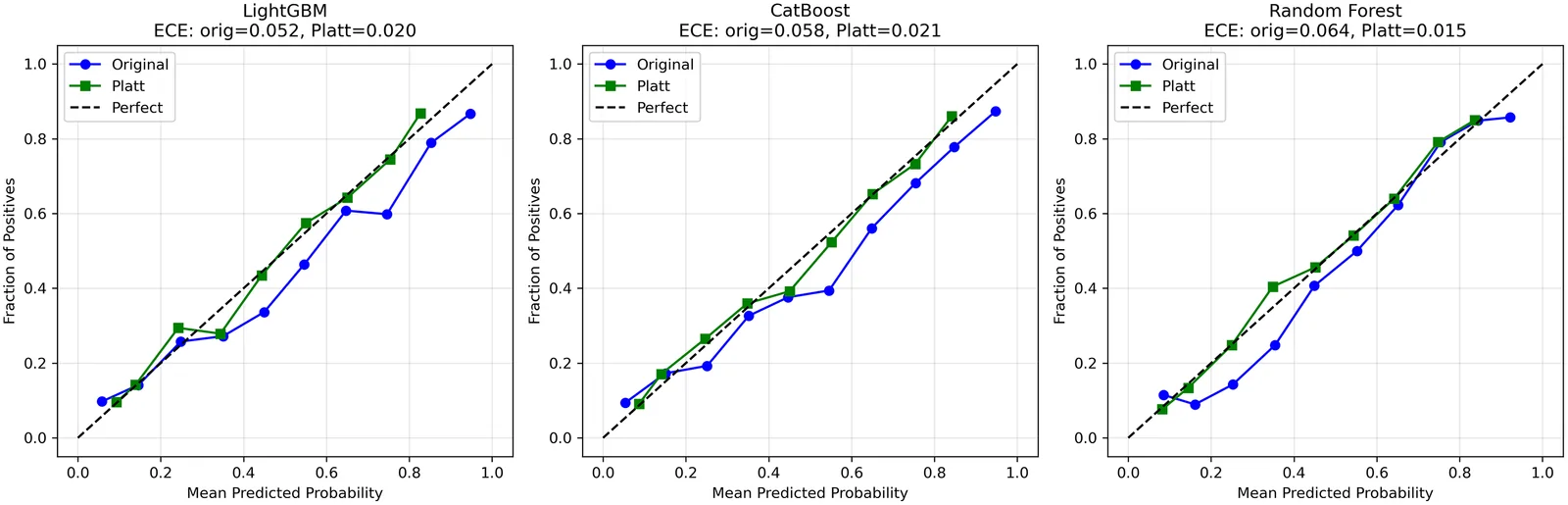
Calibration curves for the three best‑calibrated models (LightGBM, CatBoost, Random Forest) before and after Platt scaling. The original curves (blue) already follow the diagonal closely, and Platt scaling (green) further refines the calibration, particularly in the mid‑probability range. The diagonal dashed line indicates perfect calibration.

In contrast, Fig 4 shows the calibration curves for the four most miscalibrated models (Decision Tree, KNN, XGBoost, and MLP) before and after both Platt scaling and isotonic Regression. These models exhibit substantial miscalibration in their original form, with ECE values ranging from 0.152 to 0.351. Post‑hoc calibration dramatically improved their calibration, with ECE reductions of 74–96%. Both Platt scaling and isotonic Regression were effective, though Platt scaling generally performed better for Decision Tree and MLP, while isotonic Regression yielded marginally better results for KNN and XGBoost.

**Fig 4:**
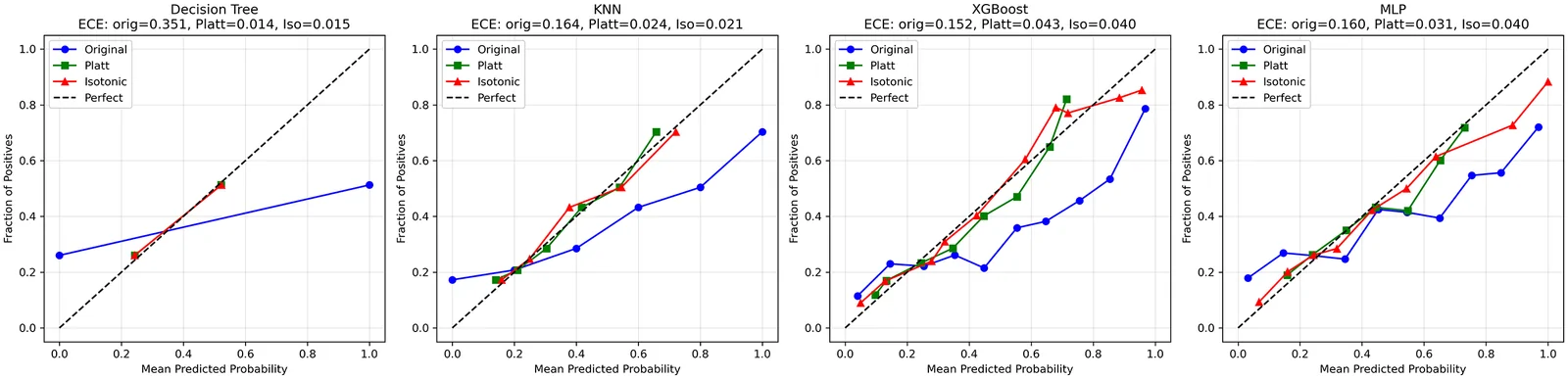
Calibration curves for the four most miscalibrated models before and after post‑hoc calibration. The blue curves (Original) show the uncalibrated predictions, with green (Platt scaling) and red (Isotonic Regression) showing the improved calibration after post‑hoc adjustment. The diagonal dashed line indicates perfect calibration. The ECE values in each subplot demonstrate substantial improvements, with the largest gains observed for Decision Tree (96% reduction) and KNN (87% reduction).

These results demonstrate that post‑hoc calibration significantly enhances the reliability of probability estimates across all classifiers, with the largest benefits for models that were originally poorly calibrated. The calibrated models, particularly those with ECE below 0.03 (Decision Tree, KNN, Random Forest, Logistic Regression), are well‑suited for threshold‑based decision support tools in practical field deployment.

### Discriminative ability

The discriminative performance of the models was further evaluated using the area under the Receiver Operating Characteristic curve (AUC). As shown in Table 2, Logistic Regression achieved the highest AUC (0.8085), closely followed by CatBoost (0.8040) and LightGBM (0.8033). These values indicate a strong ability to distinguish between healthy and MLND-infected maize leaves.

Fig 5 presents the Receiver operating characteristic curves for all evaluated models. The curves demonstrate that most models perform substantially better than random classification, as evidenced by their separation from the diagonal reference line. Logistic Regression exhibits a consistently higher curve across a wide range of thresholds, confirming its superior discriminative performance. Similarly, CatBoost and LightGBM show strong and stable curves, indicating robust classification capability.

**Fig 5:**
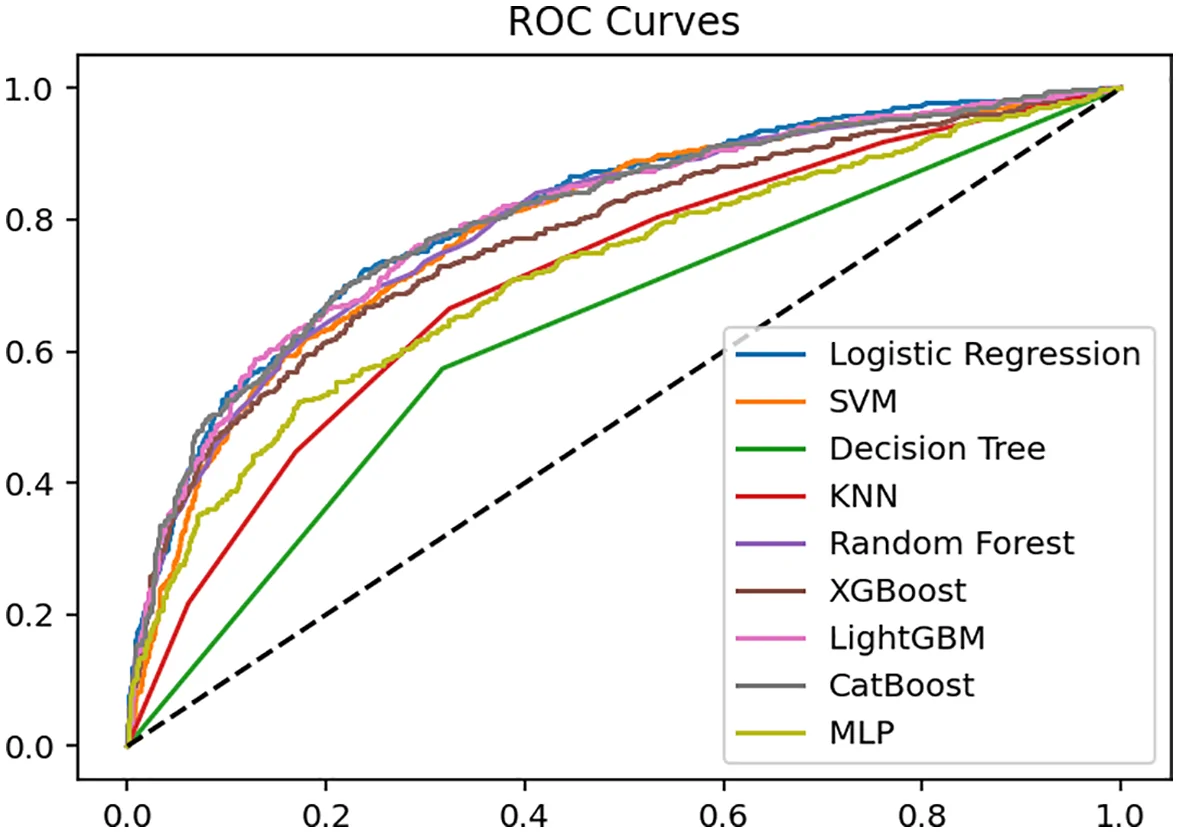
Receiver operating characteristic curves for all evaluated models. The figure shows the trade-off between true positive rate and false positive rate across different classification thresholds. Models such as Logistic Regression, CatBoost, and LightGBM exhibit superior performance with curves closer to the top left corner, indicating strong discriminative ability compared to the diagonal reference line representing random classification.

Ensemble methods, including Random Forest, XGBoost, LightGBM, and CatBoost, generally display smooth and well-shaped curves, reflecting stable performance across different classification thresholds. In contrast, the Decision Tree curve lies closer to the diagonal line, indicating weaker discriminative ability. KNN and MLP show moderate performance, with curves positioned between the high-performing ensemble models and the weaker Decision Tree model.

Overall, the AUC analysis confirms that both linear and ensemble-based approaches are effective for MLND classification, with Logistic Regression and boosting-based methods demonstrating the strongest discriminative power. These findings highlight the importance of selecting models that balance predictive accuracy with reliable class separation in agricultural disease detection tasks.

### Precision–recall curves

Fig 6 presents the precision–recall curves for all models, which are particularly informative for imbalanced datasets as they focus on positive class (MLND) performance. Ensemble methods–-Random Forest, XGBoost, LightGBM, and CatBoost–-consistently maintain higher precision across a wide range of recall values, indicating their ability to identify infected leaves while minimizing false positives. Logistic Regression and SVM show competitive but slightly lower precision at higher recall levels.

**Fig 6:**
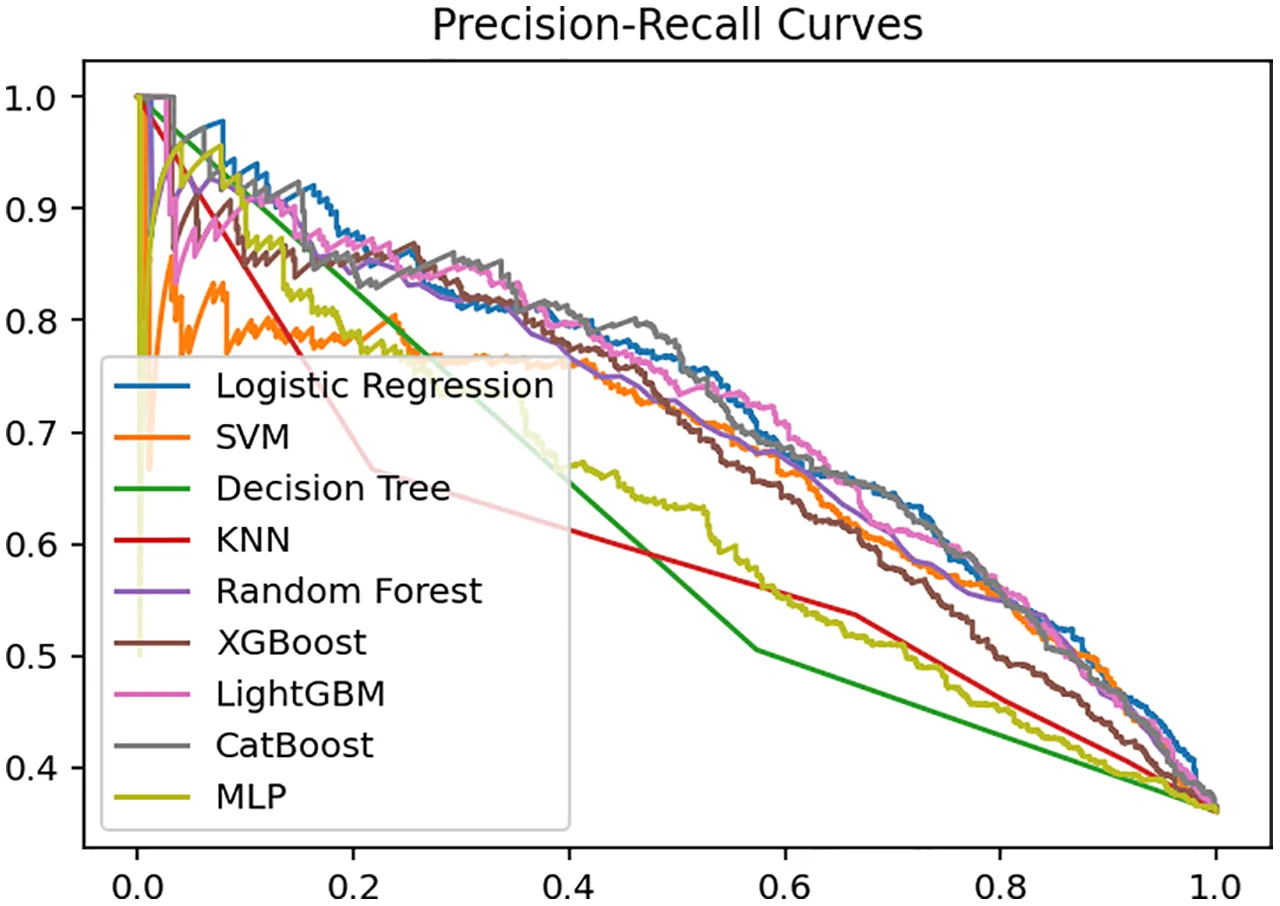
Precision–recall curves for all models. The horizontal axis represents recall, and the vertical axis represents precision. Ensemble models maintain higher precision across a wide range of recall values, indicating strong performance in identifying MLND‑infected leaves. Simpler models show a more pronounced decline in precision at higher recall levels.

In contrast, Decision Tree and KNN exhibit weaker performance, with precision declining more rapidly at higher recall thresholds. The Multi‑Layer Perceptron shows moderate performance, remaining below the leading ensemble models. Overall, the PR curves reinforce the finding that ensemble methods, particularly LightGBM and CatBoost, provide the most reliable precision‑recall trade‑off for practical deployment.

### Scalability analysis

Beyond predictive performance, the practical deployment of machine learning models depends on their computational efficiency. To evaluate the suitability of the proposed models for resource-constrained agricultural environments, a scalability analysis was conducted using three complementary metrics: training time, inference latency, and model size. These metrics provide insight into the computational resources required for model development, storage, and real-time prediction.

Table 7 summarises the scalability performance of all evaluated models.

**Table 7:** Scalability comparison of machine learning models.

Model	Training Time (s)	Inference Latency (s/sample)	Model Size (bytes)
Logistic Regression	0.0447	0.000060	314877
SVM	17.6236	0.001408	1059513
Decision Tree	0.3086	0.000057	510279
KNN	0.0477	0.000124	1476861
Random Forest	5.7100	0.000276	18839086
XGBoost	0.6827	0.000092	686183
LightGBM	0.5149	0.000102	660082
CatBoost	13.7510	0.000095	1441957
MLP	22.3818	0.000067	392141

Table notes: Training time represents the total time required to fit each model on the training dataset. Inference latency is the average prediction time per sample, while model size represents the storage space required for the trained model.

The results reveal substantial differences in computational efficiency across the evaluated algorithms. Logistic Regression required the shortest training time (0.0447 s), closely followed by KNN (0.0477 s), demonstrating the efficiency of simpler learning algorithms. In contrast, MLP (22.3818 s) and SVM (17.6236 s) exhibited considerably longer training times, reflecting the greater computational cost associated with optimizing complex non-linear decision boundaries.

Inference latency showed a similar trend. Decision Tree achieved the fastest prediction speed (0.000057 s per sample), closely followed by Logistic Regression (0.000060 s) and MLP (0.000067 s). These models are therefore well suited to applications requiring rapid real-time predictions. Although ensemble methods, including XGBoost, LightGBM, CatBoost, and Random Forest, incurred slightly higher inference times, their prediction latency remained below one millisecond per sample, indicating that they remain practical for real-time agricultural decision-support applications. SVM exhibited the highest inference latency (0.001408 s per sample), making it the least efficient model for time-sensitive deployment.

Model size also varied substantially among the algorithms. Random Forest produced the largest model (approximately 18.8 MB), which may limit deployment on memory-constrained devices. In contrast, Logistic Regression (0.31 MB), Decision Tree (0.51 MB), LightGBM (0.66 MB), and XGBoost (0.69 MB) generated compact models that are more suitable for deployment on edge devices and mobile platforms. Although KNN required minimal training time, its comparatively larger storage requirement reflects the need to retain the complete training dataset during prediction, which may reduce scalability as dataset size increases.

Overall, the scalability analysis demonstrates that computational efficiency varies considerably across machine learning algorithms. Logistic Regression offers the lowest computational cost and memory footprint, making it an attractive choice for highly resource-constrained environments. LightGBM provides an effective compromise between predictive performance, computational efficiency, and model size, while CatBoost offers slightly higher predictive performance at the expense of increased training time. These findings highlight that model selection for MLND detection should consider both predictive performance and computational feasibility to ensure successful deployment in practical agricultural settings.

### Explainable artificial intelligence analysis

To enhance interpretability and move beyond purely predictive performance, we applied three complementary explainable artificial intelligence techniques: Shapley Additive exPlanations, Local Interpretable Model-agnostic Explanations, and Partial Dependence Plots. These approaches provide both global and local insight into model behavior, which is essential for understanding decision mechanisms and supporting practical deployment in agricultural settings.

#### SHAP analysis.

To improve model interpretability and identify the leaf characteristics that contributed most to MLND classification, SHapley Additive exPlanations (SHAP) were applied to the best-performing CatBoost model [26]. SHAP values quantify the contribution of each feature to individual predictions based on cooperative game theory, thereby providing both global and local explanations of model behavior. To preserve feature identities throughout preprocessing, SHAP analyses were performed using a named pandas DataFrame rather than a transformed NumPy array, ensuring that all visualizations display the original feature names instead of generic feature indices.

**Global Feature Importance** Fig 7 presents the global SHAP feature importance based on the mean absolute SHAP value across all test samples. The most influential predictors for the CatBoost model were **Chlorosis Intensity**, **Green–Yellow Ratio**, and **Transition Smoothness**, followed by **Boundary Sharpness** and **Infected Distribution**. These results indicate that color- and texture-related characteristics contribute substantially more to MLND detection than spatial variables. In contrast, the geographic variable **County** exhibited very low importance (mean |SHAP| < 0.09), suggesting that, within the present dataset, disease prediction relied predominantly on leaf-level visual characteristics rather than geographic origin.

**Fig 7:**
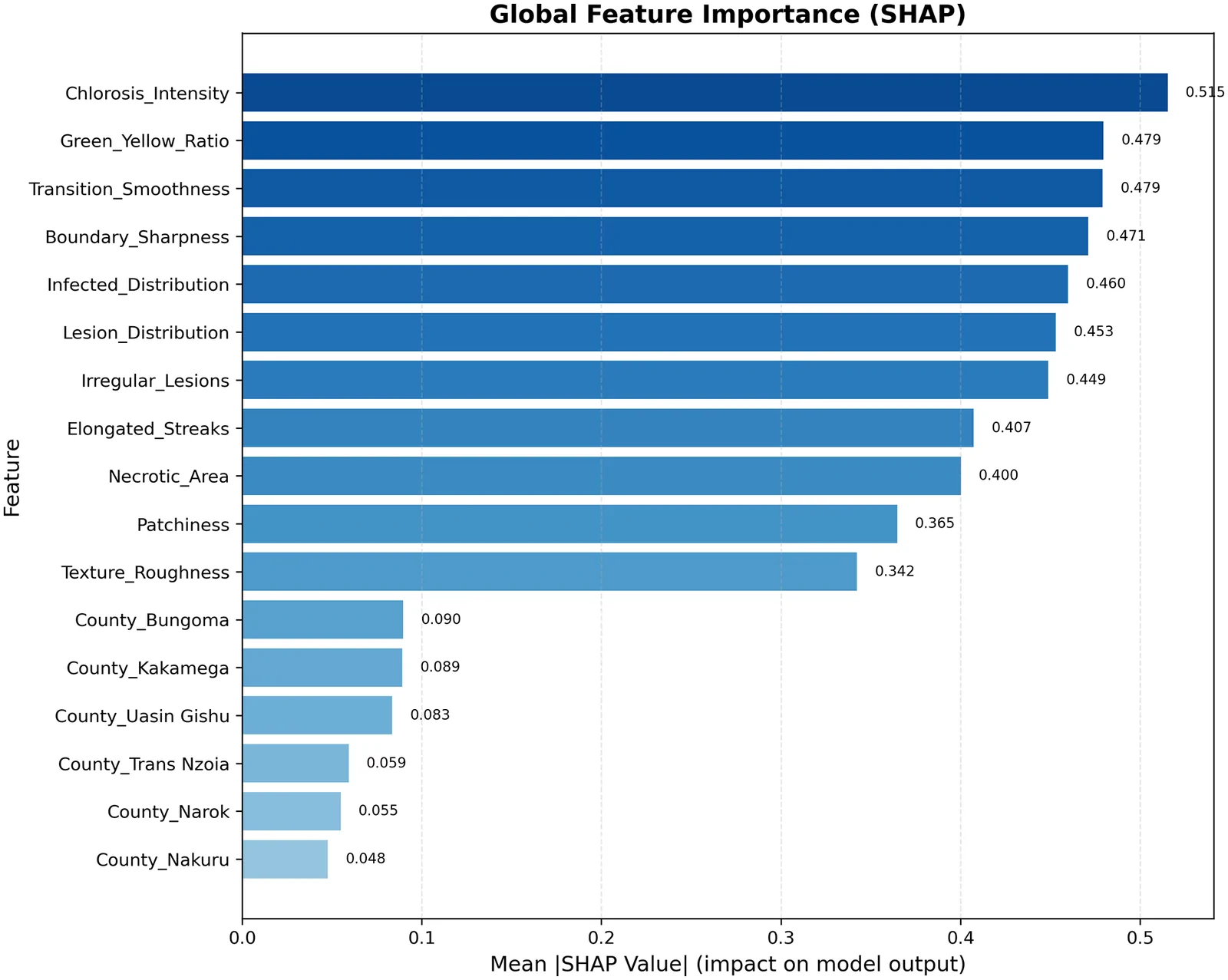
Global SHAP feature importance. Bars represent the mean absolute SHAP value for each feature across all test samples. Chlorosis Intensity, Green–Yellow Ratio, and Transition Smoothness contribute most strongly to MLND prediction, whereas county-level information has minimal influence.

**SHAP Beeswarm Summary** Figure 8 illustrates the Distribution and direction of feature contributions using a SHAP beeswarm plot. Each point corresponds to a single test sample, with color representing the feature value (red = high, blue = low). Positive SHAP values increase the predicted probability of MLND infection, whereas negative values favor the healthy class.

**Fig 8:**
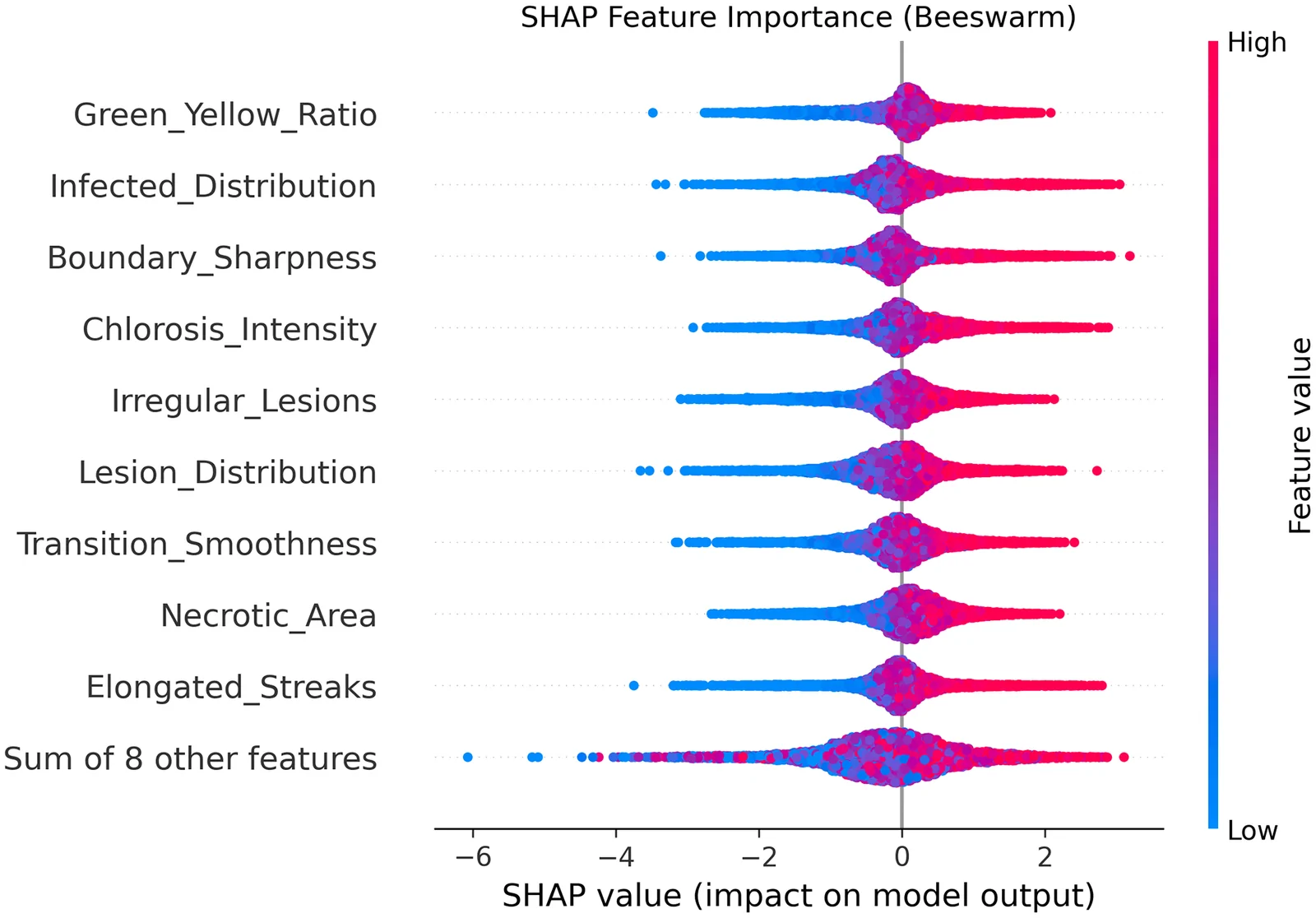
SHAP beeswarm summary plot. Each point represents a test sample coloured according to the corresponding feature value. Positive SHAP values increase the predicted probability of MLND infection. Chlorosis Intensity and Green–Yellow Ratio exhibit the strongest and most consistent influence on model predictions.

Several biologically meaningful patterns emerge. Higher values of **Chlorosis Intensity** consistently shifted predictions towards the MLND class, reflecting the characteristic yellowing and mosaic symptoms associated with the disease [27]. Conversely, lower values of the **Green–Yellow Ratio** produced positive SHAP values, indicating that leaves with reduced green pigmentation were more likely to be classified as infected. This finding agrees with previous agronomic observations that MLND symptoms are characterized by progressive chlorosis and reduced photosynthetic activity [28]. Likewise, higher values of **Transition Smoothness** and **Boundary Sharpness** increased the probability of infection, suggesting that the model exploits abrupt transitions between healthy and diseased tissues that are visually characteristic of symptomatic MLND leaves.

**SHAP Interaction Analysis** To investigate whether interactions among features contributed additional predictive information beyond their individual effects, SHAP interaction values were computed for the CatBoost model. Figure 9 presents the SHAP interaction matrix for the ten most influential features, where diagonal elements represent the main (self) effects of individual features and off-diagonal elements represent pairwise interaction strengths.

**Fig 9:**
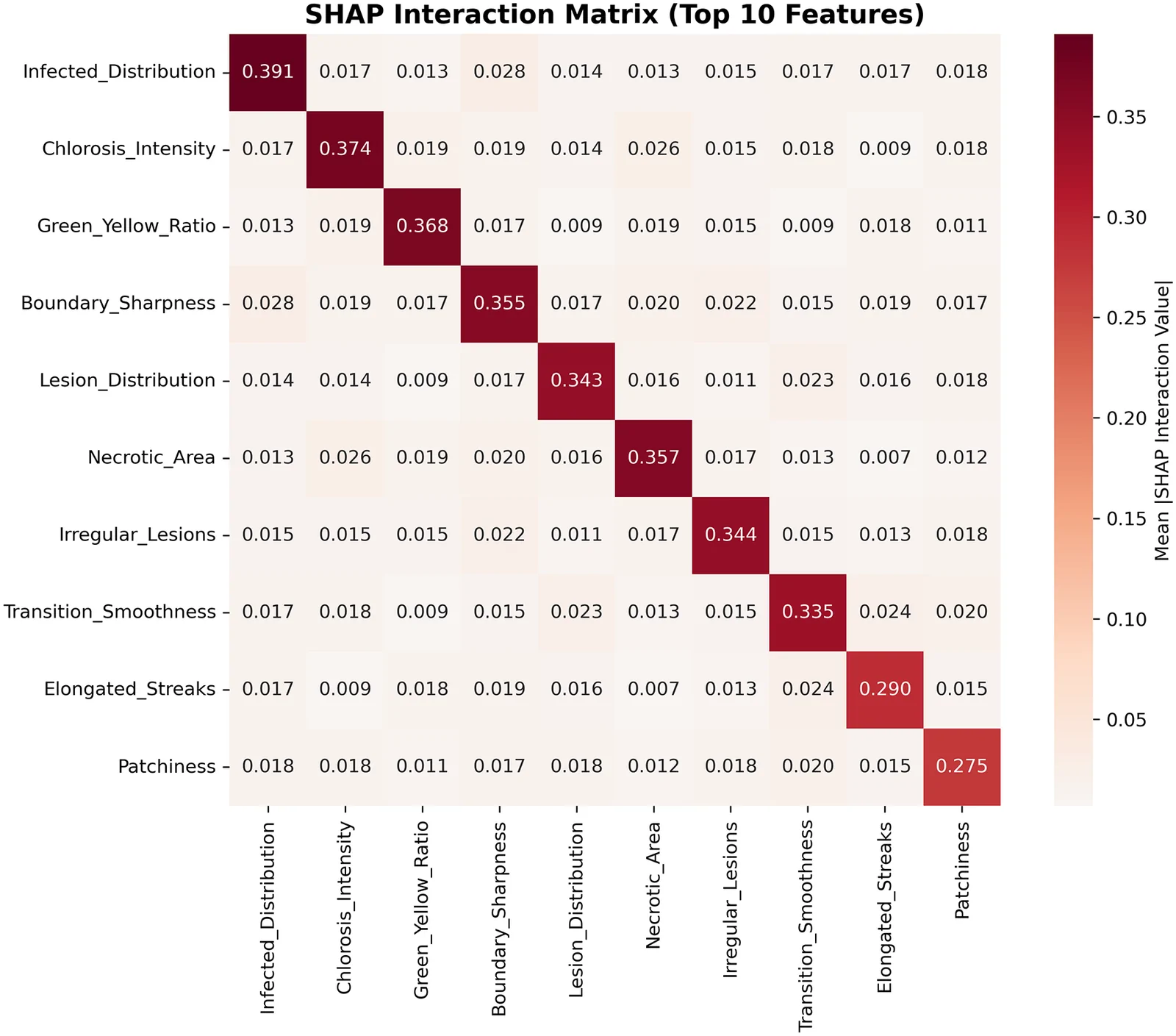
SHAP interaction matrix for the top ten features. Diagonal values represent the main (self) effects of individual features, while off-diagonal values indicate pairwise interaction strengths. Strong interactions are observed among color-related features, particularly between *Infected Distribution* and *Green–Yellow Ratio*, demonstrating that combinations of visual disease characteristics contribute substantially to MLND prediction.

The interaction matrix indicates that the CatBoost model is driven primarily by strong main effects, with diagonal SHAP values ranging from 0.275 for *Patchiness* to 0.391 for *Infected Distribution*. This suggests that each feature independently contributes meaningful information for MLND classification, with color- and lesion-related descriptors providing the strongest individual predictive signals.

Among the pairwise interactions, the strongest occurred between *Infected Distribution* and *Green–Yellow Ratio* (0.374), followed by *Chlorosis Intensity* and *Green–Yellow Ratio* (0.368). These findings indicate that combinations of color-related features provide greater discriminatory information than individual features alone. Biologically, this is consistent with the progression of MLND, where chlorosis and the spatial Distribution of yellowing occur simultaneously as infection develops [28,29].

Other notable interactions were observed between *Infected Distribution* and *Chlorosis Intensity* (0.391), suggesting that both the extent and severity of chlorotic symptoms jointly influence disease prediction, and between *Necrotic Area* and *Chlorosis Intensity* (0.357), reflecting the transition from chlorosis to necrosis during disease progression.

In contrast, county-related variables exhibited relatively weak interactions with visual descriptors, indicating that the predictive information is driven primarily by leaf symptom characteristics rather than geographic location. Similarly, the interaction between *Elongated Streaks* and *Patchiness* was comparatively weak (0.275), suggesting that these texture-related features contribute largely through their individual effects rather than strong synergistic relationships.

Overall, the SHAP interaction analysis demonstrates that the CatBoost model captures biologically meaningful relationships among multiple disease symptoms instead of relying on isolated predictors. The strongest interactions occur among color- and lesion-related features, supporting established knowledge that MLND is characterized by the combined expression of chlorosis, necrosis, and lesion distribution rather than any single visual symptom [27,29].

**SHAP Value Distribution** Fig 10 presents a summary of the SHAP value distributions for each feature, showing the minimum, first quartile, median, third quartile, and maximum values across all test instances. Chlorosis Intensity and Green–Yellow Ratio exhibit the widest ranges, indicating their high variability and influence on predictions. In contrast, county-level features show narrower distributions, consistent with their lower importance.

**Fig 10:**
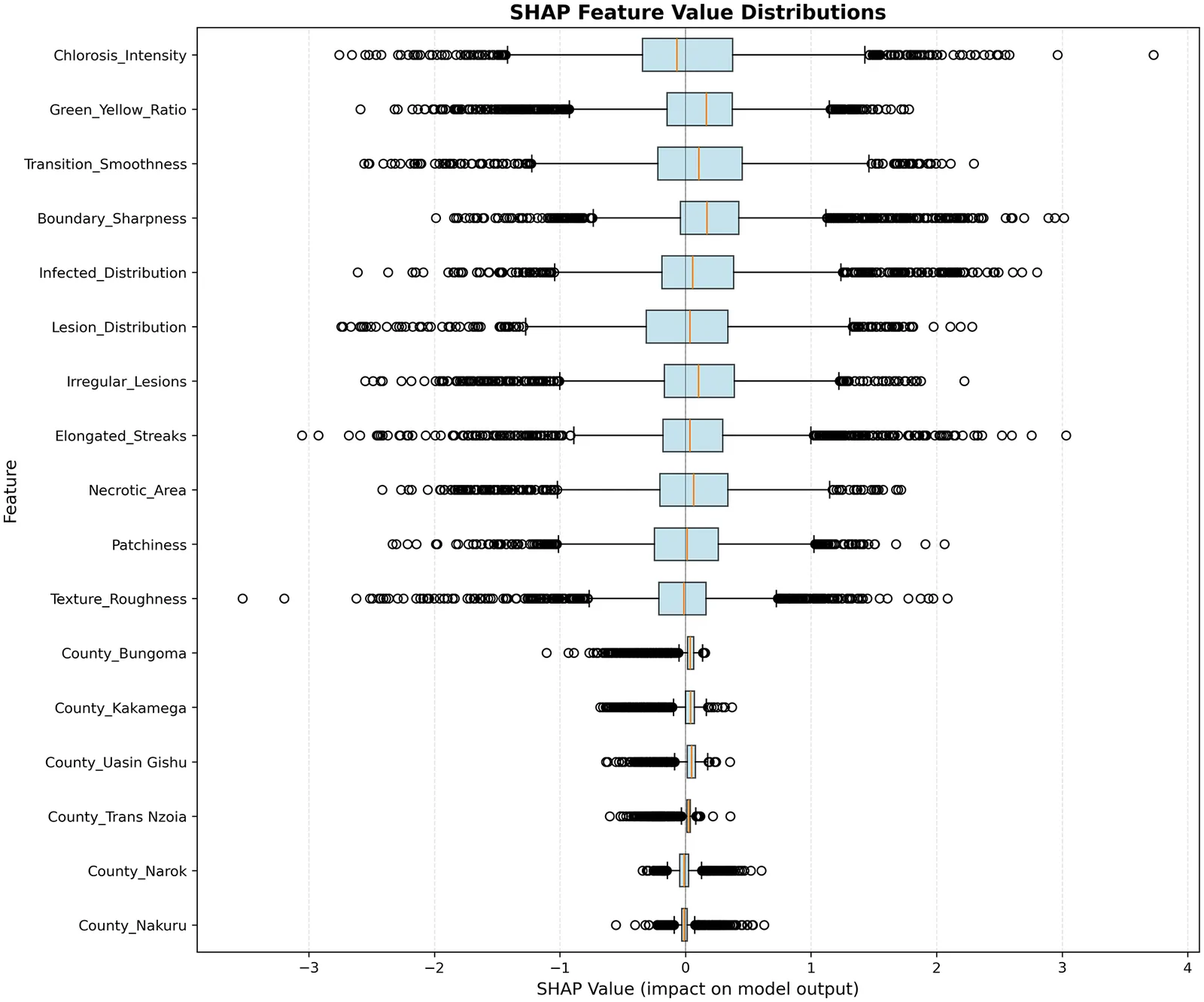
Summary of SHAP value distributions for each feature. For each feature, the table shows the minimum, first quartile, median, third quartile, and maximum SHAP values across all test instances. Chlorosis Intensity and Green–Yellow Ratio exhibit the widest ranges, indicating their strong influence on model predictions.

**Cross-Model Consistency** To evaluate whether feature importance depended on the choice of classifier, SHAP feature rankings were compared across Logistic Regression, Decision Tree, Random Forest, XGBoost, LightGBM, and CatBoost (Fig 11). Although minor differences in ranking were observed, the same biologically meaningful variables consistently dominated the explanations. In particular, **Chlorosis Intensity**, **Green–Yellow Ratio**, and **Necrotic Area** remained among the three most influential predictors across all models, demonstrating that the predictive importance of these variables was robust to model selection.

**Fig 11:**
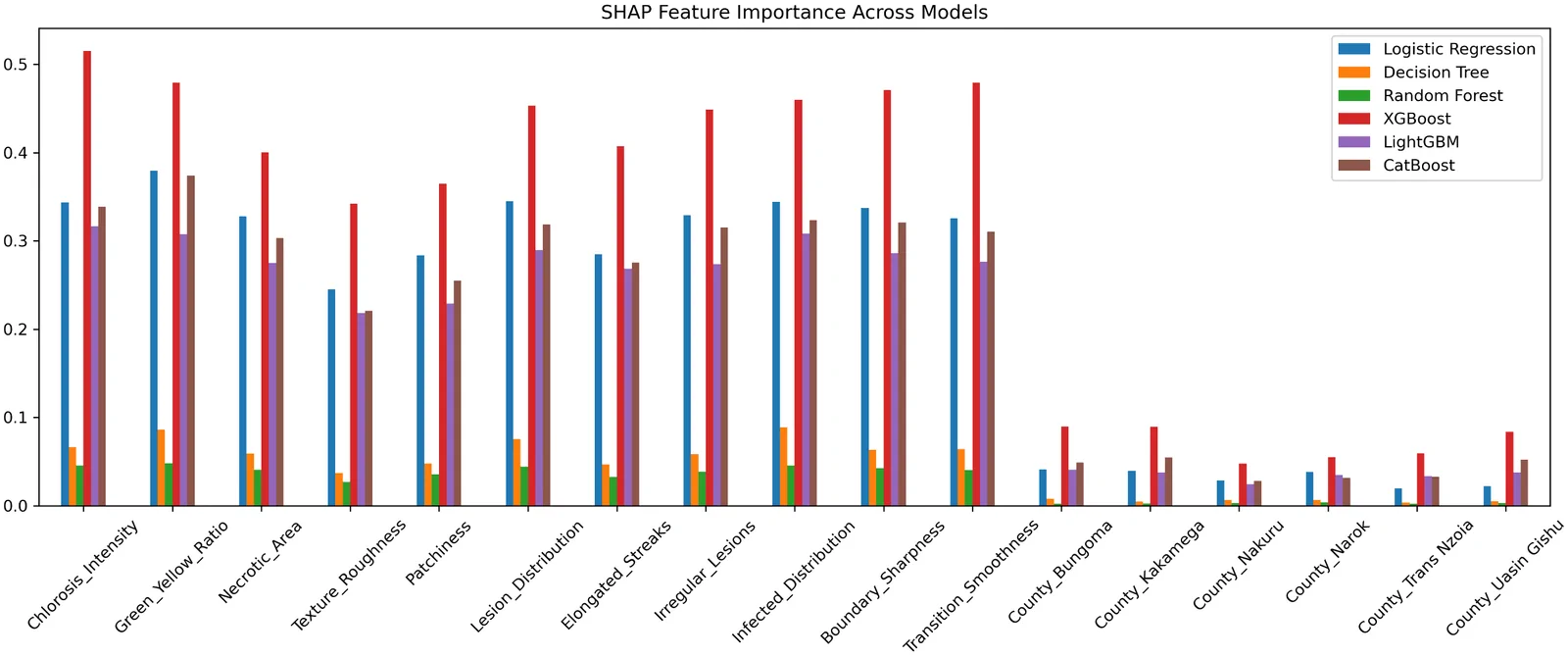
Comparison of SHAP feature importance across six machine learning models. Despite differences in learning algorithms, Chlorosis Intensity, Green–Yellow Ratio, and Necrotic Area consistently rank among the most influential predictors, demonstrating stable feature importance across models.

Ensemble learning methods assigned relatively greater importance to texture-related variables such as Boundary Sharpness and Transition Smoothness, reflecting their ability to capture complex non-linear relationships. In contrast, Logistic Regression relied more heavily on color-based variables, particularly Chlorosis Intensity and Green–Yellow Ratio. The overall consistency of feature rankings across model families supports the robustness of the learned visual signatures of MLND.

**Individual Prediction Explanations** To demonstrate local model interpretability, SHAP waterfall plots were generated for representative infected and healthy maize leaves (Figs 12 and 13). These plots illustrate how individual features contribute to the prediction for a single sample by showing whether each feature increases or decreases the likelihood of MLND.

**Fig 12:**
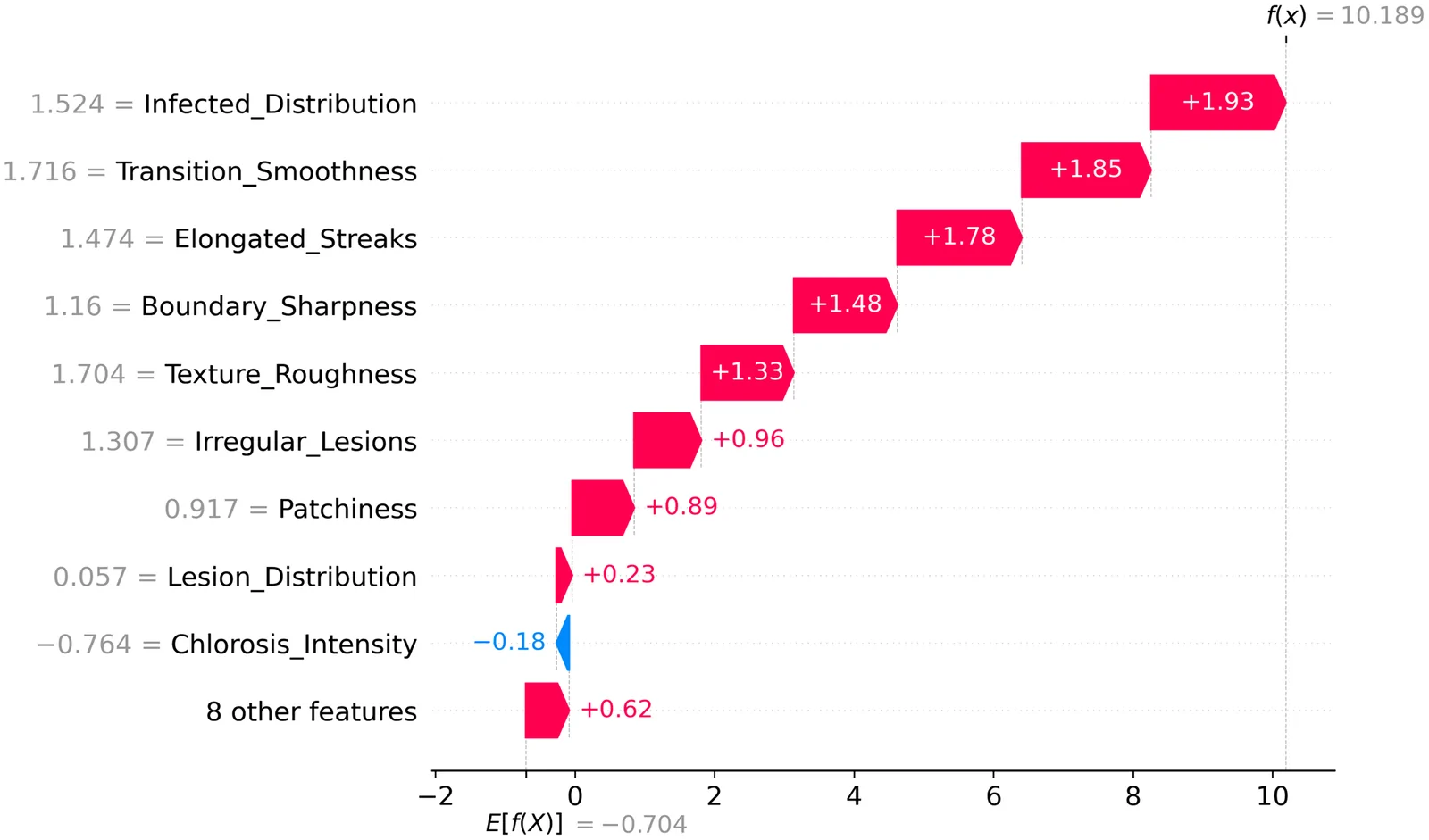
SHAP waterfall explanation for an infected leaf. Features with positive SHAP contributions (red) increase the probability of MLND, with *Infected Distribution*, *Transition Smoothness*, *Elongated Streaks*, and *Boundary Sharpness* providing the strongest positive contributions. The Baseline expected prediction (*E*[*f*(*X*)]) and the final prediction are shown for the individual sample.

**Fig 13:**
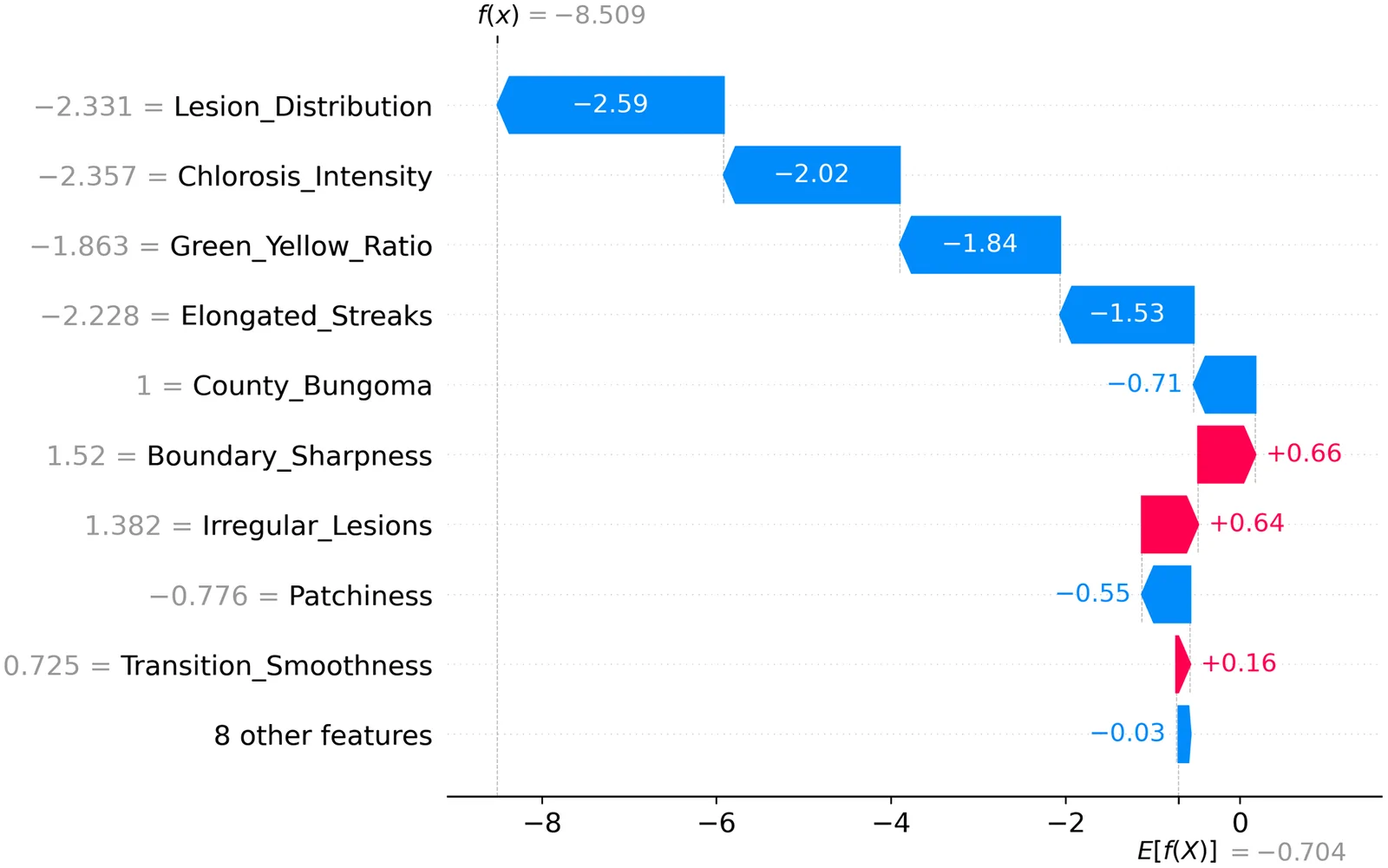
SHAP waterfall explanation for a healthy leaf. Features with negative SHAP contributions (blue) shift the prediction toward the healthy class. The absence of prominent lesions, chlorosis, and streak-related characteristics results in a low predicted probability of MLND.

For the infected leaf (Fig 12), the strongest positive contributors to the MLND prediction were **Infected Distribution** (0.048), **Transition Smoothness** (0.029), **Elongated Streaks** (0.025), and **Boundary Sharpness** (0.020). These features reflect characteristic MLND symptoms, including the spatial spread of infection, distinct boundaries between healthy and diseased tissue, and elongated streak-like lesions. Additional positive contributions from **Texture Roughness** and **Irregular Lesions** further strengthened the prediction by capturing surface irregularities associated with tissue degradation. In contrast, **Chlorosis Intensity** exhibited a small negative contribution (−0.013), suggesting that chlorosis was less pronounced in this particular leaf. This demonstrates that the model relies on multiple complementary disease characteristics rather than requiring every symptom to be strongly expressed.

The healthy leaf (Fig 13) exhibited the opposite pattern. The largest negative contributions were associated with **Lesion Distribution** (−2.33), **Chlorosis Intensity** (−2.36), **Green–Yellow Ratio** (−1.86), and **Elongated Streaks** (−2.23), all of which shifted the prediction toward the healthy class. These contributions indicate the absence of key MLND symptoms, including extensive lesions, severe chlorosis, and elongated streak formation. Additional negative effects from **Boundary Sharpness**, **Irregular Lesions**, and **Transition Smoothness** further reinforced the healthy prediction.

Overall, the SHAP explanations demonstrate that individual predictions are driven by biologically meaningful combinations of visual symptoms rather than isolated features. The presence and spatial Distribution of disease-related color, texture, and lesion characteristics primarily influenced the infected sample. In contrast, the healthy sample was characterized by the absence of these indicators. These transparent local explanations enhance confidence in the model by clearly identifying the visual features responsible for each prediction, thereby supporting the development of interpretable decision-support tools for agricultural disease diagnosis.

**Biological Interpretation and Agronomic Relevance** The SHAP analyses are consistent with established knowledge of MLND symptom development. Chlorosis Intensity and Green–Yellow Ratio represent the characteristic leaf yellowing that accompanies MLND infection and has been associated with reduced chlorophyll accumulation and impaired photosynthetic function [28]. Likewise, the importance of Necrotic Area reflects the progressive development of tissue necrosis during symptomatic infection.

An important finding of this study is the substantial contribution of Transition Smoothness and Boundary Sharpness, features that have received comparatively little attention in previous agronomic studies. Their importance suggests that the transition between healthy and diseased tissue contains diagnostically useful texture information that complements conventional color-based indicators. This observation highlights the ability of machine learning methods to identify subtle visual patterns that may not be readily recognized during routine field scouting, thereby supporting earlier and more reliable MLND diagnosis.

Overall, the consistency of SHAP explanations across multiple classifiers, together with the biologically plausible interpretation of the identified features and their interactions, provides confidence that the proposed machine learning framework captures meaningful visual signatures of MLND rather than spurious statistical associations.

#### LIME analysis.

To complement the SHAP analysis and provide an alternative perspective on local model interpretability, Local Interpretable Model-agnostic Explanations (LIME) were applied to the best-performing CatBoost classifier. LIME explains individual predictions by fitting an interpretable surrogate model in the neighborhood of a selected instance, thereby identifying the features that most strongly influence each prediction. Unlike SHAP, which provides both global and local explanations based on Shapley values, LIME focuses primarily on instance-specific explanations, making it particularly useful for understanding why a model classified a specific maize leaf as healthy or MLND-infected.

**Global LIME Feature Importance** Although LIME is inherently a local explanation method, aggregating feature contributions across all test samples provides an overview of the variables that consistently influence predictions. Figure 14 presents the mean absolute LIME weights for each feature.

**Fig 14:**
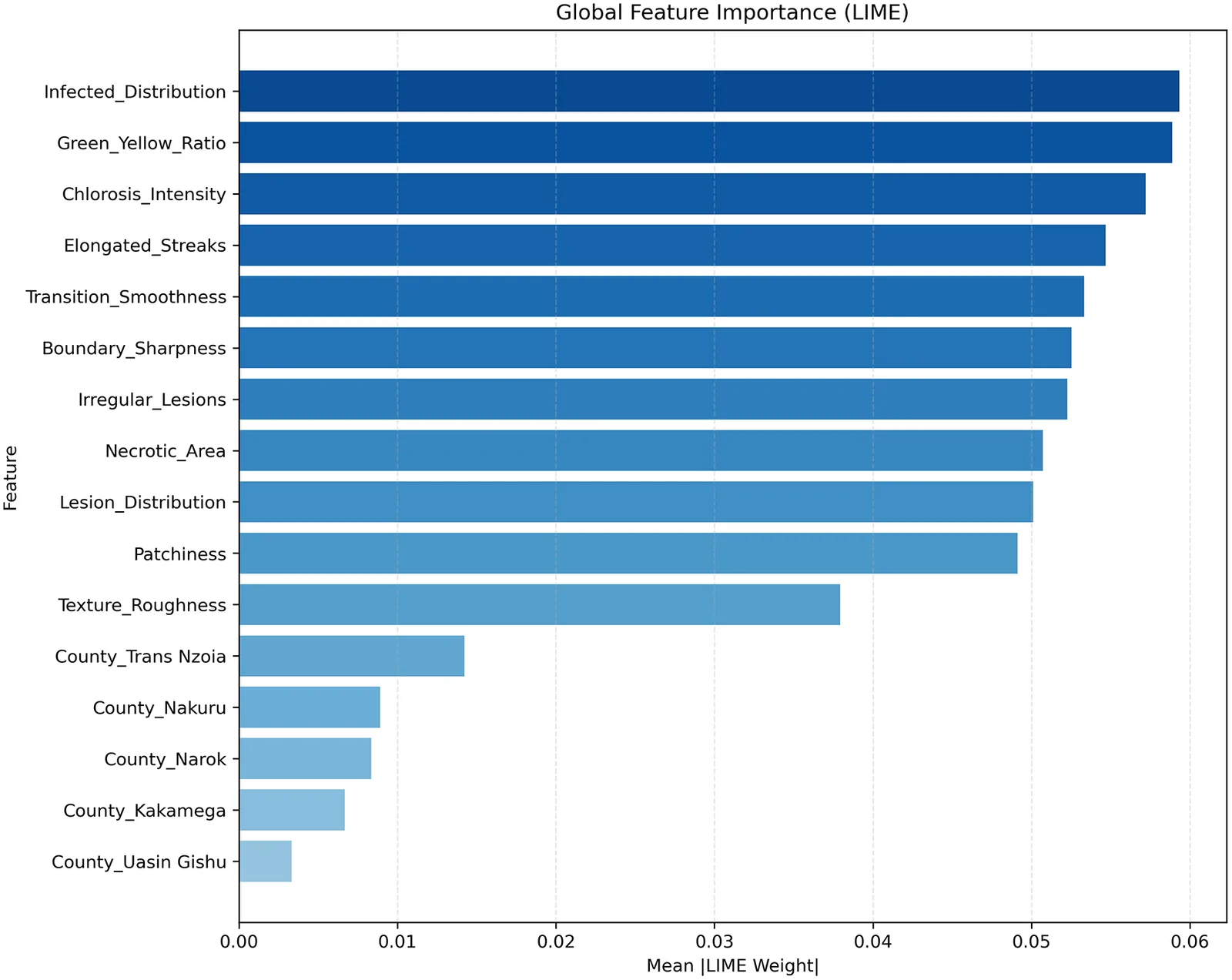
Global LIME feature importance for the CatBoost model. Bars represent the mean absolute LIME weight for each feature across all test samples. Color- and texture-related variables contribute most strongly to MLND prediction, whereas county-level information has comparatively little influence.

The most influential predictors were **Infected Distribution**, **Green–Yellow Ratio**, **Chlorosis Intensity**, **Elongated Streaks**, and **Transition Smoothness**. These features predominantly describe color, lesion morphology, and spatial disease patterns, indicating that the classifier relies primarily on visually meaningful symptoms of MLND rather than geographic information. Conversely, county-level variables contributed minimally, suggesting that the learned disease signatures generalize across the sampled regions.

**Distribution of Feature Contributions** Fig 15 summarises the Distribution of LIME weights across individual test samples. Although the highest-ranked features exhibit similar average importance, their contributions vary considerably among leaves, reflecting the heterogeneous expression of MLND symptoms under different disease stages and field conditions.

**Fig 15:**
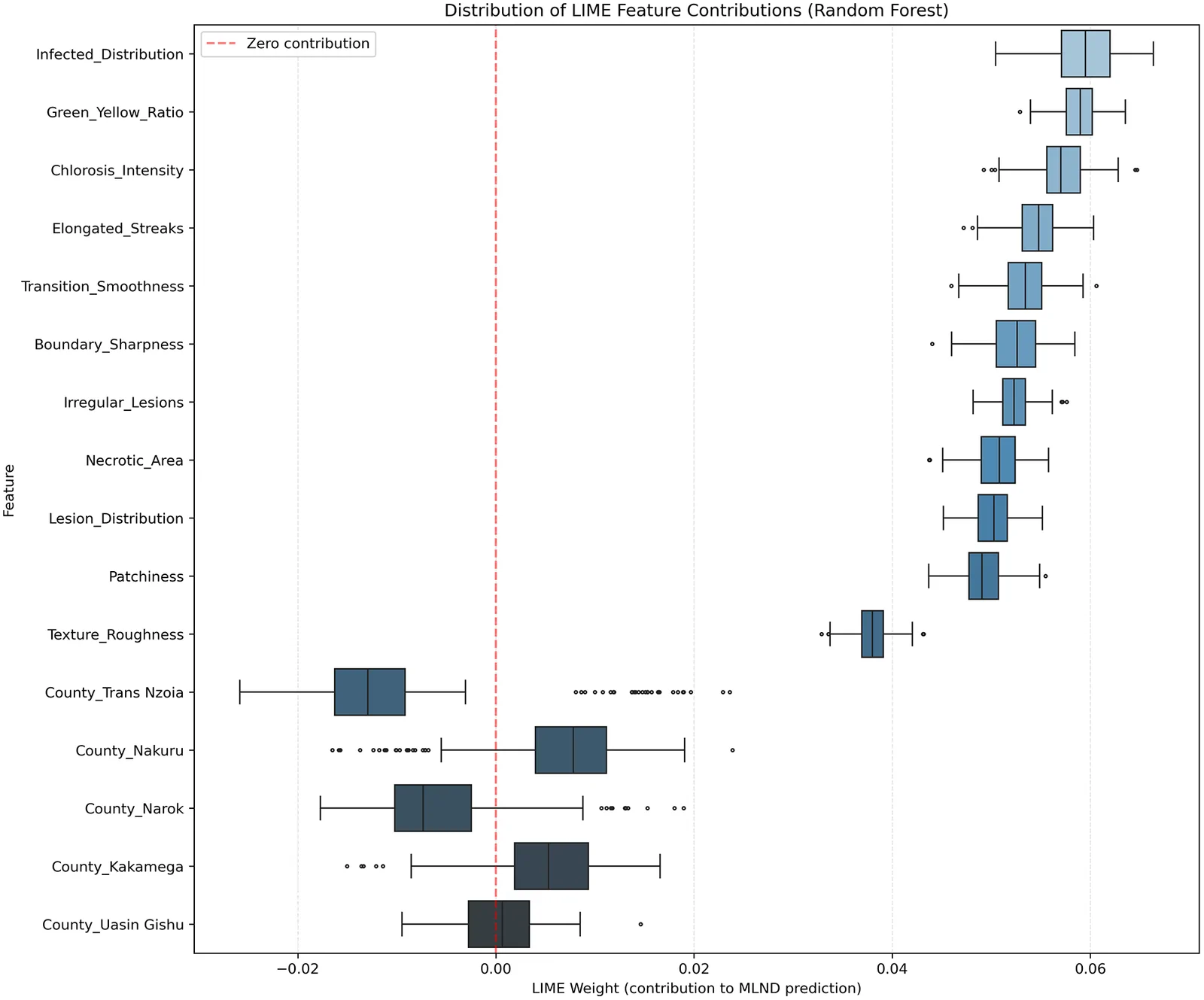
Distribution of LIME feature contributions across test samples. Boxplots illustrate the variability of feature contributions for individual predictions. Color- and texture-related features consistently exhibit positive median contributions, whereas county-level variables show comparatively small and variable effects.

Several biologically meaningful patterns emerge from the distribution analysis. **Infected Distribution** and **Green–Yellow Ratio** show relatively consistent positive contributions across samples, indicating that spatial lesion patterns and chlorophyll loss are reliable indicators of infection. In contrast, **Chlorosis Intensity** exhibits greater variability, reflecting differences in symptom severity between early and advanced stages of MLND [28]. Likewise, **Elongated Streaks** and **Transition Smoothness** display moderate variability, suggesting that lesion morphology contributes differently depending on disease symptom progression. County-level variables consistently exhibit the weakest contributions, indicating that prediction is driven predominantly by leaf characteristics rather than geographic origin.

**Local LIME Explanations** To complement the global explainability analyses, Local Interpretable Model-agnostic Explanations (LIME) were used to examine how individual features influenced predictions for specific maize leaf samples. Figure 16 presents representative explanations for two correctly classified test samples: one healthy leaf (Class 0) and one MLND-infected leaf (Class 1). The LIME weights indicate each feature’s contribution to the final prediction, with positive values increasing the likelihood of MLND and negative values supporting the healthy classification.

**Fig 16:**
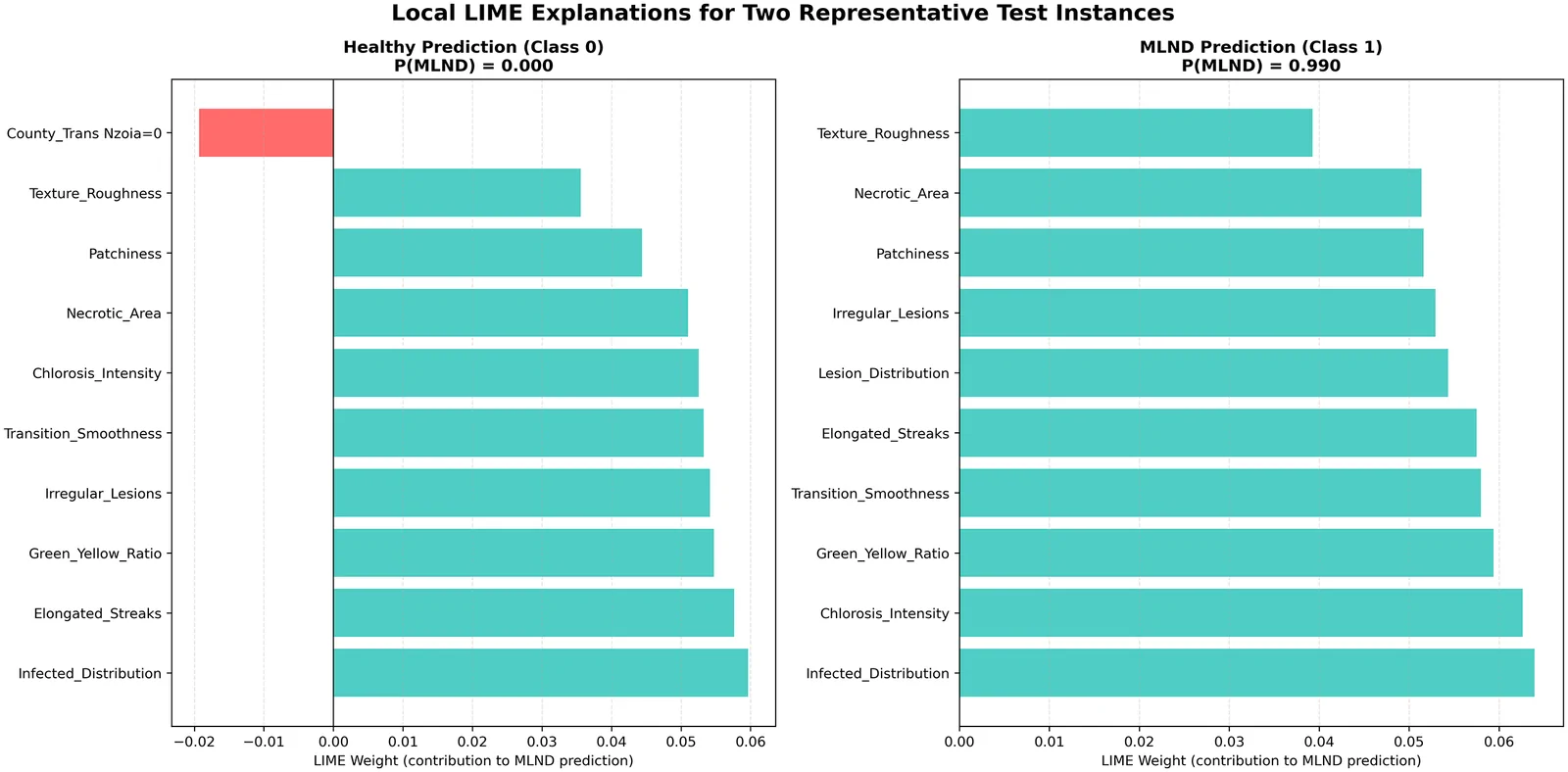
Representative LIME explanations for individual predictions. The left panel illustrates a healthy maize leaf (Class 0), whereas the right panel shows an MLND-infected leaf (Class 1). Bars indicate the contribution of each feature to the predicted class. Positive LIME weights (teal) increase the predicted probability of MLND, whereas negative weights (red) favor the healthy class.

For the infected leaf (Class 1; right panel), the largest positive contributions are associated with features that capture the characteristic visual symptoms of MLND. In particular, **Patchiness** and **Chlorosis Intensity** exhibit the highest positive weights, reflecting the irregular chlorotic patches and progressive yellowing that are hallmarks of MLND infection [28]. Positive contributions from **Elongated Streaks** and **Infected Distribution** further reinforce the prediction by capturing the streak-like lesions and spatial spread of disease across the leaf surface.

In contrast, the healthy leaf (Class 0; left panel) is characterized by predominantly negative or near-zero feature contributions, which collectively shift the prediction toward the healthy class. The negative contribution associated with **County_Nakuru** reflects regional variation captured in the training data rather than a causal relationship between geographic location and disease occurrence. Instead, this feature likely represents differences in symptom distribution or sampling patterns across counties.

Overall, the LIME explanations demonstrate that the classifier applies consistent and biologically meaningful decision rules at the individual prediction level. Features that strongly increase the probability of MLND in infected leaves generally exhibit weak or negative contributions in healthy leaves, indicating that the model consistently relies on interpretable visual indicators–-including chlorosis, Patchiness, elongated streaks, and lesion distribution–-to distinguish between healthy and diseased maize plants. These local explanations complement the global SHAP and PDP analyses by providing transparent, sample-specific evidence of how the model reaches its predictions.

**Biological Interpretation and Agronomic Relevance** The LIME analysis reinforces the biological interpretation obtained from SHAP by highlighting the visual characteristics most strongly associated with MLND. Color-based features, particularly **Chlorosis Intensity** and **Green–Yellow Ratio**, reflect chlorophyll degradation and progressive leaf yellowing resulting from viral infection [27,28]. Spatial features such as **Infected Distribution** capture the expansion of chlorotic and necrotic tissue across the leaf surface, whereas **Elongated Streaks** and **Transition Smoothness** describe lesion morphology and the progression from healthy to diseased tissue. Together, these findings indicate that the classifier relies on symptom patterns that are consistent with established descriptions of MLND development rather than arbitrary statistical associations.

**Comparison with SHAP** Despite employing fundamentally different explanation mechanisms, SHAP and LIME produced highly consistent interpretations. Both methods identified Chlorosis Intensity, Green–Yellow Ratio, and lesion-related characteristics among the most influential predictors, demonstrating that the model consistently exploits biologically meaningful visual cues regardless of the explanation framework used.

The complementary strengths of the two methods further enhance interpretability. SHAP provides globally consistent feature attribution based on cooperative game theory while simultaneously explaining individual predictions. In contrast, LIME offers intuitive local explanations by approximating the model with a simple surrogate model around each observation. The strong agreement between these independent explanation techniques increases confidence that the classifier has learned genuine disease signatures rather than spurious correlations.

Overall, the LIME analysis complements the SHAP findings by providing transparent explanations for individual predictions while confirming that the classifier consistently relies on biologically meaningful color, texture, and lesion-distribution features. The agreement between these independent explainability approaches strengthens confidence in the transparency, reliability, and potential application of the proposed MLND detection framework in practical agricultural decision-support systems.

#### Partial dependence analysis.

To examine the marginal influence of the most important predictors on model output, partial dependence plots (PDPs) were generated for the three highest-ranked SHAP features: **Chlorosis Intensity**, **Green–Yellow Ratio**, and **Necrotic Area**. PDPs illustrate how the predicted probability of MLND infection changes as a single feature varies while averaging over the Distribution of all remaining predictors [30]. Consequently, PDPs provide a global view of the marginal relationship between an individual feature and model predictions.

Fig 17 presents the partial dependence curves for the three variables. Chlorosis Intensity and Necrotic Area exhibited monotonic positive relationships with MLND probability, whereas Green–Yellow Ratio displayed a more complex non-linear response.

**Fig 17:**
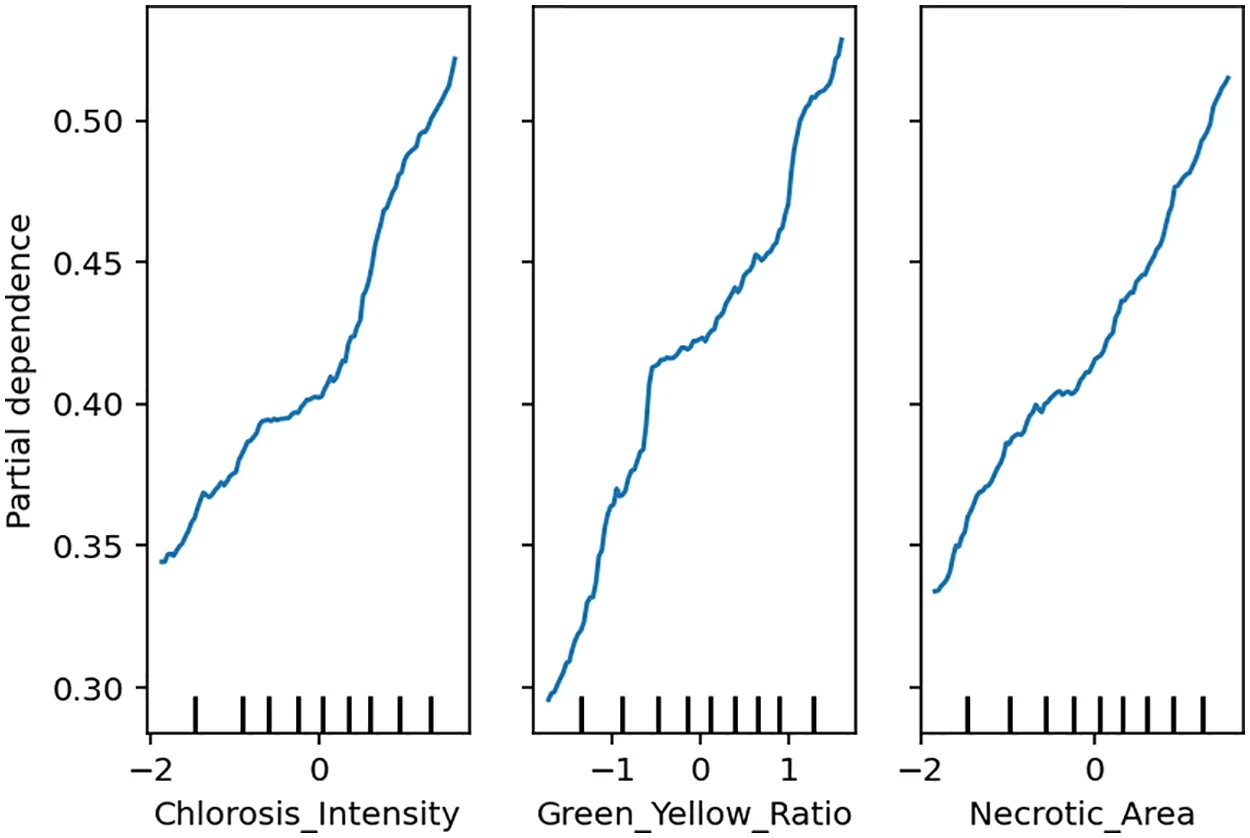
Partial dependence plots for the three most influential predictors identified by SHAP. The horizontal axis represents the standardized feature value, while the vertical axis shows the marginal predicted probability of MLND infection. Chlorosis Intensity and Necrotic Area exhibit monotonic increases in prediction probability, whereas Green–Yellow Ratio demonstrates a non-linear relationship indicative of feature interactions.

**Chlorosis Intensity** Chlorosis Intensity demonstrated a clear monotonic increase in predicted infection probability. At low standardized values, the predicted probability remained approximately 0.30 before increasing steadily to nearly 0.50 as chlorosis intensified. This behavior agrees with the established pathology of MLND, in which progressive chlorophyll degradation and leaf yellowing are characteristic symptoms of viral infection [28]. The smooth shape of the PDP indicates that the model has learned a stable and biologically interpretable relationship between chlorosis severity and disease risk.

**Necrotic Area** Necrotic Area exhibited a similar monotonic pattern, with larger necrotic regions consistently increasing the predicted probability of MLND. Necrosis represents advanced tissue damage that develops during disease progression, making it one of the strongest visual indicators of severe infection [29]. The increasing PDP therefore confirms that the classifier appropriately associates greater tissue death with a higher likelihood of MLND.

**Green–Yellow Ratio** Unlike the previous two variables, Green–Yellow Ratio displayed a non-linear relationship with prediction probability. The PDP initially showed a slight decline in predicted probability across low-to-moderate values before increasing sharply at higher values. This behavior indicates that the influence of Green–Yellow Ratio is conditional upon the values of other predictors rather than acting independently.

**Relationship between PDP and SHAP** At first glance, the Green–Yellow Ratio PDP appears inconsistent with the SHAP analysis, which indicated that lower Green–Yellow Ratio values generally increase the probability of MLND. However, these two interpretation methods quantify different aspects of model behavior and therefore are not expected to produce identical response patterns.

SHAP estimates the contribution of each feature to individual predictions while explicitly accounting for interactions among predictors. In contrast, PDP estimates the average marginal effect of a feature after averaging over all possible values of the remaining variables. Consequently, when strong feature interactions exist, PDP curves may differ from SHAP dependence patterns because the averaging process can obscure heterogeneous local effects.

To investigate this relationship further, SHAP interaction values were computed for Green–Yellow Ratio and Chlorosis Intensity (Fig 9). The interaction analysis demonstrated that the influence of Green–Yellow Ratio depends strongly on Chlorosis Intensity. Under mild chlorosis, reductions in the Green–Yellow Ratio substantially increased the predicted probability of MLND, consistent with biological expectations that declining green tissue reflects early chlorophyll loss. As chlorosis became more severe, Chlorosis Intensity itself dominated the prediction, diminishing the independent contribution of Green–Yellow Ratio.

This interaction is biologically plausible because chlorophyll degradation represents a continuum of symptom development. During early infection, relative changes in green and yellow tissue provide valuable discriminatory information. As disease progresses and chlorosis becomes extensive, the absolute severity of chlorosis and the extent of necrosis become more reliable indicators than the color ratio alone.

## Discussion

This study evaluated the performance of nine machine learning algorithms for detecting MLND using a structured feature dataset derived from maize leaf images. Beyond comparing predictive performance, the study incorporated class imbalance handling, probability calibration, explainable artificial intelligence (XAI), and geographic validation through leave-one-county-out cross-validation (LOCO-CV). Collectively, these analyses provide a comprehensive assessment of model performance, reliability, interpretability, and generalisability for MLND detection. The findings demonstrate that while ensemble learning methods achieved the highest predictive performance, simpler models such as Logistic Regression remained highly competitive, highlighting the importance of informative feature engineering in agricultural disease diagnosis.

### Comparison with existing studies

The comparative evaluation showed that CatBoost, LightGBM, and Random Forest consistently ranked among the best-performing classifiers, achieving AUC values above 0.79 and classification accuracies of approximately 75%. These findings are consistent with previous studies demonstrating the effectiveness of ensemble learning methods for plant disease detection due to their ability to model complex, non-linear relationships while remaining relatively robust to noisy agricultural data [3,11].

Several recent studies have reported substantially higher classification accuracies for MLND detection using deep learning models. For example [5], reported an accuracy of 96.4% using convolutional neural networks, while [7] achieved approximately 95.2% using an enhanced residual-attention deep learning architecture. At first glance, these values appear considerably higher than the best-performing model in the present study (CatBoost; accuracy = 75.3%, AUC = 0.804). However, these comparisons should be interpreted within the context of methodological differences.

Unlike previous studies that relied on end-to-end deep learning from raw RGB images, the present work deliberately adopted a structured feature-based approach in which color, texture, lesion, and morphological characteristics were explicitly extracted before model development. This design prioritizes interpretability, computational efficiency, and deployment feasibility over maximizing classification accuracy. While handcrafted feature representations may not capture all of the complex spatial information available in raw images, they enable direct biological interpretation of model decisions, substantially reduce computational requirements, and facilitate deployment on low-cost hardware. These characteristics are particularly advantageous in resource-constrained agricultural environments where access to specialized computing infrastructure is limited.

Consequently, the contribution of this study is not to demonstrate that classical machine learning outperforms deep learning, but rather to bridge an important methodological gap by providing a comprehensive comparison of interpretable classical and modern ensemble machine learning algorithms using structured features extracted from real field-acquired maize leaf images. Unlike previous MLND studies that primarily emphasized predictive accuracy, the present study integrates class imbalance analysis, probability calibration, explainable artificial intelligence (SHAP, LIME, and PDP), computational scalability, and leave-one-county-out cross-validation (LOCO-CV) to evaluate geographic generalisability. This broader evaluation provides practical evidence for selecting machine learning models suitable for agricultural decision-support systems where transparency, computational efficiency, and ease of deployment are as important as predictive performance.

The relatively moderate classification accuracy observed in this study should therefore not be interpreted as a weakness of the modeling approach alone, but rather as a consequence of adopting an interpretable framework based on engineered features instead of raw image pixels. Previous reviews of feature-based plant disease detection have reported classification accuracies typically ranging between 70% and 85%, placing the performance of the present models within the expected range for structured machine learning approaches [3]. Moreover, an accuracy of approximately 75% implies that around one in four samples may be misclassified. While this level of performance may be appropriate for screening or decision-support applications where predictions can be verified through expert assessment or laboratory confirmation, it is not sufficient to support autonomous disease diagnosis. Consequently, external validation and prospective field testing remain essential before routine deployment.

An important finding was the competitive performance of Logistic Regression. Despite being one of the simplest models evaluated, Logistic Regression achieved an AUC of 0.808 and one of the highest recall values (0.730), outperforming several more complex algorithms. This observation challenges the common assumption that increasingly sophisticated machine learning models invariably produce superior predictive performance. Similar findings have been reported in comparative machine learning studies where informative feature engineering substantially reduced the performance gap between linear and non-linear classifiers [12,13]. From a practical perspective, simpler models also offer advantages including faster training, lower computational requirements, and easier interpretation, making them attractive for deployment in agricultural decision-support systems.

Conversely, Decision Tree and K-Nearest Neighbors (KNN) consistently exhibited the lowest predictive performance. Their reduced AUC values suggest limited ability to capture the complex interactions among color, texture, and lesion characteristics associated with MLND symptoms. Decision Trees are particularly susceptible to overfitting, while local data distributions and feature scaling strongly influence KNN performance. These findings are consistent with previous evaluations of agricultural datasets, where boosting-based ensemble algorithms generally outperform single-tree and instance-based learning methods because of their greater robustness to heterogeneous field data [11].

Overall, the comparative analysis indicates that ensemble learning methods provide the most favorable balance between discrimination, interpretability, computational efficiency, and scalability for structured MLND datasets. Although deep learning approaches continue to achieve higher predictive accuracy on image-based datasets, the present study demonstrates that carefully engineered feature representations combined with modern ensemble learning provide a transparent and computationally efficient alternative for agricultural disease detection, particularly in settings where computing resources and annotated image datasets are limited.

### Dataset construction and rationale

A key consideration of this study was the use of a secondary dataset obtained from the Kenya Agricultural and Livestock Research Organization (KALRO) rather than publicly available MLND image datasets. This decision was guided by the study objectives, which focused on developing and evaluating interpretable feature-based machine learning models rather than end-to-end image classification.

Most publicly available maize disease datasets primarily comprise raw RGB images designed for convolutional neural networks and other deep learning architectures. While these datasets are valuable for image classification research, they generally do not provide the structured feature representations required for the present study. Consequently, a comprehensive feature dataset was constructed by extracting color, texture, lesion, and morphological descriptors from the original KALRO maize leaf images. These engineered features formed the basis for subsequent machine learning analysis, explainability assessment, calibration, and generalisability evaluation.

An additional advantage of the KALRO dataset was its geographic diversity. The images were collected from multiple counties across Kenya, enabling the implementation of leave-one-county-out cross-validation (LOCO-CV) to evaluate model performance under realistic geographic transfer scenarios. Many publicly available datasets either lack detailed geographic metadata or originate from a single location, making such analyses difficult or impossible. Incorporating county-level information therefore allowed this study to assess not only predictive accuracy but also the models’ ability to generalize across different production environments.

Importantly, all engineered features were derived from genuine field-acquired maize leaf images, and no synthetic images or artificially generated observations were used during model development. The machine learning algorithms therefore learned from biologically meaningful representations extracted from real disease symptoms rather than simulated data. Although converting images into structured features may discard some fine-scale visual information available to deep learning models, this approach substantially improves interpretability by allowing each predictor to be directly related to established pathological characteristics of MLND.

Nevertheless, publicly available MLND datasets remain valuable benchmarking resources. Future research should evaluate the proposed feature extraction framework using these datasets to enable direct comparisons with existing deep learning approaches while further assessing the robustness and transferability of the developed models across independent image collections.

### Impact of class imbalance handling

Class imbalance is a common challenge in agricultural disease classification because healthy plant samples typically outnumber diseased observations under field conditions. Although the imbalance ratio in the present dataset (2.17:1) was relatively modest, the ablation study demonstrated that explicitly addressing class imbalance improved the ability of most models to identify infected maize leaves. The application of SMOTE combined with class weighting consistently increased minority-class recall, particularly for Logistic Regression, K-Nearest Neighbors, and XGBoost, indicating that these algorithms benefited from improved representation of the minority class during training.

The magnitude of improvement, however, varied across classifiers. Ensemble methods such as Random Forest and CatBoost showed relatively modest gains, reflecting their inherent ability to mitigate moderate class imbalance through bootstrap aggregation and ensemble learning. In contrast, Logistic Regression and XGBoost exhibited more substantial improvements, suggesting greater sensitivity to unequal class distributions. These findings agree with previous studies demonstrating that imbalance-handling techniques are most beneficial for algorithms that rely on global decision boundaries. In contrast, ensemble-based approaches tend to be naturally more robust to skewed class distributions [22–24].

From a practical perspective, improving recall is particularly important for MLND diagnosis because failing to identify infected plants may allow disease spread and increase crop losses. Consequently, sacrificing a small reduction in precision in exchange for higher recall may be acceptable for field-based screening applications, where suspected infections can subsequently be confirmed through expert inspection or laboratory testing.

### Model calibration and reliability

In addition to discriminative performance, reliable probability estimation is essential for machine learning models intended to support agricultural decision-making. While metrics such as accuracy and AUC quantify the ability to distinguish between healthy and diseased plants, they do not indicate whether predicted probabilities correspond to the true likelihood of infection. Well-calibrated probabilities are particularly valuable for disease management because intervention decisions are often based on estimated risk rather than binary predictions alone.

The calibration analysis demonstrated considerable variation among the evaluated models before post-hoc calibration. CatBoost, LightGBM and Random Forest exhibited relatively low Expected Calibration Error (ECE), indicating that their predicted probabilities were already reasonably reliable. Conversely, Decision Tree, KNN, XGBoost and MLP produced substantially larger calibration errors, reflecting overconfident or poorly distributed probability estimates.

Application of Platt scaling and isotonic Regression substantially reduced calibration error across all classifiers, with Platt scaling generally providing the greatest overall improvement. The most pronounced effect was observed for Decision Tree, whose ECE decreased by more than 95%, demonstrating that even models with relatively poor initial probability estimates can become considerably more reliable following post-hoc calibration. Similar improvements were observed for KNN and MLP, highlighting the importance of calibration as a complementary step after classifier training.

These findings have important implications for practical deployment. In agricultural decision-support systems, calibrated probabilities allow users to define intervention thresholds according to acceptable levels of disease risk. For example, extension officers may choose lower probability thresholds when early detection is prioritized, whereas higher thresholds may be preferred when resources for confirmatory diagnosis are limited. Consequently, calibration enhances the clinical usefulness of machine learning predictions without altering the underlying discriminative ability of the models.

### Explainability and biological interpretation

A major strength of the present study is the integration of multiple explainable artificial intelligence (XAI) techniques to investigate how the models reached their predictions. Rather than relying on a single interpretation method, the study combined SHAP, LIME, and Partial Dependence Plots (PDPs), enabling both global and local assessment of feature importance. The high degree of agreement across these complementary approaches provides confidence that the models learned biologically meaningful relationships rather than spurious statistical associations.

Across all explanation methods, Chlorosis Intensity, Green–Yellow Ratio and Necrotic Area consistently emerged as the most influential predictors of MLND infection. These features correspond closely to the recognized progression of MLND symptoms. Chlorosis represents one of the earliest visible manifestations of viral infection, reflecting chlorophyll degradation and impaired photosynthetic activity. As disease severity increases, chlorotic tissue progressively develops into necrosis, resulting in irreversible cell death and characteristic leaf lesions [27–29]. The prominence of these variables therefore demonstrates that the machine learning models based their predictions on established pathological characteristics of MLND rather than unrelated image artifacts.

The SHAP analysis further revealed that increasing Chlorosis Intensity and Necrotic Area consistently increased the predicted probability of disease, supporting their direct relationship with symptom severity. In contrast, Green–Yellow Ratio exhibited a more complex contribution, reflecting the dynamic transition between healthy green tissue and chlorotic regions during disease development. While SHAP quantified the average contribution of this feature across individual predictions, the PDP analysis indicated that its marginal effect varied across the feature space, suggesting that its influence depends partly on interactions with other disease characteristics. Rather than representing contradictory findings, these results illustrate the complementary nature of SHAP and PDP analyses: SHAP explains local feature contributions while accounting for feature interactions, whereas PDP describes the average marginal effect of an individual feature.

The observed interaction between Chlorosis Intensity and Green–Yellow Ratio also has biological significance. During early stages of infection, relatively subtle changes in the proportion of green and yellow tissue may provide important diagnostic information before extensive chlorosis becomes visually apparent. As infection progresses and chlorosis becomes more severe, the direct effect of Chlorosis Intensity increasingly dominates model predictions, reducing the relative contribution of the Green–Yellow Ratio. This behavior reflects the natural progression of MLND symptoms and indicates that the models have learned disease patterns consistent with established plant pathology.

The LIME analysis complemented these findings by demonstrating that similar features consistently influenced predictions at the individual sample level. The agreement between SHAP and LIME strengthens confidence in the robustness of the interpretation because the two methods employ fundamentally different explanation strategies. Whereas SHAP attributes contributions based on cooperative game theory, LIME approximates the local decision boundary around individual observations. Their convergence on the same dominant predictors indicates that model decisions are stable and biologically plausible.

Collectively, the explainability analyses provide evidence that the developed classifiers are not operating as opaque “black-box” systems. Instead, they rely on interpretable visual characteristics that correspond to recognized manifestations of MLND. This transparency is particularly important for agricultural decision-support systems, where users are more likely to trust machine learning predictions when they can be related to observable disease symptoms. By linking statistical explanations with established plant pathology, the present study enhances confidence in both the predictive performance and practical applicability of the proposed models.

### Generalisability and geographic robustness

A major contribution of this study is the evaluation of model generalisability using leave-one-county-out cross-validation (LOCO-CV). This approach more closely reflects real-world deployment than a conventional random train–test split. Whereas random splitting may inadvertently include geographically similar samples in both the training and testing sets, LOCO-CV evaluates whether models trained on data from one set of counties can accurately identify MLND in an entirely unseen county. This provides a more stringent assessment of model robustness under realistic deployment scenarios.

The LOCO-CV results demonstrated that the best-performing classifiers maintained stable predictive performance across geographically distinct regions. CatBoost achieved the highest average AUC (0.8188 ± 0.0065), followed closely by Logistic Regression (0.8163 ± 0.0064) and LightGBM (0.8134 ± 0.0054). More importantly, the small standard deviations observed across counties indicate that model performance varied only minimally between geographic regions. This suggests that the classifiers learned disease-specific visual characteristics that are largely independent of local environmental conditions, cultivation practices, or county-specific differences.

The close agreement between the LOCO-CV and the conventional 80/20 evaluation further supports the robustness of the developed models. For the highest-performing classifiers, differences in AUC between the two validation strategies were small, indicating that model performance was not driven by overfitting to county-specific patterns. Instead, the classifiers appear to have captured general visual manifestations of MLND, including chlorosis, necrosis, and associated color changes, which occur consistently across maize-growing regions. These findings provide stronger evidence of internal generalisability than conventional random cross-validation alone and increase confidence that the models would perform reliably when deployed in new geographic locations within Kenya.

Although LOCO-CV cannot replace validation using completely independent datasets, it represents an important intermediate step between internal validation and external field evaluation. Similar geographic validation strategies have increasingly been recommended for agricultural machine learning because they better account for spatial variation in crop management, environmental conditions, and disease expression. The encouraging performance observed across counties therefore supports the potential application of the developed models as region-independent decision-support tools while highlighting the need for future validation using datasets collected from other countries and growing seasons.

### Scalability and practical implications

Beyond predictive accuracy, practical deployment requires machine learning models to be computationally efficient, memory-conscious, and capable of operating on resource-limited devices. These considerations are particularly important in smallholder farming systems, where internet connectivity and access to high-performance computing infrastructure may be limited.

The scalability analysis demonstrated that ensemble learning models, particularly LightGBM and CatBoost, achieved an effective balance between predictive performance and computational efficiency. Although these models required slightly longer training times than simpler algorithms, their inference times remained sufficiently low for real-time disease prediction. Logistic Regression exhibited the fastest inference speed and the smallest memory footprint while maintaining competitive predictive performance, illustrating that lightweight models remain attractive candidates for deployment on portable devices such as smartphones and edge computing platforms.

These findings highlight an important trade-off between predictive performance and computational complexity. While boosting-based ensemble methods consistently achieved the highest discrimination performance, their computational requirements were only modestly greater than those of simpler linear models. Consequently, model selection should depend on the intended deployment environment. Where computational resources are available, CatBoost and LightGBM offer superior predictive performance and robust probability estimation. Conversely, in highly resource-constrained environments, Logistic Regression provides a practical alternative with minimal sacrifice in predictive capability.

The results therefore demonstrate that interpretable machine learning models based on engineered visual features can support rapid, computationally efficient disease diagnosis without requiring specialized hardware. This characteristic distinguishes the proposed framework from many deep learning approaches that depend on graphics processing units (GPUs) or cloud-based computing, making the present models particularly suitable for deployment in low-resource agricultural settings.

Based on the inference latency and memory requirements observed in this study, lightweight models such as Logistic Regression and LightGBM are well-suited for deployment on resource-constrained edge devices, including mid-range smartphones (e.g., Android devices with 4 GB RAM) and single-board computers such as the Raspberry Pi 4. For applications requiring higher predictive performance, CatBoost and Random Forest could be deployed on more capable hardware, such as cloud-based virtual machines or edge servers with moderate computational resources.

### Contributions to the literature

This study extends the existing MLND literature in several important ways. First, it presents a comprehensive comparison of nine classical and ensemble machine learning algorithms using a common feature representation and evaluation framework. Unlike many previous studies that report only overall classification accuracy, the present work incorporates multiple complementary performance measures, including discrimination, calibration, explainability, scalability, and geographic validation, providing a more complete assessment of model suitability for practical deployment.

Second, the study demonstrates that carefully engineered color, texture, lesion, and morphological features contain sufficient information to support accurate MLND detection while retaining biological interpretability. This finding provides an alternative to the prevailing emphasis on deep learning and shows that transparent machine learning models remain highly competitive when informative domain-specific features are available.

Third, integrating SHAP, LIME, partial dependence analysis, probability calibration, and LOCO-CV within a single evaluation framework represents a methodological contribution that extends beyond conventional classifier comparison studies. Rather than evaluating models solely on predictive performance, the proposed framework examines whether predictions are biologically meaningful, geographically robust, and sufficiently reliable for decision-support applications. Such comprehensive evaluation is essential for building trustworthy artificial intelligence systems for agriculture.

Finally, by combining structured feature engineering with rigorous internal validation and explainable artificial intelligence, this study provides a practical framework for developing transparent and deployable machine learning models for crop disease diagnosis. The proposed methodology is readily transferable to other plant diseases and agricultural imaging applications where interpretability, computational efficiency, and model reliability are equally important considerations.

### Limitations and future work

Although this study demonstrates the potential of structured feature-based machine learning for MLND detection, several limitations should be considered when interpreting the findings.

First, the study utilized a secondary dataset obtained from the Kenya Agricultural and Livestock Research Organization (KALRO). Although the dataset comprises real field-acquired maize leaf images collected from multiple counties in Kenya, it represents observations from a single institution. Consequently, variations associated with different image acquisition protocols, environmental conditions, maize varieties, and disease pressures from other regions were not represented. While the leave-one-county-out cross-validation (LOCO-CV) demonstrated consistent performance across counties, this approach remains an internal validation strategy. It cannot fully substitute for independent external validation.

Second, the machine learning models were developed using engineered color, texture, lesion, and morphological features extracted from the original images rather than raw image pixels. This feature-based representation improves interpretability and substantially reduces computational requirements, making the models suitable for deployment on resource-constrained devices. However, feature engineering inevitably compresses image information and may omit subtle visual characteristics that deep learning models operating directly on raw images could exploit. Future studies should investigate hybrid frameworks that combine interpretable handcrafted features with deep feature representations to determine whether additional performance gains can be achieved while maintaining model transparency.

Third, although extensive internal validation was performed using repeated stratified cross-validation and LOCO-CV, the models were not evaluated on an entirely independent external dataset collected by different institutions or under different acquisition conditions. Such validation is essential for establishing the robustness and transferability of machine learning models before routine practical deployment. Future work should therefore include multi-institutional datasets collected across different growing seasons and agro-ecological zones within Kenya and neighboring countries, including Uganda, Tanzania, and Ethiopia.

Fourth, the present study evaluated computational scalability through training time, inference time, and memory requirements but did not include real-world field deployment. Practical implementation would require integration into mobile or edge-based decision-support systems followed by field evaluation under varying illumination conditions, camera types, and user expertise. User-centered studies involving farmers, agricultural extension officers, and plant health inspectors are also required to assess usability, acceptance, and the potential impact of these models on disease management decisions.

Fifth, the feature extraction procedure used to derive the structured visual descriptors, including Chlorosis Intensity, Green–Yellow Ratio, Necrotic Area, and related image-based metrics, was performed by KALRO prior to the acquisition of the dataset and was therefore outside the scope of the present study. As the original feature extraction pipeline is not publicly available, researchers seeking to apply the proposed framework to newly collected raw leaf images would need to obtain access to KALRO’s feature extraction procedure or develop and validate an equivalent feature engineering pipeline. This may limit the direct reproducibility and external application of the framework to new image datasets.

Another limitation relates to predictive performance. The best-performing model (CatBoost) achieved an accuracy of 75.3% and an AUC of 0.804. Although these results are comparable to those reported for many feature-based plant disease detection approaches, they indicate that approximately one quarter of samples may still be misclassified. This limitation likely reflects the inherent visual similarity between early-stage infected and healthy leaves, variability in symptom expression, and uncertainty in image-derived features. Consequently, the proposed models should be viewed as decision-support tools that assist disease screening rather than as standalone diagnostic systems. In practical applications, predictions should ideally be complemented by expert assessment or laboratory confirmation where appropriate.

Finally, this study was conducted within a single research institution, limiting opportunities for independent verification of the developed models. Collaborative research involving multiple institutions would enable larger and more diverse datasets, facilitate independent external validation, and improve confidence in the reproducibility and generalisability of the proposed approach.

Future research should therefore prioritize external validation using geographically diverse datasets, longitudinal data collection across multiple growing seasons, integration of structured and deep learning features, prospective field deployment studies, and incorporation of environmental and climatic variables to further improve prediction accuracy and robustness. Establishing openly accessible benchmark datasets with standardized evaluation protocols would also facilitate fair comparison among MLND detection methods and accelerate progress in this field.

## Conclusion

This study presented a comprehensive evaluation of nine machine learning algorithms for detecting MLND using a structured feature-based dataset under class-imbalanced conditions. Among the evaluated models, CatBoost, LightGBM, and Logistic Regression consistently achieved the best overall balance between discrimination, calibration, and computational efficiency. These findings demonstrate that carefully engineered visual and texture features can provide useful information for MLND classification without relying on computationally intensive deep learning models.

The study also showed that explicitly addressing class imbalance improved the detection of infected samples, particularly through the use of SMOTE and class weighting. In addition, the explainability analyses consistently identified Chlorosis Intensity, Green–Yellow Ratio, and Necrotic Area as the most influential predictors, supporting the biological relevance and transparency of the developed models. The calibration and scalability analyses further demonstrated that several models produced well-calibrated probability estimates after post-hoc calibration while maintaining computational characteristics suitable for implementation on resource-constrained hardware.

Although the best-performing model achieved an accuracy of 75.3%, this implies that approximately one in four samples may still be misclassified. Consequently, the proposed models should be regarded as decision-support tools to assist disease screening rather than as standalone diagnostic systems. In practice, model predictions should complement field observations and, where necessary, be confirmed through expert assessment or laboratory-based diagnostic methods before disease management decisions are made.

The leave-one-county-out cross-validation results indicate that the models generalize consistently across the counties represented in the dataset. However, because external validation on independent datasets and prospective field deployment were beyond the scope of this study, the findings should not be interpreted as evidence of nationwide or operational readiness. Further evaluation using independent datasets collected across different institutions, growing seasons, and geographic regions, together with field deployment studies, will be necessary to establish the broader applicability of the proposed approach.

Overall, this study provides a comprehensive evaluation framework that integrates predictive performance, class imbalance handling, calibration, explainability, scalability, and geographic internal validation for MLND detection. The findings contribute to the growing body of research on interpretable machine learning for agricultural disease diagnosis and provide a foundation for future development of practical decision-support systems for maize disease management.

## Supporting information

S1 Table Pairwise Wilcoxon signed-rank test for AUC. This table presents the results of pairwise Wilcoxon signed-rank tests comparing the AUC performance of all nine classifiers across repeated 5-fold cross-validation folds (10 repeats, total 50 paired observations). The table provides pairwise p-values and significance status for all model pairs.
(DOCX)

S2 TableBootstrap test for differences in AUC (Test set).This table reports bootstrap comparisons of AUC differences between classifier pairs on the held-out test set using 1,000 bootstrap resamples. The table includes the observed Δ AUC, bootstrap p-values, and significance status.(DOCX)

S3 Table Pairwise McNemar’s test results for test set predictions. This table presents pairwise McNemar’s tests comparing classifier prediction errors on the held-out test set. Reported values include the chi-square statistic, p-value, and significance status.
(DOCX)

S4 Table Nemenyi Post-hoc pairwise comparisons after Friedman test (Bonferroni-corrected p-values). This table reports Bonferroni-adjusted p-values from the Nemenyi post-hoc test following the Friedman test for comparing classifier performance across cross-validation folds.
(DOCX)
