# Supplementary material for: Comparative evaluation of machine learning algorithms for Maize Lethal Necrosis Disease detection under class imbalance
Source: PLoS One. 2026 Oct 23;21(10):e0349738. doi: 10.1371/journal.pone.0349738 (PMC13693939; doi:10.1371/journal.pone.0349738)
Supplement: Supplementary file 1 [file pone.0349738.s005.pdf]

Comments on PONE-D-26-21835

The topic presented in the manuscripts highlights an important and interesting considering the automated MLND detection in maize. However, there are a few major as well as minor concerns that needs to be fully addressed before moving forward.

Major Concerns

1. The manuscript highlights novelty in three key aspects: 1) dataset imbalance, 2) comparative study with numerous ML models, and 3) Utilization of large scale synthetic data to mimic MLND characteristics. (Line # 37-42). There is a need to address the following points which will surely highlight the novelty of the manuscript in a much clearer manner.
    - Firstly, majority of real-life datasets such as medical diagnostics or agriculture are normally imbalanced. So, in my opinion there is a need to include a few examples of publicly available balanced datasets existing in this research domain and studies with comparable performances.
    - Secondly, the manuscripts states that rigorous comparison of ML algorithms was conducted "bridging the gap between classical and modern approaches" in the MLND detection. In results section, the CatBoost achieved the highest accuracy 75.30%, which is particularly low considering the importance of reliability of disease detection and binary classification problem. Therefore, there is also a need to clearly pin point the key factors contributed in identifying the gaps between classical and modern approaches. Moreover, comparing these ML model performances with some recent modern approaches, particularly DL will enhance the scientific rigor of the study.
    - Thirdly, there is a stronger need to elaborate the protocol followed for obtaining the "large scale synthetic data", that is the core of this manuscript. Additionally, there are confusions regarding the synthetic data; whether this synthetic dataset is a publicly available or it was curated / generated as a part of this research study. If generated then elaboration of techniques will benefit the manuscript's novelty.
2. Line # 43-44. The manuscript states that "the study develops a structured and scalable dataset for MLND detection based on interpretable visual features". This line somewhat clarifies the point that the dataset was constructed as a part of this study. However, still there is a stronger need to provide detailed insights into the term "interpretable visual features", clearly elaborating the source of these visual features such as images or videos. Providing these details will surly enhance the understanding of potential readers.
3. Line # 46-47. The manuscript includes "resource-constrained environments" in the contributions section. Including a few examples of devices with specifications will establish the context properly.
4. The manuscript includes a heading "Model Development" whereas the study only includes comparative analysis of ML models, clearly lacking the novelty component particularly to the model development. In my opinion, experimentation with feature engineering and ML models will increase the scientific rigor of the study.
5. Hyper-parameters for MLP (no. of layers, epochs, optimizer, and learning rate), RF (no. of trees, depth), XGBoost (learning rate, maximum depth, scale_pos_weight), and SVM (kernel type, regularization parameter, and gamma) needs to properly elaborate along with empirical reasons

for the hyper-parameter values. Clearly mentioning these hyper-parameters will enhance the reproducibility aspect of this study.

6. The abstract mentions "Results showed that CatBoost (accuracy = 0.753), LightGBM (accuracy = 0.750), and Logistic Regression (AUC = 0.808) achieved the best overall performance". In results, the recall and F1-score of both LightGBM and CatBoost are quite close; considering these results along with training time and model size, suggesting one best fit model (with logical reasoning) for resource constrained settings will be extremely value-added.

Minor Concerns

1. Line # 85: "algorithms was implemented" needs to be corrected grammatically.
2. Mathematical definitions and elaborations of CatBoost and LightGBM needs to be added to increase the understanding of potential readers, as both these models are regarded as the best performers in the manuscript.
3. It is impressive that the author(s) have included the computational efficiency in terms of training time and inference latency. However, including a collective inference time for the whole test set will also give detailed insights to these ML models, particularly considering the facilities providing batch analysis.
4. One liner definitions of ML models are added alongside mathematical formulae which is a good thing. However, highlighting the reasons for choosing these specific ML models will enhance understanding of readers.
5. Resolution of figures needs to be improved keeping readability easier.
6. Some figures are cropped from the left side (looking into the page).

Overall the topic and content is good. But the manuscript requires considerable amendments for highlighting the novelty and contributions of the study. My recommendation is a major revision at the moment before moving forward.
