# Supplementary material for: Comparative evaluation of machine learning algorithms for Maize Lethal Necrosis Disease detection under class imbalance
Source: PLoS One. 2026 Oct 23;21(10):e0349738. doi: 10.1371/journal.pone.0349738 (PMC13693939; doi:10.1371/journal.pone.0349738)
Supplement: Supplementary file 3 [file pone.0349738.s002.docx]

**S2 Table.**

*Bootstrap Test for Differences in AUC (Test Set).*

| **Model 1** | **Model 2** | ∆**AUC** | **p-value** | **Significant (***α* = 0*.*05**)** |
| --- | --- | --- | --- | --- |
| Logistic Regression | SVM | 0.0199 | 0.5015 | No |
| Logistic Regression | Decision Tree | 0.1914 | 0.4845 | No |
| Logistic Regression | KNN | 0.1011 | 0.5015 | No |
| Logistic Regression | Random Forest | 0.0090 | 0.4995 | No |
| Logistic Regression | XGBoost | 0.0358 | 0.5055 | No |
| Logistic Regression | LightGBM | 0.0052 | 0.5385 | No |
| Logistic Regression | CatBoost | 0.0045 | 0.5584 | No |
| Logistic Regression | MLP | 0.0849 | 0.4885 | No |
| SVM | Decision Tree | 0.1716 | 0.4885 | No |
| SVM | KNN | 0.0812 | 0.4945 | No |
| SVM | Random Forest | -0.0108 | 0.4975 | No |
| SVM | XGBoost | 0.0159 | 0.5205 | No |
| SVM | LightGBM | -0.0147 | 0.5025 | No |
| SVM | CatBoost | -0.0154 | 0.4725 | No |
| SVM | MLP | 0.0650 | 0.4695 | No |
| Decision Tree | KNN | -0.0904 | 0.5035 | No |
| Decision Tree | Random Forest | -0.1824 | 0.4895 | No |
| Decision Tree | XGBoost | -0.1557 | 0.4945 | No |
| Decision Tree | LightGBM | -0.1863 | 0.4935 | No |
| Decision Tree | CatBoost | -0.1870 | 0.4865 | No |
| Decision Tree | MLP | -0.1066 | 0.5015 | No |
| KNN | Random Forest | -0.0920 | 0.4945 | No |
| KNN | XGBoost | -0.0653 | 0.4805 | No |
| KNN | LightGBM | -0.0959 | 0.4825 | No |
| KNN | CatBoost | -0.0966 | 0.4805 | No |
| KNN | MLP | -0.0162 | 0.5035 | No |
| Random Forest | XGBoost | 0.0267 | 0.5165 | No |
| Random Forest | LightGBM | -0.0038 | 0.5684 | No |
| Random Forest | CatBoost | -0.0046 | 0.5095 | No |
| Random Forest | MLP | 0.0758 | 0.4935 | No |
| XGBoost | LightGBM | -0.0306 | 0.5235 | No |
| XGBoost | CatBoost | -0.0313 | 0.5045 | No |
| XGBoost | MLP | 0.0491 | 0.4785 | No |
| LightGBM | CatBoost | -0.0007 | 0.8222 | No |
| LightGBM | MLP | 0.0797 | 0.4905 | No |
| CatBoost | MLP | 0.0804 | 0.4785 | No |
