# Supplementary material for: Comparative evaluation of machine learning algorithms for Maize Lethal Necrosis Disease detection under class imbalance
Source: PLoS One. 2026 Oct 23;21(10):e0349738. doi: 10.1371/journal.pone.0349738 (PMC13693939; doi:10.1371/journal.pone.0349738)
Supplement: Supplementary file 4 [file pone.0349738.s003.docx]

**S3 Table.**

*Pairwise McNemar's Test Results for Test Set Predictions.*

| **Model 1** | **Model 2** *χ*^2^ **p-value Significant (***α* = 0*.*05**)** | | | |
| --- | --- | --- | --- | --- |
| Logistic Regression | SVM | 5.985 | 0.0144 | Yes |
| Logistic Regression | Decision Tree | 68.343 | *<*0.0001 | Yes |
| Logistic Regression | KNN | 38.336 | *<*0.0001 | Yes |
| Logistic Regression | Random Forest | 0.183 | 0.6690 | No |
| Logistic Regression | XGBoost | 16.376 | 0.0001 | Yes |
| Logistic Regression | LightGBM | 0.325 | 0.5687 | No |
| Logistic Regression | CatBoost | 0.770 | 0.3802 | No |
| Logistic Regression | MLP | 20.376 | *<*0.0001 | Yes |
| SVM | Decision Tree | 49.124 | *<*0.0001 | Yes |
| SVM | KNN | 23.306 | *<*0.0001 | Yes |
| SVM | Random Forest | 7.955 | 0.0048 | Yes |
| SVM | XGBoost | 5.333 | 0.0209 | Yes |
| SVM | LightGBM | 8.556 | 0.0034 | Yes |
| SVM | CatBoost | 10.916 | 0.0010 | Yes |
| SVM | MLP | 9.623 | 0.0019 | Yes |
| Decision Tree | KNN | 6.694 | 0.0097 | Yes |
| Decision Tree | Random Forest | 79.133 | *<*0.0001 | Yes |
| Decision Tree | XGBoost | 24.249 | *<*0.0001 | Yes |
| Decision Tree | LightGBM | 76.810 | *<*0.0001 | Yes |
| Decision Tree | CatBoost | 80.574 | *<*0.0001 | Yes |
| Decision Tree | MLP | 16.435 | *<*0.0001 | Yes |
| KNN | Random Forest | 43.317 | *<*0.0001 | Yes |
| KNN | XGBoost | 6.144 | 0.0132 | Yes |
| KNN | LightGBM | 43.725 | *<*0.0001 | Yes |
| KNN | CatBoost | 47.438 | *<*0.0001 | Yes |
| KNN | MLP | 2.606 | 0.1065 | No |
| Random Forest | XGBoost | 19.129 | *<*0.0001 | Yes |
| Random Forest | LightGBM | 0.008 | 0.9306 | No |
| Random Forest | CatBoost | 0.195 | 0.6585 | No |
| Random Forest | MLP | 25.558 | *<*0.0001 | Yes |
| XGBoost | LightGBM | 24.556 | *<*0.0001 | Yes |
| XGBoost | CatBoost | 27.529 | *<*0.0001 | Yes |
| XGBoost | MLP | 0.598 | 0.4392 | No |
| LightGBM | CatBoost | 0.090 | 0.7642 | No |
| LightGBM | MLP | 26.214 | *<*0.0001 | Yes |
| CatBoost | MLP | 28.970 | *<*0.0001 | Yes |
