# Supplementary material for: Comparative evaluation of machine learning algorithms for Maize Lethal Necrosis Disease detection under class imbalance
Source: PLoS One. 2026 Oct 23;21(10):e0349738. doi: 10.1371/journal.pone.0349738 (PMC13693939; doi:10.1371/journal.pone.0349738)
Supplement: Supplementary file 5 [file pone.0349738.s004.docx]

S4 Table.

*Nemenyi Post-hoc Pairwise Comparisons After Friedman Test.*

| **Model 1** | **Model 2** | **Adjusted p-value** | **Significant (***α* = 0*.*05**)** |
| --- | --- | --- | --- |
| Logistic Regression | SVM | 0.1195 | No |
| Logistic Regression | Decision Tree | *<*0.0001 | Yes |
| Logistic Regression | KNN | *<*0.0001 | Yes |
| Logistic Regression | Random Forest | 1.0000 | No |
| Logistic Regression | XGBoost | *<*0.0001 | Yes |
| Logistic Regression | LightGBM | 1.0000 | No |
| Logistic Regression | CatBoost | 1.0000 | No |
| Logistic Regression | MLP | *<*0.0001 | Yes |
| SVM | Decision Tree | 0.0001 | Yes |
| SVM | KNN | 0.0496 | Yes |
| SVM | Random Forest | 1.0000 | No |
| SVM | XGBoost | 1.0000 | No |
| SVM | LightGBM | 1.0000 | No |
| SVM | CatBoost | 0.0864 | No |
| SVM | MLP | 1.0000 | No |
| Decision Tree | KNN | 1.0000 | No |
| Decision Tree | Random Forest | *<*0.0001 | Yes |
| Decision Tree | XGBoost | 0.2480 | No |
| Decision Tree | LightGBM | *<*0.0001 | Yes |
| Decision Tree | CatBoost | *<*0.0001 | Yes |
| Decision Tree | MLP | 1.0000 | No |
| KNN | Random Forest | 0.0005 | Yes |
| KNN | XGBoost | 1.0000 | No |
| KNN | LightGBM | *<*0.0001 | Yes |
| KNN | CatBoost | *<*0.0001 | Yes |
| KNN | MLP | 1.0000 | No |
| Random Forest | XGBoost | 1.0000 | No |
| Random Forest | LightGBM | 1.0000 | No |
| Random Forest | CatBoost | 1.0000 | No |
| Random Forest | MLP | 0.2239 | No |
| XGBoost | LightGBM | 0.8700 | No |
| XGBoost | CatBoost | *<*0.0001 | Yes |
| XGBoost | MLP | 1.0000 | No |
| LightGBM | CatBoost | 1.0000 | No |
| LightGBM | MLP | 0.0035 | Yes |
| CatBoost | MLP | *<*0.0001 | Yes |
