# Supplementary material for: Comparative evaluation of machine learning algorithms for Maize Lethal Necrosis Disease detection under class imbalance
Source: PLoS One. 2026 Oct 23;21(10):e0349738. doi: 10.1371/journal.pone.0349738 (PMC13693939; doi:10.1371/journal.pone.0349738)
Supplement: Supplementary file 6 [file pone.0349738.s006.docx]

**Response to the Academic Editor and Reviewers**

We sincerely thank the Academic Editor and all reviewers for their careful evaluation of our manuscript entitled **"Comparative Evaluation of Machine Learning Algorithms for Maize Lethal Necrosis Disease Detection Under Class Imbalance" (PONE-D-26-21835)**. We greatly appreciate the constructive comments and suggestions, which have substantially improved the quality, clarity, methodological rigor, and reproducibility of the manuscript.

In response to the reviewers' recommendations, we have undertaken a comprehensive revision of the manuscript. The major revisions include:

- Clarification of the dataset origin throughout the manuscript and correction of all inconsistencies regarding the data source.
- Revision of the Introduction to better justify the study novelty and clearly position our contribution relative to existing MLND and deep learning studies.
- Expansion of the Methods section to include detailed descriptions of the study design, variables, machine learning implementation, hyperparameter optimisation, class imbalance handling, calibration procedures, computational environment, and reproducibility considerations.
- Addition of an ablation study quantifying the impact of class imbalance handling.
- Inclusion of statistical comparisons using cross-validation summaries and confidence intervals, together with a more cautious interpretation of model differences.
- Regeneration of SHAP figures using the original feature names, inclusion of the missing SHAP beeswarm figure, and expansion of the explainability analysis with biological interpretation.
- Revision of the Discussion to include direct comparisons with previous MLND studies, justification of the structured feature-based approach, practical implications, limitations, and a more balanced conclusion.
- Revision of the Data Availability Statement and clarification of the use of secondary data obtained from the Kenya Agricultural and Livestock Research Organization (KALRO).
- Numerous improvements to figures, tables, grammar, formatting, references, and supplementary materials.

We believe that these revisions have addressed all of the reviewers' concerns and have substantially strengthened the manuscript.

**Responses to Reviewer #1**

We sincerely thank Reviewer #1 for the exceptionally thorough and constructive review. The comments significantly improved the scientific quality, clarity, and reproducibility of the manuscript. Each comment has been carefully considered and addressed as detailed below.

**Comment 1**

**Dataset Origin is Inconsistent and Unverifiable**

Three different descriptions of the dataset appear across three sections: the abstract describes it as "derived from real observations," the introduction calls it "a large-scale synthetic dataset designed to mimic real-world MLND characteristics," and the methods describe it as "secondary data obtained from KALRO." These are mutually exclusive descriptions. The authors must provide one clear, consistent statement of the dataset origin across all sections.

The Zenodo DOI does not resolve to any accessible record, directly violating PLOS One data availability policy. If the data was obtained from KALRO under a formal data use agreement, any sharing restrictions must be explicitly stated. If the dataset is synthetic, it should be freely shareable and the DOI must be corrected before re-submission.

**Response**

We thank the reviewer for identifying this important inconsistency.

The inconsistency resulted from an earlier draft of the manuscript that referred to a synthetic dataset. This was an oversight and has now been completely corrected throughout the manuscript.

The revised manuscript consistently states that the study used secondary data obtained from the Kenya Agricultural and Livestock Research Organization (KALRO) comprising 7,892 observations collected from field-acquired maize leaf images originating from multiple maize-growing counties in Kenya. No synthetic images or artificially generated samples were used in this study.

To improve clarity, we substantially revised the Abstract, Introduction, Study Design and Data Source, Dataset Construction and Choice, Discussion, Limitations, and Conclusion to consistently describe the dataset as secondary field-derived data obtained from KALRO.

We also expanded the manuscript to explain that the machine learning models operate on engineered visual, texture, lesion, and morphological features extracted from real maize leaf images, rather than raw image pixels.

The Zenodo DOI cited in the original submission is correct since it contains the real data from KALRO. I obtained permission from KALRO to make the dataset publicly available because it contains no sensitive or personally identifiable information.

**Comment 2**

**SMOTE vs. SMOTE-ENN Inconsistency**

The methods section describes SMOTE as the oversampling technique. The results section references SMOTE-ENN without prior definition. These are different methods. SMOTE-ENN combines synthetic minority oversampling with Edited Nearest Neighbours undersampling, which removes borderline and noisy majority class samples near decision boundaries. This cleaning step can substantially alter the training data distribution compared to SMOTE alone. If SMOTE-ENN was used, the methods section must describe it fully including the ENN component, the number of neighbours used, and the rationale for choosing the combined approach. The foundational SMOTE paper (Chawla et al., 2002) is absent from the reference list despite SMOTE being a core methodological component.

**Response**

We appreciate the reviewer highlighting this inconsistency.

The manuscript has been revised to consistently describe the class imbalance methodology used. The Methods section now clearly specifies:

- the exact resampling technique employed;
- the implementation details;
- the oversampling procedure;
- the nearest-neighbour parameter;
- the sequence of preprocessing operations;
- the application of resampling **only within the training folds** to prevent data leakage;
- and the rationale for combining resampling with class weighting.

The terminology has been made consistent throughout the manuscript, eliminating the previous ambiguity between SMOTE and SMOTE-ENN.

In addition, the foundational work by **Chawla et al. (2002)** has been added and cited appropriately within both the Methods and Discussion sections.

**Comment 3**

**No Statistical Significance Testing for Model Comparisons**

The comparative analysis rests entirely on point estimates. The three top models differ in accuracy by less than 0.005. At this margin, no superiority claim is statistically defensible without formal testing. McNemar test and Friedman test with Nemenyi post-hoc correction are recommended. All metrics in Table 1 should report mean and standard deviation across cross-validation folds rather than single point estimates.

**Response**

We thank the reviewer for this important suggestion.

The manuscript has been revised to provide a more statistically rigorous comparison of model performance.

Specifically, we now report cross-validation performance using **mean values together with standard deviations and 95% confidence intervals**, providing a better representation of model variability across folds rather than relying solely on point estimates.

Furthermore, we substantially revised the Results and Discussion to avoid implying superiority based on very small numerical differences. Instead, we emphasise that CatBoost, LightGBM, Random Forest, and Logistic Regression demonstrated comparable predictive performance, with model selection depending on deployment objectives such as interpretability, computational efficiency, calibration, and scalability.

This more conservative interpretation better reflects the observed performance differences and addresses the reviewer's concern regarding overinterpretation.

**Comment 4**

**SHAP Figure Uses Generic Feature Labels**

Fig 10 displays only "Feature 0," "Feature 1," through "Feature 10" instead of actual feature names. This indicates the SHAP analysis was run on a preprocessed numpy array where column names were lost after encoding or standardization. The figure directly contradicts the text claiming Chlorosis Intensity, Green Yellow Ratio, and Necrotic Area are the most influential predictors. The authors must use a named pandas DataFrame as input to the SHAP explainer and regenerate Fig 10 with correct feature labels.

**Response**

We appreciate the reviewer identifying this issue.

The SHAP analysis has been repeated using the original feature-labelled dataset to preserve feature names throughout the explainability pipeline.

Figure 10 has been regenerated and now displays the actual feature names rather than generic labels. The revised figure clearly identifies biologically meaningful predictors including **Chlorosis Intensity**, **Green–Yellow Ratio**, **Necrotic Area**, **Infected Distribution**, and related visual descriptors.

The accompanying Results and Discussion sections have also been revised to ensure complete consistency between the figures and the textual interpretation.

**Comment 5**

**Fig 11 Was Not Submitted**

The SHAP beeswarm plot is referenced and described in detail but was not included in the submission. It must be provided.

**Response**

We thank the reviewer for noting this omission.

The missing SHAP beeswarm figure has now been included in the revised manuscript.

The associated Results section has been revised to correspond directly with the submitted figure, and the figure caption has been expanded to improve interpretation.

**Comment 6**

**Partial Dependence Plot Text Does Not Match Fig 9**

Lines 326–328 state that Fig 9 illustrates the relationship between Greenness Intensity and predicted infection probability. The actual Fig 9 shows PDPs for Chlorosis Intensity, Green Yellow Ratio, and Necrotic Area. The feature "Greenness Intensity" does not exist in the dataset. The text must be corrected.

**Response**

We appreciate the reviewer identifying this inconsistency.

The text describing the partial dependence plots has been corrected to accurately reflect the features presented in Figure 9.

The revised manuscript now correctly discusses the partial dependence relationships for **Chlorosis Intensity**, **Green–Yellow Ratio**, and **Necrotic Area**, and removes all incorrect references to "Greenness Intensity."

**Comment 7**

**Contradiction Between PDP and SHAP for Green Yellow Ratio**

The SHAP description states that lower values of Green Yellow Ratio are associated with higher predicted risk, whereas the PDP shows a monotonically increasing relationship. These two results directly contradict each other. The authors must identify and resolve the source of this contradiction before publication.

**Response**

We thank the reviewer for this careful observation.

We revisited the explainability analysis and identified that the inconsistency arose from an incorrect interpretation of the SHAP outputs in the original manuscript rather than from the underlying model.

The SHAP analysis, PDP interpretation, and accompanying discussion have all been carefully re-evaluated and rewritten to ensure consistency across all explainability methods.

Furthermore, we substantially expanded the explainability section by incorporating:

- a revised SHAP global importance analysis;
- SHAP interaction analysis;
- SHAP waterfall explanations for representative healthy and infected leaves;
- improved LIME interpretations;
- and stronger biological interpretation linking the identified features to established MLND symptom progression reported in the plant pathology literature.

These revisions provide a consistent and biologically meaningful interpretation of feature effects across SHAP, PDP, and LIME analyses.

**Comment 8. No Ablation Study for Class Imbalance Handling**

*The claim that SMOTE and class weighting contributed to improved minority class detection requires a controlled comparison. No baseline results without imbalance handling are presented. Without this, the contribution of these techniques cannot be quantified. The authors should add baseline experiments for each model without imbalance handling and report the difference in recall and F1 for the minority class.*

**Response:**

Thank you for this valuable suggestion. We have incorporated a comprehensive ablation study comparing model performance with and without class imbalance mitigation. Specifically, we evaluated all classifiers under three settings: (i) no imbalance handling, (ii) class weighting only, and (iii) SMOTE combined with class weighting. The revised Results section now reports the corresponding changes in minority-class Recall, F1-score, Balanced Accuracy, and AUC. The findings demonstrate that class imbalance mitigation consistently improved minority-class detection, particularly for ensemble-based models, thereby providing quantitative evidence supporting our methodological choice.

**Comment 9. Hardware Specifications Absent from Scalability Analysis**

*Table 2 reports training time and inference latency without stating the CPU model, RAM, operating system, or library versions used.*

**Response:**

We appreciate this observation. A dedicated Computational Environment subsection has been added to the Methods section. The revised manuscript now reports the processor specifications, available RAM, operating system, Python version, and major software libraries used during model development and scalability experiments. This information improves reproducibility and enables fair comparison with future studies.

**Comment 10. Hyperparameter Values for Final Models Not Reported**

*Grid search with 5-fold cross-validation is described but the final selected hyperparameter values for all nine models are never reported.*

**Response:**

Thank you for highlighting this reproducibility issue. We have substantially expanded the **Hyperparameter Optimization** subsection by reporting the search ranges, optimization strategy, cross-validation procedure, random seed, stopping criteria where applicable, and the final optimized hyperparameter values for all evaluated models. In addition, a supplementary table summarizing the optimized hyperparameters has been included to facilitate reproducibility.

**Comment 11. Brier Score Misinterpreted**

*The authors attribute low Brier scores to better calibration. Calibration should be assessed using Expected Calibration Error (ECE) or the Hosmer-Lemeshow test.*

**Response:**

We appreciate this important clarification. The calibration analysis has been revised to distinguish between **overall probabilistic accuracy (Brier Score)** and **probability calibration**. In addition to calibration curves, we now report **Expected Calibration Error (ECE)**. The discussion has been revised accordingly to avoid overstating the interpretation of Brier scores and to provide a more rigorous assessment of model calibration.

**Comment 12. Comparison with Prior MLND Studies Lacks Specificity**

*The discussion should include direct numerical comparisons with previous MLND studies and explain why publicly available datasets were not used.*

**Response:**

Thank you for this suggestion. The Comparison with Existing Studies subsection has been substantially expanded. The revised manuscript now includes direct comparisons with recent MLND studies employing deep learning approaches, highlighting methodological differences between image-based deep learning and the structured feature-based machine learning framework adopted in this study. We also clarify that the present study utilizes a secondary dataset obtained from KALRO, which provides structured field-derived observations appropriate for evaluating interpretable machine learning models under realistic agricultural conditions.

**Comment 13. Limitations Section is Inadequate**

*The limitations section should address dataset characteristics, absence of external validation, lack of field deployment testing, practical implications of the reported accuracy, and institutional constraints.*

**Response:**

We thank the reviewer for this recommendation. The **Limitations** section has been substantially expanded. It now discusses the use of secondary field-derived data, the absence of independent external validation, the lack of real-world deployment experiments, the implications of the observed classification accuracy, and directions for future research, including multi-institutional validation and prospective field evaluation.

**Comment 14. Conclusion Overstates Findings**

*The conclusion describes a system with 0.753 accuracy as reliable and deployable. At this accuracy level approximately one in four samples is misclassified.*

**Response:**

We appreciate this observation. The Conclusion has been carefully revised to present a more balanced interpretation of the findings. Statements implying immediate deployment have been moderated, and the manuscript now emphasizes that the proposed framework represents a promising decision-support approach that requires further external validation before operational deployment. The discussion also explicitly acknowledges the trade-off between predictive performance, interpretability, and computational efficiency.

**Minor Comments**

**Minor Comment 1. Figure 15 LIME has truncated feature labels**

**Response:**

Thank you for identifying this formatting issue. The Figure has been regenerated at higher resolution with enlarged margins and complete feature labels to improve readability.

**Minor Comment 2. Figure 8 Learning Curve lacks x-axis label**

**Response:**

The learning curves results were removed.

**Minor Comment 3. Model size units are inconsistent**

**Response:**

Thank you for noting this inconsistency. Model sizes are now reported consistently in **megabytes (MB)** throughout the manuscript, including the scalability table and accompanying discussion.

**Minor Comment 4. Grammatical error**

*"The author declare no conflicts of interest."*

**Response:**

This grammatical error has been corrected to:

*"The author declares no conflicts of interest."*

**Minor Comment 5. Overuse of Reference [3]**

**Response:**

We appreciate this observation. The literature review has been revised by replacing several general citations with more specific primary references that directly support the corresponding methodological and application-related statements.

**Minor Comment 6. Inappropriate citation supporting SMOTE**

*Reference [21], a coronary heart disease prediction paper, is cited to support SMOTE in agricultural disease detection.*

**Response:**

Thank you for identifying this issue. The inappropriate citation has been replaced with the original foundational SMOTE paper by **Chawla et al. (2002)** together with additional domain-relevant references on imbalanced learning in agricultural machine learning.

**Response to Reviewer #2**

We sincerely thank the reviewer for the careful assessment of our manuscript and for the constructive comments. We appreciate the recognition of the relevance of the study to precision agriculture and food security. We have carefully revised the manuscript to address all concerns raised. Our point-by-point responses are provided below.

**Comment 1. Dataset description is unclear and inconsistent**

*The largest problem is the unclear nature of the dataset. The abstract and methods say the dataset is from real observations and from KALRO, but the introduction also says the study uses a "large-scale synthetic dataset designed to mimic real world MLND characteristics". This is a major contradiction. The paper must clearly state if the data are real, synthetic, simulated, or partly synthetic.*

**Response:**

We thank the reviewer for identifying this important inconsistency. The contradiction arose from an error in the original Introduction. The manuscript has been thoroughly revised to ensure consistency across all sections. We now clearly state that the study utilizes secondary field-derived data obtained from the Kenya Agricultural and Livestock Research Organization (KALRO) comprising 7,892 observations collected from major maize-growing counties in Kenya. All references describing the dataset as synthetic have been removed. In addition, the Study Design and Data Source subsection has been expanded to clearly describe the data source, study design, variables, and the nature of the structured feature dataset.

**Comment 2. The class imbalance is only moderate**

*The dataset has 5,400 healthy samples and 2,492 infected samples, with a ratio of about 2.17:1. This is only moderate imbalance. The title gives strong emphasis to class imbalance, but the paper does not show clearly how imbalance affects the models. There is no clear comparison between models trained with and without SMOTE or class weighting.*

**Response:**

We appreciate this observation. Although the imbalance ratio is moderate, minority-class misclassification remains a practical concern in disease detection because failing to identify infected plants has significant agricultural consequences. To address this concern, we have added an ablation study comparing classifier performance under three experimental settings: (i) no imbalance handling, (ii) class weighting only, and (iii) SMOTE combined with class weighting. The revised Results section now quantitatively demonstrates the impact of imbalance mitigation on Recall, F1-score, Balanced Accuracy, and AUC, thereby providing evidence of the contribution of these techniques.

**Comment 3. Methodology lacks sufficient implementation details**

*Many models are tested, including Logistic Regression, SVM, Random Forest, XGBoost, LightGBM, CatBoost, and MLP. But the paper does not give enough detail about feature extraction, parameter grids, SMOTE application, leakage control, or statistical testing. The paper says SMOTE and class weighting were used, but it does not clearly state whether SMOTE was applied only to the training data and inside each cross-validation fold.*

**Response:**

We thank the reviewer for this valuable recommendation. The Methods section has been substantially expanded to improve transparency and reproducibility. Specifically, we now provide:

- a clearer description of the structured feature dataset and variables;
- detailed information on the SMOTE implementation, including the variant used, oversampling parameters, and the application of SMOTE exclusively to the training data within each cross-validation fold to prevent data leakage;
- comprehensive descriptions of the hyperparameter search space, optimized hyperparameter values, random seed, software versions, and computational environment; and
- statistical significance analyses, including McNemar's test, DeLong's test, Wilcoxon signed-rank test, and Friedman/Nemenyi comparisons, together with confidence intervals for the reported performance metrics.

These additions substantially improve the reproducibility and methodological rigor of the study.

**Comment 4. The results are useful but over-interpreted**

*CatBoost has the best accuracy, while Logistic Regression has the best AUC and F1 score. This is interesting, but the authors describe many results as "strong" although the performance is moderate. An AUC around 0.80 and accuracy around 0.75 are promising, but not enough to claim reliable field deployment without external validation.*

**Response:**

We appreciate this thoughtful comment and agree that our original interpretation was overly optimistic. The Abstract, Discussion, and Conclusion have been carefully revised to provide a more balanced interpretation of the findings. Rather than describing the models as "reliable" or immediately suitable for deployment, we now characterize the reported performance as promising within the context of structured feature-based machine learning. We explicitly acknowledge that external validation and prospective field evaluation remain necessary before operational deployment. The Discussion also emphasizes the trade-offs between predictive performance, interpretability, calibration, and computational efficiency, particularly for resource-constrained agricultural settings.

**Response to Reviewer #3**

We sincerely thank the reviewer for the insightful and constructive comments. We appreciate the positive assessment of the importance of the research topic and the valuable suggestions for strengthening the manuscript. We have carefully revised the manuscript to address all major and minor concerns. Our detailed responses are provided below.

**Major Comments**

**Comment 1. Novelty should be better justified**

*The manuscript highlights novelty in three key aspects: (1) dataset imbalance, (2) comparative study with numerous ML models, and (3) utilization of large-scale synthetic data to mimic MLND characteristics. There is a need to address the following points which will surely highlight the novelty of the manuscript in a much clearer manner.*

**Firstly,** majority of real-life datasets such as medical diagnostics or agriculture are normally imbalanced. There is a need to include examples of publicly available balanced datasets existing in this research domain and studies with comparable performances.

**Secondly,** the manuscript states that rigorous comparison of ML algorithms was conducted "bridging the gap between classical and modern approaches." However, CatBoost achieved only 75.30% accuracy. The manuscript should clearly identify the factors contributing to the gap between classical and modern approaches. Moreover, comparing these machine learning models with recent deep learning approaches would enhance the scientific rigor.

**Thirdly,** there is a stronger need to elaborate the protocol followed for obtaining the "large-scale synthetic data" and clarify whether the dataset is publicly available or was generated as part of this study.

**Response:**

We sincerely thank the reviewer for these valuable observations. The Introduction has been substantially revised to better justify the novelty and positioning of the study.

First, we now discuss the prevalence of class imbalance in agricultural disease datasets while acknowledging that moderate imbalance is common in real-world applications. We also include recent literature to contextualize the problem and clarify the motivation for evaluating imbalance mitigation techniques.

Second, the comparison with existing studies has been significantly expanded. The revised Discussion now directly compares the performance of the evaluated machine learning models with recent deep learning approaches for MLND detection. We explain that the objective of this study was not to outperform deep learning methods in classification accuracy, but rather to evaluate the trade-offs between predictive performance, interpretability, computational efficiency, calibration, and deployment suitability using structured feature-based machine learning. This discussion clarifies the practical gap addressed between classical and modern machine learning approaches.

Third, we have corrected the dataset description throughout the manuscript. The dataset is not synthetic. All references to synthetic data have been removed, and the manuscript now consistently states that the study uses secondary field-derived data obtained from the Kenya Agricultural and Livestock Research Organization (KALRO). The Study Design and Data Source section has been expanded accordingly.

**Comment 2. The manuscript states that the study develops a structured and scalable dataset but does not explain the interpretable visual features.**

*There is a stronger need to provide detailed insights into the term "interpretable visual features", clearly elaborating the source of these visual features such as images or videos.*

**Response:**

Thank you for this important suggestion. The Study Design and Data Source and Variables and Feature Description sections have been expanded to provide a clearer description of the structured dataset and the engineered visual descriptors. We now explicitly state that the dataset consists of structured quantitative variables representing colour, texture, lesion morphology, and spatial characteristics of maize leaves obtained from secondary KALRO data. We further clarify that the study evaluates machine learning models using these structured descriptors rather than performing image processing or feature extraction within the present work.

**Comment 3. Resource-constrained environments should be better contextualized**

*Including a few examples of devices with specifications will establish the context properly.*

**Response:**

We appreciate this recommendation. The manuscript has been revised to better contextualize resource-constrained deployment scenarios by discussing lightweight computing platforms typically used in precision agriculture, including mobile devices, edge computing platforms, and single-board computers such as Raspberry Pi-class hardware. The scalability analysis now better explains how computational efficiency, memory usage, and inference latency influence practical deployment in such environments.

**Comment 4. The heading "Model Development" is misleading**

*The manuscript includes a heading "Model Development" whereas the study only includes comparative analysis of ML models.*

**Response:**

We thank the reviewer for this observation. The section has been renamed "Machine Learning Model Implementation" to more accurately reflect the scope of the study. The revised section now explains the rationale for selecting the different machine learning algorithms and clarifies that the objective was a comparative evaluation using a common structured feature representation rather than proposing a novel machine learning model.

**Comment 5. Hyperparameters should be fully reported**

*Hyperparameters for MLP, RF, XGBoost, and SVM need to be elaborated together with empirical reasons for the selected values.*

**Response:**

Thank you for this valuable suggestion. The hyperparameter optimization section has been substantially expanded. The revised manuscript now reports the parameter search ranges, optimization strategy, final optimized hyperparameter values, random seed, stopping criteria where applicable, and the rationale for selecting these parameters.

**Comment 6. Recommend one best model for resource-constrained settings**

*Considering recall, F1-score, training time, and model size, suggesting one best-fit model with logical reasoning for resource-constrained settings would be extremely valuable.*

**Response:**

We appreciate this excellent suggestion. The Abstract, Discussion, and Scalability Analysis have been revised accordingly. While CatBoost achieved the highest classification accuracy, the revised manuscript now concludes that LightGBM provides the best overall balance between predictive performance, computational efficiency, model size, and inference speed, making it the most suitable candidate for deployment in resource-constrained agricultural environments. This recommendation is supported by the combined evaluation of predictive accuracy, calibration, scalability, and computational cost rather than classification performance alone.

**Minor Comments**

**Comment 1. Line #85: "algorithms was implemented" should be corrected grammatically.**

**Response:**

Thank you for identifying this error. The grammatical mistake has been corrected throughout the manuscript.

**Comment 2. Mathematical definitions of CatBoost and LightGBM should be added.**

**Response:**

We appreciate this recommendation. Mathematical formulations and accompanying descriptions for both CatBoost and LightGBM have been added to the Machine Learning Model Implementation section to improve methodological completeness and enhance reader understanding of the best-performing models.

**Comment 3. Include collective inference time for the entire test set**

*Including inference time for the whole test set would provide additional insights.*

**Response:**

We thank the reviewer for this useful suggestion. In addition to reporting per-sample inference latency, the scalability analysis has been expanded to discuss the computational implications for batch prediction and practical deployment scenarios. This provides a more comprehensive evaluation of model efficiency during large-scale inference.

**Comment 4. Explain why these specific ML models were selected**

*Highlighting the reasons for choosing these specific ML models will enhance understanding.*

**Response:**

Thank you for this suggestion. The Machine Learning Model Implementation section has been revised to explain the rationale for selecting each class of algorithms. The evaluated models were chosen to represent diverse machine learning paradigms—including linear, kernel-based, tree-based, ensemble, and neural network methods—allowing a comprehensive comparison of predictive performance, interpretability, computational efficiency, and robustness using a common structured feature representation.

**Comment 5. Resolution of figures should be improved.**

**Response:**

We appreciate this recommendation. All figures have been regenerated at higher resolution to improve readability and comply with the journal's figure quality requirements.

**Comment 6. Some figures are cropped from the left side.**

**Response:**

Thank you for identifying this formatting issue. The affected figures have been resized and reformatted to ensure that all labels, axes, and annotations are fully visible in the revised manuscript.

We sincerely thank Reviewer #3 for the constructive comments and recommendations. Addressing these suggestions has substantially strengthened the manuscript by improving its methodological clarity, novelty justification, reproducibility, interpretability, and practical relevance.

**Response to Reviewer #4**

We sincerely thank the reviewer for the thorough and constructive assessment of our manuscript. We appreciate the recognition of the relevance of the research problem and the positive comments regarding the comprehensive evaluation of machine learning models, explainable AI, calibration analysis, and computational efficiency. We have carefully revised the manuscript to address all concerns raised. Our detailed responses are provided below.

**Major Comments**

**Comment 1. Dataset description is inconsistent and unclear**

*The manuscript states that the dataset consists of 7,892 observations collected from KALRO, while the Introduction later states that the study utilizes a large-scale synthetic dataset designed to mimic real-world MLND characteristics. These two descriptions contradict each other and create confusion regarding the actual data source. The authors should clearly explain whether the dataset is entirely real, entirely synthetic, or partially synthetic. The manuscript should describe exactly how the data were collected or generated.*

**Response:**

We thank the reviewer for identifying this important inconsistency. The contradiction resulted from an inadvertent statement retained in the Introduction. The manuscript has been comprehensively revised to ensure consistency across all sections. We now clearly state that the study uses secondary field-derived data obtained from the Kenya Agricultural and Livestock Research Organization (KALRO) comprising 7,892 observations collected from major maize-growing counties in Kenya. All references describing the dataset as synthetic have been removed. In addition, the Study Design and Data Source section has been substantially expanded to describe the study design, data source, variables, and characteristics of the structured feature dataset.

**Comment 2. Lack of detailed feature extraction methodology**

*The manuscript refers to features such as Chlorosis Intensity, Green-Yellow Ratio, Necrotic Area, Lesion Distribution, and Texture Roughness but never explains how these variables were computed. The paper should specify image acquisition, segmentation, preprocessing, mathematical definitions, software used, and whether feature extraction was manual or automated.*

**Response:**

We appreciate the reviewer's concern. The present study utilizes secondary structured data obtained from KALRO, and the feature extraction process was performed prior to data acquisition. Consequently, the original image acquisition, segmentation pipeline, preprocessing procedures, and feature engineering algorithms are beyond the scope of this study and were not available to the authors. To avoid any ambiguity, the manuscript now explicitly states that the study evaluates machine learning models using pre-extracted structured visual, texture, lesion, and morphological descriptors provided within the secondary dataset rather than performing feature extraction from raw images. The Study Design and Data Source and Variables and Feature Description sections have been revised accordingly to clearly describe the available variables and their role in model development.

**Comment 3. Insufficient information about class imbalance handling**

*The authors mention SMOTE and class weighting but omit critical implementation details.*

**Response:**

Thank you for this important observation. The Methods section has been substantially expanded to provide complete implementation details. The revised manuscript now specifies the SMOTE variant employed, oversampling parameters, nearest-neighbor settings, application of SMOTE exclusively to the training data within each cross-validation fold to prevent data leakage, and the complete cross-validation workflow. These additions substantially improve methodological transparency and reproducibility.

**Comment 4. Missing external validation**

*All results appear to be obtained from a single dataset using an 80/20 split. There is no validation using an external dataset, independent field data, or temporal validation.*

**Response:**

We appreciate this valuable suggestion. To strengthen the assessment of model generalizability, we incorporated **Leave-One-County-Out Cross-Validation (LOCO-CV)**, whereby models were trained using data from all counties except one and subsequently evaluated on the held-out county. This geographic validation provides a more rigorous assessment of spatial generalizability across maize-growing regions in Kenya. We have also explicitly acknowledged the absence of an independent external dataset as a limitation and recommend external validation using datasets collected from different geographical regions and growing seasons in future work.

**Comment 5. Statistical significance is missing**

*Although many algorithms are compared, no statistical tests are reported.*

**Response:**

We thank the reviewer for this important recommendation. The statistical analysis has been substantially strengthened. The revised manuscript now includes McNemar's test, DeLong's test for AUC comparisons, Wilcoxon signed-rank tests, and Friedman tests with Nemenyi post-hoc comparisons where appropriate. In addition, 95% confidence intervals are reported for the principal performance metrics. These additions provide statistical evidence supporting the comparative conclusions presented in the manuscript.

**Comment 6. Hyperparameter tuning lacks reproducibility**

*The manuscript only mentions Grid Search.*

**Response:**

We appreciate this recommendation. The hyperparameter optimization procedure has been comprehensively expanded to include the search ranges, optimization strategy, final optimized hyperparameter values, random seed, stopping criteria, software versions, and computational environment. Furthermore, a supplementary table containing the optimized hyperparameters for all evaluated models has been included to improve reproducibility.

**Comment 7. Explainability analysis is largely descriptive**

*The SHAP and LIME analyses simply identify important variables. The discussion should better connect these findings to plant pathology, MLND symptoms, biological interpretation, and previous agronomic studies.*

**Response:**

We thank the reviewer for this valuable suggestion. The explainability section has been substantially revised to provide deeper biological interpretation of the SHAP, LIME, Partial Dependence Plot, and SHAP interaction analyses. The revised discussion now explicitly relates the identified predictors—including chlorosis intensity, green-yellow ratio, necrotic area, lesion distribution, and texture characteristics—to the known progression and symptomatology of MLND, supported by recent plant pathology and agricultural literature. These revisions considerably strengthen the scientific interpretation of the explainability results.

**Comment 8. Novelty should be better justified**

*The manuscript claims novelty through comparison of ML algorithms, class imbalance, and explainable AI. However, many recent papers already compare classical ML algorithms and apply SHAP/LIME. The authors should clearly explain what methodological innovation this work introduces.*

**Response:**

We appreciate this important observation. The Introduction has been extensively revised to better articulate the contribution of the study. The manuscript now clarifies that the novelty lies not in the individual machine learning algorithms or explainability techniques, but in their integrated application within a unified evaluation framework for MLND detection. Specifically, the revised manuscript highlights the comprehensive evaluation of diverse machine learning paradigms using structured field-derived features, incorporation of class imbalance mitigation, calibration analysis, complementary explainability methods, geographic validation through LOCO-CV, and scalability assessment within a single reproducible framework. This clarification better positions the study relative to existing literature.

**Minor Comments**

**Comment 1. Several grammatical issues remain throughout the manuscript.**

**Response:**

Thank you for this recommendation. The entire manuscript has undergone comprehensive language editing to improve grammar, consistency, and readability. Hyphenation and terminology (e.g., *feature-based*, *real-world*, *Multi-Layer Perceptron*) have been standardized throughout.

**Comment 2. Figure captions should be more informative.**

**Response:**

We appreciate this suggestion. All figure captions have been revised to provide additional methodological context, including descriptions of the displayed analyses, principal observations, and interpretation of the results where appropriate.

**Comment 3. The computational environment should be reported.**

**Response:**

Thank you for this recommendation. A dedicated Computational Environment subsection has been added to the Methods section, reporting the processor, memory specifications, operating system, Python version, and software library versions used for model development and evaluation.

**Comment 4. Report confidence intervals.**

**Response:**

I appreciate this suggestion. The revised manuscript now reports 95% confidence intervals for the principal performance metrics, including Accuracy, AUC, Precision, Recall, and F1-score, providing a more rigorous assessment of model uncertainty.

**Comment 5. The Discussion repeats the Results.**

**Response:**

Thank you for this constructive observation. The Discussion has been substantially revised to focus on critical interpretation rather than repetition of numerical results. The revised section now emphasizes the practical implications of the findings, biological interpretation of the explainability analyses, comparison with recent literature, deployment considerations, and study limitations.

**Comment 6. Source code and trained models should be shared.**

**Response:** We appreciate the reviewer's recommendation. To further improve the reproducibility and transparency of this study, the complete implementation has been made publicly available. The source code, preprocessing scripts, statistical analysis scripts, explainable AI (SHAP, LIME, and PDP) implementations, and trained machine learning models have been deposited in a publicly accessible Zenodo repository, available at [**https://doi.org/10.5281/zenodo.21473059**](https://doi.org/10.5281/zenodo.21473059).

**Closing Statement**

I sincerely thank the Academic Editor and all four reviewers for their thorough, thoughtful, and constructive comments. Their feedback has substantially strengthened the manuscript by improving its methodological rigor, reproducibility, statistical analysis, scientific interpretation, clarity, and practical relevance.

In response to the reviewers' recommendations, we have comprehensively revised the manuscript by clarifying the data source, expanding the methodological descriptions, strengthening the statistical analyses, improving the explainability and discussion sections, enhancing the novelty statement, incorporating geographic validation, reporting confidence intervals and optimized hyperparameters, expanding the scalability analysis, and improving the overall presentation of the manuscript.

I believe that these extensive revisions have addressed the reviewers' concerns and have significantly improved the quality and scientific contribution of the manuscript. I am grateful for the opportunity to revise my work and respectfully submit the revised manuscript for your consideration.
