# Supplementary material for: Comparative evaluation of machine learning algorithms for Maize Lethal Necrosis Disease detection under class imbalance
Source: PLoS One. 2026 Oct 23;21(10):e0349738. doi: 10.1371/journal.pone.0349738 (PMC13693939; doi:10.1371/journal.pone.0349738)
Supplement: Supplementary file 7 [file pone.0349738.s007.docx]

**Comments on PONE-D-26-21835_R1**

The revised manuscript has been improved considerably. However, there are still few concerns:

**Major Concerns**

1. The manuscript mentions “resource-constrained environments” in various sections. Although the detailed specifications of hardware used for the experimental study is included. Still, adding a few examples of platforms suitable for real-time operations will establish the context properly.
2. The manuscript mentions “secondary dataset” multiple times in the entire manuscript. (line # 100, 106, 344 etc). However, the repository records (<https://zenodo.org/records/19997730>) again create certain confusions about the data source. As the entire study is based on it, so the authors should provide more details on it.

**Minor Concerns**

1. The same abbreviations such as MLND (Line # 99, Line # 4, Line # 358) has been defined multiple times.
2. Providing access link of the repository

Rest of the manuscript has been improved. My recommendation is a minor revision at the moment before moving forward.
