# Supplementary material for: Comparative evaluation of machine learning algorithms for Maize Lethal Necrosis Disease detection under class imbalance
Source: PLoS One. 2026 Oct 23;21(10):e0349738. doi: 10.1371/journal.pone.0349738 (PMC13693939; doi:10.1371/journal.pone.0349738)
Supplement: Supplementary file 8 [file pone.0349738.s008.docx]

**Response to Reviewers**

**Manuscript ID:** PONE-D-26-21835R1

**Title:** Comparative Evaluation of Machine Learning Algorithms for Maize Lethal Necrosis Disease Detection Under Class Imbalance

Dear Dr. Okagbue and Reviewers,

We thank the Academic Editor and the reviewers for their thoughtful and constructive comments on my manuscript. I am grateful for the detailed feedback, which has helped me substantially improve the quality of my work. I have carefully addressed all remaining concerns raised by the reviewers. Below, I provide a point-by-point response to each comment.

**Reviewer #1**

I thank Reviewer #1 for the thorough and encouraging evaluation of our revised manuscript. I am pleased that the reviewer acknowledged the substantial improvements made in response to the first round of reviews. I have carefully addressed the two remaining issues and the moderate concern as detailed below.

**Issue 1: SMOTE-ENN Reference in the Results Section**

**Reviewer's Comment:** The methods section correctly states that no SMOTE-ENN hybrid resampling was performed. However, the results section still contains the sentence: *"These findings support the use of imbalance handling techniques such as SMOTE-ENN and provide a strong foundation for subsequent comparative machine learning analyses."* This contradicts the methods statement and must be corrected.

**Author's Response:** I thank the reviewer for identifying this inconsistency. I have corrected the sentence in the Exploratory Data Analysis subsection of the Results section. The phrase "SMOTE-ENN" has been replaced with "SMOTE," which is the technique actually used in this study.

**Issue 2: Data Availability Statement**

**Reviewer's Comment:** The Data Availability Statement still points to https://doi.org/10.5281/zenodo.19997730, which the reviewer identified as non-resolving. The new DOI for code and models (https://doi.org/10.5281/zenodo.21473059) appears only in the response letter and not in the manuscript. Both DOIs must be listed explicitly if the data and code are in different locations.

**Author's Response:** I thank the reviewer for this important clarification. Upon re-verification, I confirm that the dataset DOI (https://doi.org/10.5281/zenodo.19997730) is active and correctly points to the dataset used in this study. I have updated the Data Availability Statement to explicitly list both the dataset DOI and the separate DOI for the source code and trained models. The statement now clearly informs readers where to access both the data and the code.

**Revised Text in the Manuscript**

"The dataset used in this study is publicly available in the Zenodo repository at \url{[https://doi.org/10.5281/zenodo.19997730}](https://doi.org/10.5281/zenodo.19997730%7D). The source code and trained machine-learning models used for the analysis are also publicly available in a separate Zenodo repository at \url{[https://doi.org/10.5281/zenodo.21473059}](https://doi.org/10.5281/zenodo.21473059%7D)."

**Moderate Concern: Feature Extraction Methodology**

**Reviewer's Comment:** The manuscript does not explain how the features (Chlorosis Intensity, Green-Yellow Ratio, Necrotic Area) were computed from images. While the authors state that feature extraction was performed by KALRO prior to data acquisition and is beyond the scope of this study, one sentence acknowledging this limitation would complete the transparency of the manuscript.

**Author's Response:** I thank the reviewer for this important suggestion. I have added a new point to the Limitations section acknowledging that the feature extraction procedure was performed by KALRO prior to data acquisition and is not publicly available. I explicitly note that researchers seeking to apply this framework to new image collections would need to either obtain KALRO's feature extraction pipeline or develop their own equivalent feature engineering workflow.

**Reviewer #3**

I thank Reviewer #3 for acknowledging the improvements made to the manuscript. I have carefully addressed all remaining concerns as detailed below.

**Major Concern 1: Resource-Constrained Environments**

**Reviewer's Comment:** The manuscript mentions "resource-constrained environments" in various sections. Although the detailed specifications of hardware used for the experimental study are included, adding a few examples of platforms suitable for real-time operations will establish the context properly.

**Author's Response:** I thank the reviewer for this valuable suggestion. I have added two sentences to the "Scalability and Practical Implications" subsection of the Discussion to provide specific recommendations for real-world deployment platforms. Based on the scalability analysis, I now suggest that lightweight models such as Logistic Regression and LightGBM are suitable for mid-range smartphones and Raspberry Pi 4 devices, while CatBoost and Random Forest may be better suited for cloud-based virtual machines or edge servers with moderate computational resources.

**Major Concern 2: Data Source Confusion**

**Reviewer's Comment:** The manuscript mentions "secondary dataset" multiple times. However, the repository records (https://zenodo.org/records/19997730) create certain confusions about the data source. As the entire study is based on it, the authors should provide more details on it.

**Author's Response:** I thank the reviewer for this important clarification. I have substantially expanded the "Study Design and Data Source" subsection in the Materials and Methods to provide comprehensive details about the dataset's origin. I now explicitly describe: (1) that the original images were acquired from farmers' fields across multiple maize-growing counties in Kenya under varying field conditions, (2) that KALRO plant pathologists visually confirmed MLND infection status based on established symptom criteria, (3) that the structured features were extracted from the images using KALRO's proprietary image analysis pipeline, (4) that the resulting dataset was provided to the author in an anonymized, tabular format containing only the engineered features and associated labels, with no raw images or identifiable farmer information included, and (5) that this secondary dataset formed the basis for all analyses in the present study. I also clarify that the study focuses on comparative algorithm evaluation using these provided features, not on developing new feature extraction methods.

**Minor Concern 1: Duplicate MLND Definitions**

**Reviewer's Comment:** The same abbreviations such as MLND (Line # 99, Line # 4, Line # 358) have been defined multiple times.

**Author's Response:** I thank the reviewer for this suggestion. I have verified that "Maize Lethal Necrosis Disease (MLND)" is defined once in the Abstract and once at its first mention in the Introduction. All subsequent occurrences throughout the manuscript use only the abbreviation "MLND." No duplicate full definitions remain.

**Minor Concern 2: Repository Access Link**

**Reviewer's Comment:** Provide an access link to the repository.

**Author's Response:** I thank the reviewer for this suggestion. I have ensured that the repository links are prominently and correctly provided in the manuscript. A dedicated Data Availability Statement has been added after the Conclusion section, which includes the dataset DOI (https://doi.org/10.5281/zenodo.19997730) and the separate DOI for the source code and trained models (https://doi.org/10.5281/zenodo.21473059). Both links have been verified and are active.

I believe the revised manuscript now fully addresses all remaining concerns raised by the reviewers. I am grateful for the time and effort the reviewers and editors have invested in improving my work. I look forward to your favorable decision.

Yours sincerely,

Maurice Wanyonyi
Corresponding Author
University of Embu, Kenya
Email: iamwanyonyi93@gmail.com
