# Supplementary material for: Comparative evaluation of machine learning algorithms for Maize Lethal Necrosis Disease detection under class imbalance
Source: PLoS One. 2026 Oct 23;21(10):e0349738. doi: 10.1371/journal.pone.0349738 (PMC13693939; doi:10.1371/journal.pone.0349738)
Supplement: Peer review history

# Peer review: Decision Letter 0

**DOI:** 10.1371/journal.pone.0349738.r001

**Authors:** Hilary Izuchukwu Okagbue

17 Jul 2026

Dear Dr. Wanyonyi,

Thank you for submitting your manuscript to PLOS ONE. After careful consideration, we feel that it has merit but does not fully meet PLOS ONE’s publication criteria as it currently stands. Therefore, we invite you to submit a revised version of the manuscript that addresses the points raised during the review process.

==============================

**ACADEMIC EDITOR: Major revision is recommended. Kindly ensure that all the comments area addressed satisfactorily.**

==============================

Please submit your revised manuscript by Sep 15 2026 11:59PM. If you will need more time than this to complete your revisions, please reply to this message or contact the journal office at plosone@plos.org. When you're ready to submit your revision, log on to https://www.editorialmanager.com/pone/ and select the 'Submissions Needing Revision' folder to locate your manuscript file.

- A letter that responds to each point raised by the academic editor and reviewer(s). You should upload this letter as a separate file labeled 'Response to Reviewers'.
- A marked-up copy of your manuscript that highlights changes made to the original version. You should upload this as a separate file labeled 'Revised Manuscript with Track Changes'.
- An unmarked version of your revised paper without tracked changes. You should upload this as a separate file labeled 'Manuscript'.

If you would like to make changes to your financial disclosure, please include your updated statement in your cover letter. Guidelines for resubmitting your figure files are available below the reviewer comments at the end of this letter.

If applicable, we recommend that you deposit your laboratory protocols in protocols.io to enhance the reproducibility of your results. Protocols.io assigns your protocol its own identifier (DOI) so that it can be cited independently in the future. For instructions see: https://journals.plos.org/plosone/s/submission-guidelines#loc-laboratory-protocols. Additionally, PLOS ONE offers an option for publishing peer-reviewed Lab Protocol articles, which describe protocols hosted on protocols.io. Read more information on sharing protocols at https://plos.org/protocols?utm_medium=editorial-email&utm_source=authorletters&utm_campaign=protocols.

As the corresponding author, your ORCID iD is verified in the submission system and will appear in the published article. PLOS supports the use of ORCID, and we encourage all coauthors to register for an ORCID iD and use it as well. Please encourage your coauthors to verify their ORCID iD within the submission system before final acceptance, as unverified ORCID iDs will not appear in the published article. *Only*  the individual author can complete the verification step; PLOS staff *cannot*  verify ORCID iDs on behalf of authors.

We look forward to receiving your revised manuscript.

Kind regards,

Academic Editor

PLOS One

Journal Requirements:

When submitting your revision, we need you to address these additional requirements.

1. Please ensure that your manuscript meets PLOS ONE's style requirements, including those for file naming. The PLOS ONE style templates can be found at

https://journals.plos.org/plosone/s/file?id=wjVg/PLOSOne_formatting_sample_main_body.pdf and

https://journals.plos.org/plosone/s/file?id=ba62/PLOSOne_formatting_sample_title_authors_affiliations.pdf

2. Please note that PLOS One has specific guidelines on code sharing for submissions in which author-generated code underpins the findings in the manuscript. In these cases, we expect all author-generated code to be made available without restrictions upon publication of the work. Please review our guidelines at https://journals.plos.org/plosone/s/materials-and-software-sharing#loc-sharing-code and ensure that your code is shared in a way that follows best practice and facilitates reproducibility and reuse.

3. PLOS requires an ORCID iD for the corresponding author in Editorial Manager on papers submitted after December 6th, 2016. Please ensure that you have an ORCID iD and that it is validated in Editorial Manager. To do this, go to ‘Update my Information’ (in the upper left-hand corner of the main menu), and click on the Fetch/Validate link next to the ORCID field. This will take you to the ORCID site and allow you to create a new iD or authenticate a pre-existing iD in Editorial Manager.

4. Please upload a copy of Figure 11, to which you refer in your text on page 12. If the figure is no longer to be included as part of the submission please remove all reference to it within the text.

5. If the reviewer comments include a recommendation to cite specific previously published works, please review and evaluate these publications to determine whether they are relevant and should be cited. There is no requirement to cite these works unless the editor has indicated otherwise.

[Note: HTML markup is below. Please do not edit.]

Reviewers' comments:

Reviewer's Responses to Questions

**Comments to the Author**

1. Is the manuscript technically sound, and do the data support the conclusions?

Reviewer #1: Partly

Reviewer #2: Partly

Reviewer #3: Partly

Reviewer #4: Yes

**********

2. Has the statistical analysis been performed appropriately and rigorously?

Reviewer #1: No

Reviewer #2: No

Reviewer #3: No

Reviewer #4: No

**********

3. Have the authors made all data underlying the findings in their manuscript fully available?

The PLOS Data policy

Reviewer #1: No

Reviewer #2: Yes

Reviewer #3: Yes

Reviewer #4: No

**********

4. Is the manuscript presented in an intelligible fashion and written in standard English?

Reviewer #1: No

Reviewer #2: Yes

Reviewer #3: Yes

Reviewer #4: Yes

**********

Reviewer #1: COMPREHENSIVE PEER REVIEW

Manuscript: PONE-D-26-21835

Title: Comparative Evaluation of Machine Learning Algorithms for Maize Lethal Necrosis Disease Detection Under Class Imbalance

OVERVIEW

This manuscript addresses a genuinely important problem. Maize Lethal Necrosis Disease remains a serious threat to food security across East Africa, and developing lightweight, interpretable detection tools for resource-limited settings is a worthwhile research direction. The study evaluates nine machine learning algorithms on a structured feature-based dataset, incorporates class imbalance handling, and applies explainable AI techniques. The research direction is sound. However, the manuscript has several critical technical, methodological, and presentation problems that must be resolved before it can be considered for publication.

CRITICAL ISSUES REQUIRING MANDATORY REVISION

1. Dataset Origin is Inconsistent and Unverifiable

Three different descriptions of the dataset appear across three sections: the abstract describes it as "derived from real observations," the introduction calls it "a large-scale synthetic dataset designed to mimic real-world MLND characteristics," and the methods describe it as "secondary data obtained from KALRO." These are mutually exclusive descriptions. The authors must provide one clear, consistent statement of the dataset origin across all sections.

The Zenodo DOI (https://doi.org/10.5281/zenodo.19997730) does not resolve to any accessible record, directly violating PLOS One data availability policy. If the data was obtained from KALRO under a formal data use agreement, any sharing restrictions must be explicitly stated. If the dataset is synthetic, it should be freely shareable and the DOI must be corrected before re-submission.

2. SMOTE vs. SMOTE-ENN Inconsistency

The methods section describes SMOTE as the oversampling technique. The results section references SMOTE-ENN without prior definition. These are different methods. SMOTE-ENN combines synthetic minority oversampling with Edited Nearest Neighbours undersampling, which removes borderline and noisy majority class samples near decision boundaries. This cleaning step can substantially alter the training data distribution compared to SMOTE alone. If SMOTE-ENN was used, the methods section must describe it fully including the ENN component, the number of neighbours used, and the rationale for choosing the combined approach. The foundational SMOTE paper (Chawla et al., 2002, Journal of Artificial Intelligence Research, 16:321-357) is absent from the reference list despite SMOTE being a core methodological component. This citation is mandatory.

3. No Statistical Significance Testing for Model Comparisons

The comparative analysis rests entirely on point estimates. The three top models differ in accuracy by less than 0.005: CatBoost (0.7530), LightGBM (0.7505), and Random Forest (0.7492). At this margin, no superiority claim is statistically defensible without formal testing. For pairwise classifier comparison on the same test set, McNemar test on the contingency table of correct and incorrect predictions is appropriate (Dietterich, 1998, Neural Computation, 10:1895-1923). For multiple classifier comparison across folds, the Friedman test with Nemenyi post-hoc correction is recommended (Demsar, 2006, Journal of Machine Learning Research, 7:1-30). Neither was applied. All metrics in Table 1 should report mean and standard deviation across cross-validation folds rather than single point estimates.

4. SHAP Figure Uses Generic Feature Labels

Fig 10 displays only "Feature 0," "Feature 1," through "Feature 10" instead of actual feature names. This indicates the SHAP analysis was run on a preprocessed numpy array where column names were lost after encoding or standardization. The figure directly contradicts the text claiming Chlorosis Intensity, Green Yellow Ratio, and Necrotic Area are the most influential predictors. The authors must use a named pandas DataFrame as input to the SHAP explainer and regenerate Fig 10 with correct feature labels. Without this correction the XAI conclusions cannot be verified.

5. Fig 11 Was Not Submitted

The SHAP beeswarm plot is referenced and described in detail at lines 344-348 with specific claims about how individual features influence predictions. This figure was not included in the submission. It must be provided.

6. Partial Dependence Plot Text Does Not Match Fig 9

Lines 326-328 state that Fig 9 illustrates the relationship between Greenness Intensity and predicted infection probability. The actual Fig 9 shows PDPs for Chlorosis Intensity, Green Yellow Ratio, and Necrotic Area. The feature "Greenness Intensity" does not exist in the dataset. The text must be corrected to accurately describe what Fig 9 shows.

7. Contradiction Between PDP and SHAP for Green Yellow Ratio

The SHAP description states that lower values of Green Yellow Ratio are associated with higher predicted risk, which is biologically consistent with MLND symptom progression where reduced healthy green tissue relative to chlorotic regions indicates infection. However, the PDP for Green Yellow Ratio in Fig 9 shows a monotonically increasing relationship, meaning higher values associate with higher predicted risk. These two results directly contradict each other. The authors must identify and resolve the source of this contradiction before publication.

8. No Ablation Study for Class Imbalance Handling

The claim that SMOTE and class weighting contributed to improved minority class detection requires a controlled comparison. No baseline results without imbalance handling are presented. Without this, the contribution of these techniques cannot be quantified. The authors should add baseline experiments for each model without imbalance handling and report the difference in recall and F1 for the minority class. This is especially important given that the imbalance ratio of 2.17:1 is mild. The imbalanced learning literature generally considers ratios below 4:1 as low imbalance (He and Garcia, 2009, IEEE Transactions on Knowledge and Data Engineering, 21:1263-1284), and aggressive resampling may not be necessary at this level.

9. Hardware Specifications Absent from Scalability Analysis

Table 2 reports training time and inference latency without stating the CPU model, RAM, operating system, or library versions used. These numbers are uninterpretable without hardware context and cannot be compared against other studies or used to support deployment recommendations. Hardware specifications must be added to the methods section.

10. Hyperparameter Values for Final Models Not Reported

Grid search with 5-fold cross-validation is described but the final selected hyperparameter values for all nine models are never reported. For reproducibility, these must be provided either in the methods section or as a supplementary table.

MODERATE ISSUES REQUIRING REVISION

11. Brier Score Misinterpreted

The authors attribute low Brier scores to better calibration. The Brier score measures overall probabilistic accuracy combining both discrimination and calibration. A model with strong discrimination but poor calibration can still achieve a low Brier score. Calibration should be assessed using Expected Calibration Error or the Hosmer-Lemeshow test. The calibration curves in Fig 7 show considerable deviation from the diagonal for all models. The authors should compute ECE for each model and clearly distinguish between discrimination and calibration in the discussion.

12. Comparison with Prior MLND Studies Lacks Specificity

References [16] and [17] are directly relevant MLND detection papers reporting specific performance metrics. The discussion should include direct numerical comparisons with these studies. Additionally, publicly available MLND datasets described in references [19] and [20] were not used. The authors should explain why a new dataset was generated rather than using these existing resources.

13. Limitations Section is Inadequate

Three sentences covering five lines is insufficient. The limitations section must address: the synthetic versus real data ambiguity; absence of external validation on an independent dataset; lack of field deployment testing; the practical implications of a 0.753 accuracy ceiling on a binary task; and single-author single-institution constraints on independent data validation.

14. Conclusion Overstates Findings

The conclusion describes a system with 0.753 accuracy as reliable and deployable. At this accuracy level, approximately 1 in 4 samples is misclassified. In agricultural disease detection this error rate has direct consequences for farmers. The authors should quantify the practical implications of this error rate rather than using unqualified terms. The word robust at line 481 is also inappropriate given that no external validation was performed.

MINOR ISSUES

Fig 15 LIME has truncated feature labels showing partial text such as "ea > 0.78" and "ss > 0.77." The figure must be regenerated with sufficient margin to display complete feature names.

Fig 8 learning curve x-axis shows training set size but carries no axis label. All axes must be fully labeled.

Table 2 reports model size in bytes while the text uses megabytes. Report in MB throughout for consistency.

Line 158-159 contains a grammatical error: "The author declare no conflicts of interest" should read "The author declares no conflicts of interest."

Reference [3] (Ngugi et al., 2024) is cited seven times across the manuscript to support claims ranging from class imbalance to SMOTE applicability to linear model calibration. Most of these require specific primary sources rather than a single broad crop disease review.

Reference [21], a coronary heart disease prediction paper by the corresponding author, is cited to support SMOTE in agricultural disease detection. A domain-appropriate primary citation should replace this reference.

CONSTRUCTIVE GUIDANCE FOR STRENGTHENING THIS WORK

On the dataset: Clearly state whether it is real, synthetic, or synthetic-derived-from-real field data and maintain this consistently across all sections. If synthetic, describe the generation process in sufficient detail for reproduction, including the statistical distributions used, the correlation structure between features, and how MLND symptom profiles were operationalized numerically.

On statistical analysis: Report cross-validated performance with standard deviations, apply McNemar test for pairwise comparisons, and use the Friedman test with Nemenyi post-hoc correction for overall model ranking. This makes all comparative claims statistically defensible.

On XAI: Perform all SHAP computations on a named pandas DataFrame to preserve feature names throughout the pipeline. Reporting SHAP interaction values for the top three features would add meaningful analytical depth beyond standard importance bar plots.

On calibration: Apply Platt scaling or isotonic regression post-hoc calibration to poorly calibrated models, then re-evaluate using ECE. This strengthens practical utility claims for threshold-based decision support tools intended for field use.

On scalability: Test at least one model on a Raspberry Pi 4 or equivalent low-cost single-board computer that approximates field deployment constraints in sub-Saharan Africa. This converts speculative deployment claims into evidence-based findings.

On feature engineering: Examine whether the high inter-correlations visible in Fig 2 indicate multicollinearity affecting model stability, particularly for Logistic Regression. Variance Inflation Factor analysis should be reported for the linear model.

SUMMARY

Major revision required. The dataset origin contradiction and broken Zenodo DOI are the most critical issues. Until these are resolved with a clear and verifiable data availability statement, the reproducibility of all reported results remains uncertain. The missing figure, mislabeled SHAP plot, PDP text mismatch, Green Yellow Ratio contradiction between PDP and SHAP, and absence of statistical significance testing are additional mandatory corrections. The research problem is important and practically relevant. With rigorous revision addressing the issues identified above, this work has the potential to make a meaningful contribution to the agricultural machine learning literature.

Reviewer #2: This paper studies machine learning models for detecting Maize Lethal Necrosis Disease using structured visual and texture features. The topic is relevant. Maize disease detection is important for food security, especially in Kenya and sub-Saharan Africa. The paper also discusses class imbalance, explainable AI, and scalability. These are useful topics for applied machine learning.

However, the paper has serious problems in its present form. The largest problem is the unclear nature of the dataset. The abstract and methods say the dataset is from real observations and from KALRO, but the introduction also says the study uses a “large scale synthetic dataset designed to mimic real world MLND characteristics” . This is a major contradiction. The paper must clearly state if the data are real, synthetic, simulated, or partly synthetic.

The class imbalance is also not very strong. The dataset has 5,400 healthy samples and 2,492 infected samples, with a ratio of about 2.17:1 . This is only moderate imbalance. The title gives strong emphasis to class imbalance, but the paper does not show clearly how imbalance affects the models. There is no clear comparison between models trained with and without SMOTE or class weighting.

The methodology is broad, but not deep enough. Many models are tested, including Logistic Regression, SVM, Random Forest, XGBoost, LightGBM, CatBoost, and MLP. But the paper does not give enough detail about feature extraction, parameter grids, SMOTE application, leakage control, or statistical testing. The paper says SMOTE and class weighting were used , but it does not clearly state whether SMOTE was applied only to the training data and inside each cross-validation fold. This is very important. If SMOTE was applied before train-test split or outside cross-validation, the results may be biased.

The results are useful but over-interpreted. CatBoost has the best accuracy, while Logistic Regression has the best AUC and F1 score in Table 1 . This is interesting, but the authors describe many results as “strong” although the performance is moderate. An AUC around 0.80 and accuracy around 0.75 are promising, but not enough to claim reliable field deployment without external validation.

Reviewer #3: The topic presented in the manuscripts highlights an important and interesting considering the automated MLND detection in maize. However, there are a few major as well as minor concerns that needs to be fully addressed before moving forward.

Major Concerns

==========

1. The manuscript highlights novelty in three key aspects: 1) dataset imbalance, 2) comparative study with numerous ML models, and 3) Utilization of large scale synthetic data to mimic MLND characteristics. (Line # 37-42). There is a need to address the following points which will surely highlight the novelty of the manuscript in a much clearer manner.

- Firstly, majority of real-life datasets such as medical diagnostics or agriculture are normally imbalanced. So, in my opinion there is a need to include a few examples of publicly available balanced datasets existing in this research domain and studies with comparable performances.

- Secondly, the manuscripts states that rigorous comparison of ML algorithms was conducted “bridging the gap between classical and modern approaches” in the MLND detection. In results section, the CatBoost achieved the highest accuracy 75.30%, which is particularly low considering the importance of reliability of disease detection and binary classification problem. Therefore, there is also a need to clearly pin point the key factors contributed in identifying the gaps between classical and modern approaches. Moreover, comparing these ML model performances with some recent modern approaches, particularly DL will enhance the scientific rigor of the study.

- Thirdly, there is a stronger need to elaborate the protocol followed for obtaining the “large scale synthetic data”, that is the core of this manuscript. Additionally, there are confusions regarding the synthetic data; whether this synthetic dataset is a publicly available or it was curated / generated as a part of this research study. If generated then elaboration of techniques will benefit the manuscript’s novelty.

2. Line # 43-44. The manuscript states that “the study develops a structured and scalable dataset for MLND detection based on interpretable visual features”. This line somewhat clarifies the point that the dataset was constructed as a part of this study. However, still there is a stronger need to provide detailed insights into the term “interpretable visual features”, clearly elaborating the source of these visual features such as images or videos. Providing these details will surly enhance the understanding of potential readers.

3. Line # 46-47. The manuscript includes “resource-constrained environments” in the contributions section. Including a few examples of devices with specifications will establish the context properly.

4. The manuscript includes a heading “Model Development” whereas the study only includes comparative analysis of ML models, clearly lacking the novelty component particularly to the model development. In my opinion, experimentation with feature engineering and ML models will increase the scientific rigor of the study.

5. Hyper-parameters for MLP (no. of layers, epochs, optimizer, and learning rate), RF (no. of trees, depth), XGBoost (learning rate, maximum depth, scale_pos_weight), and SVM (kernel type, regularization parameter, and gamma) needs to properly elaborate along with empirical reasons for the hyper-parameter values. Clearly mentioning these hyper-parameters will enhance the reproducibility aspect of this study.

6. The abstract mentions “Results showed that CatBoost (accuracy = 0.753), LightGBM (accuracy = 0.750), and Logistic Regression (AUC = 0.808) achieved the best overall performance”. In results, the recall and F1-score of both LightGBM and CatBoost are quite close; considering these results along with training time and model size, suggesting one best fit model (with logical reasoning) for resource constrained settings will be extremely value-added.

Minor Concerns

==========

1. Line # 85: “algorithms was implemented” needs to be corrected grammatically.

2. Mathematical definitions and elaborations of CatBoost and LightGBM needs to be added to increase the understanding of potential readers, as both these models are regarded as the best performers in the manuscript.

3. It is impressive that the author(s) have included the computational efficiency in terms of training time and inference latency. However, including a collective inference time for the whole test set will also give detailed insights to these ML models, particularly considering the facilities providing batch analysis.

4. One liner definitions of ML models are added alongside mathematical formulae which is a good thing. However, highlighting the reasons for choosing these specific ML models will enhance understanding of readers.

5. Resolution of figures needs to be improved keeping readability easier.

6. Some figures are cropped from the left side (looking into the page).

Overall the topic and content is good. But the manuscript requires considerable amendments for highlighting the novelty and contributions of the study. My recommendation is a major revision at the moment before moving forward.

Reviewer #4: **Major Revision**

The manuscript addresses an important problem in precision agriculture by comparing several machine learning algorithms for Maize Lethal Necrosis Disease (MLND) detection under class imbalance. The topic is relevant and within the scope of PLOS ONE. The manuscript is generally well organized, includes a broad comparison of machine learning algorithms, and incorporates explainable AI techniques (SHAP, LIME, PDP), calibration analysis, and computational efficiency evaluation.

However, several methodological issues substantially weaken the scientific contribution. Most importantly, the manuscript lacks sufficient information regarding the dataset generation and validation, making reproducibility and the reliability of the reported results questionable. Significant revisions are required before the manuscript can be considered for publication.

---

# Major Comments

### 1. Dataset description is inconsistent and unclear

The manuscript states that the dataset consists of **7,892 observations collected from KALRO**, while the Introduction later states that the study utilizes **a large-scale synthetic dataset designed to mimic real-world MLND characteristics.** These two descriptions contradict each other and create confusion regarding the actual data source.

The authors should clearly explain:

* whether the dataset is entirely real,

* entirely synthetic,

* or partially synthetic.

The manuscript should describe exactly how the data were collected or generated.

---

### 2. Lack of detailed feature extraction methodology

The manuscript refers to features such as:

* Chlorosis Intensity

* Green-Yellow Ratio

* Necrotic Area

* Lesion Distribution

* Texture Roughness

However, it never explains how these variables were computed.

The paper should specify:

* image acquisition procedure,

* segmentation pipeline,

* image preprocessing,

* mathematical definitions of features,

* software used,

* whether feature extraction was manual or automated.

Without these details, the work cannot be reproduced.

---

### 3. Insufficient information about class imbalance handling

The authors mention SMOTE and class weighting but omit critical implementation details.

Specifically, they should report:

* which SMOTE variant was used,

* oversampling ratio,

* nearest-neighbor parameter,

* whether SMOTE was applied before or after train-test splitting,

* whether cross-validation was performed after resampling.

Incorrect application of SMOTE can introduce data leakage.

---

### 4. Missing external validation

All results appear to be obtained from a single dataset using an 80/20 split.

There is no validation using:

* an external dataset,

* independent field data,

* or temporal validation.

Consequently, it is difficult to judge the generalizability of the proposed models.

---

### 5. Statistical significance is missing

Although many algorithms are compared, no statistical tests are reported.

The authors should determine whether observed performance differences are statistically significant using methods such as:

* McNemar's test

* DeLong test (AUC)

* Wilcoxon signed-rank test

* Friedman/Nemenyi comparison

Without significance testing, it is difficult to conclude that one model truly outperforms another.

---

### 6. Hyperparameter tuning lacks reproducibility

The manuscript only mentions Grid Search.

The paper should provide:

* parameter search ranges,

* final optimized parameters,

* random seeds,

* stopping criteria,

* software versions.

A supplementary table containing all optimized hyperparameters would greatly improve reproducibility.

---

### 7. Explainability analysis is largely descriptive

The SHAP and LIME analyses simply identify important variables.

The discussion should better connect these findings to:

* plant pathology,

* MLND symptoms,

* biological interpretation,

* previous agronomic studies.

Currently, the explainability section mostly repeats the figures without deeper scientific interpretation.

---

### 8. Novelty should be better justified

The manuscript claims novelty through:

* comparison of ML algorithms,

* class imbalance,

* explainable AI.

However, many recent papers already compare classical ML algorithms and apply SHAP/LIME.

The authors should clearly explain:

* what methodological innovation this work introduces,

* why it advances the state of the art beyond previous comparative studies.

---

# Minor Comments

### 1.

Several grammatical issues remain throughout the manuscript.

Examples include:

* "feature based" → "feature-based"

* "real world" → "real-world"

* "Multi Layer Perceptron" → "Multi-Layer Perceptron"

Professional language editing is recommended.

---

### 2.

Many figures are referenced but their captions remain rather generic.

Captions should contain:

* sample sizes,

* model settings,

* interpretation of major observations.

---

### 3.

The computational environment should include:

* CPU

* RAM

* operating system

* Python version

* package versions

to improve reproducibility.

---

### 4.

The manuscript would benefit from reporting confidence intervals for:

* Accuracy

* AUC

* Precision

* Recall

* F1-score

instead of only point estimates.

---

### 5.

The Discussion section repeats many of the Results instead of critically interpreting them.

It should better discuss:

* practical implications,

* limitations,

* deployment challenges,

* comparison with recent literature.

---

### 6.

Although data availability is provided through Zenodo, the manuscript should also provide:

* source code,

* preprocessing scripts,

* trained models,

to facilitate reproducibility.

---

# Strengths

* Relevant agricultural application.

* Comprehensive comparison of multiple machine learning algorithms.

* Inclusion of calibration analysis.

* Consideration of computational efficiency.

* Use of explainable AI methods (SHAP, LIME, PDP).

* Clear manuscript organization.

---

# Weaknesses

* Dataset description is inconsistent.

* Feature extraction methodology is insufficiently described.

* Limited reproducibility.

* No external validation.

* No statistical significance analysis.

* Limited methodological novelty.

* Explainability discussion requires deeper biological interpretation.

---

**Final Recommendation: Major Revision.**

**********

what does this mean?). If published, this will include your full peer review and any attached files.

If you choose “no”, your identity will remain anonymous but your review may still be made public.

**Do you want your identity to be public for this peer review?**  For information about this choice, including consent withdrawal, please see our Privacy Policy

Reviewer #1: No

Reviewer #2: No

Reviewer #3: No

Reviewer #4: **Yes:**  Jakup Fondaj

**********

[NOTE: If reviewer comments were submitted as an attachment file, they will be attached to this email and accessible via the submission site. Please log into your account, locate the manuscript record, and check for the action link "View Attachments". If this link does not appear, there are no attachment files.]

To ensure your figures meet our technical requirements, please review our figure guidelines: https://journals.plos.org/plosone/s/figures

You may also use PLOS’s free figure tool, NAAS, to help you prepare publication quality figures: https://journals.plos.org/plosone/s/figures#loc-tools-for-figure-preparation.

NAAS will assess whether your figures meet our technical requirements by comparing each figure against our figure specifications.

Attachment Submitted filename: Comments on PONE-D-26-21835.pdf

# Author comment: Author response to Decision Letter 1

**DOI:** 10.1371/journal.pone.0349738.r002

24 Jul 2026

Response to the Academic Editor and Reviewers

We sincerely thank the Academic Editor and all reviewers for their careful evaluation of our manuscript entitled "Comparative Evaluation of Machine Learning Algorithms for Maize Lethal Necrosis Disease Detection Under Class Imbalance" (PONE-D-26-21835). We greatly appreciate the constructive comments and suggestions, which have substantially improved the quality, clarity, methodological rigor, and reproducibility of the manuscript.

In response to the reviewers' recommendations, we have undertaken a comprehensive revision of the manuscript. The major revisions include:

• Clarification of the dataset origin throughout the manuscript and correction of all inconsistencies regarding the data source.

• Revision of the Introduction to better justify the study novelty and clearly position our contribution relative to existing MLND and deep learning studies.

• Expansion of the Methods section to include detailed descriptions of the study design, variables, machine learning implementation, hyperparameter optimisation, class imbalance handling, calibration procedures, computational environment, and reproducibility considerations.

• Addition of an ablation study quantifying the impact of class imbalance handling.

• Inclusion of statistical comparisons using cross-validation summaries and confidence intervals, together with a more cautious interpretation of model differences.

• Regeneration of SHAP figures using the original feature names, inclusion of the missing SHAP beeswarm figure, and expansion of the explainability analysis with biological interpretation.

• Revision of the Discussion to include direct comparisons with previous MLND studies, justification of the structured feature-based approach, practical implications, limitations, and a more balanced conclusion.

• Revision of the Data Availability Statement and clarification of the use of secondary data obtained from the Kenya Agricultural and Livestock Research Organization (KALRO).

• Numerous improvements to figures, tables, grammar, formatting, references, and supplementary materials.

We believe that these revisions have addressed all of the reviewers' concerns and have substantially strengthened the manuscript.

Responses to Reviewer #1

We sincerely thank Reviewer #1 for the exceptionally thorough and constructive review. The comments significantly improved the scientific quality, clarity, and reproducibility of the manuscript. Each comment has been carefully considered and addressed as detailed below.

Comment 1

Dataset Origin is Inconsistent and Unverifiable

Three different descriptions of the dataset appear across three sections: the abstract describes it as "derived from real observations," the introduction calls it "a large-scale synthetic dataset designed to mimic real-world MLND characteristics," and the methods describe it as "secondary data obtained from KALRO." These are mutually exclusive descriptions. The authors must provide one clear, consistent statement of the dataset origin across all sections.

The Zenodo DOI does not resolve to any accessible record, directly violating PLOS One data availability policy. If the data was obtained from KALRO under a formal data use agreement, any sharing restrictions must be explicitly stated. If the dataset is synthetic, it should be freely shareable and the DOI must be corrected before re-submission.

Response

We thank the reviewer for identifying this important inconsistency.

The inconsistency resulted from an earlier draft of the manuscript that referred to a synthetic dataset. This was an oversight and has now been completely corrected throughout the manuscript.

The revised manuscript consistently states that the study used secondary data obtained from the Kenya Agricultural and Livestock Research Organization (KALRO) comprising 7,892 observations collected from field-acquired maize leaf images originating from multiple maize-growing counties in Kenya. No synthetic images or artificially generated samples were used in this study.

To improve clarity, we substantially revised the Abstract, Introduction, Study Design and Data Source, Dataset Construction and Choice, Discussion, Limitations, and Conclusion to consistently describe the dataset as secondary field-derived data obtained from KALRO.

We also expanded the manuscript to explain that the machine learning models operate on engineered visual, texture, lesion, and morphological features extracted from real maize leaf images, rather than raw image pixels.

The Zenodo DOI cited in the original submission is correct since it contains the real data from KALRO. I obtained permission from KALRO to make the dataset publicly available because it contains no sensitive or personally identifiable information.

Comment 2

SMOTE vs. SMOTE-ENN Inconsistency

The methods section describes SMOTE as the oversampling technique. The results section references SMOTE-ENN without prior definition. These are different methods. SMOTE-ENN combines synthetic minority oversampling with Edited Nearest Neighbours undersampling, which removes borderline and noisy majority class samples near decision boundaries. This cleaning step can substantially alter the training data distribution compared to SMOTE alone. If SMOTE-ENN was used, the methods section must describe it fully including the ENN component, the number of neighbours used, and the rationale for choosing the combined approach. The foundational SMOTE paper (Chawla et al., 2002) is absent from the reference list despite SMOTE being a core methodological component.

Response

We appreciate the reviewer highlighting this inconsistency.

The manuscript has been revised to consistently describe the class imbalance methodology used. The Methods section now clearly specifies:

• the exact resampling technique employed;

• the implementation details;

• the oversampling procedure;

• the nearest-neighbour parameter;

• the sequence of preprocessing operations;

• the application of resampling only within the training folds to prevent data leakage;

• and the rationale for combining resampling with class weighting.

The terminology has been made consistent throughout the manuscript, eliminating the previous ambiguity between SMOTE and SMOTE-ENN.

In addition, the foundational work by Chawla et al. (2002) has been added and cited appropriately within both the Methods and Discussion sections.

Comment 3

No Statistical Significance Testing for Model Comparisons

The comparative analysis rests entirely on point estimates. The three top models differ in accuracy by less than 0.005. At this margin, no superiority claim is statistically defensible without formal testing. McNemar test and Friedman test with Nemenyi post-hoc correction are recommended. All metrics in Table 1 should report mean and standard deviation across cross-validation folds rather than single point estimates.

Response

We thank the reviewer for this important suggestion.

The manuscript has been revised to provide a more statistically rigorous comparison of model performance.

Specifically, we now report cross-validation performance using mean values together with standard deviations and 95% confidence intervals, providing a better representation of model variability across folds rather than relying solely on point estimates.

Furthermore, we substantially revised the Results and Discussion to avoid implying superiority based on very small numerical differences. Instead, we emphasise that CatBoost, LightGBM, Random Forest, and Logistic Regression demonstrated comparable predictive performance, with model selection depending on deployment objectives such as interpretability, computational efficiency, calibration, and scalability.

This more conservative interpretation better reflects the observed performance differences and addresses the reviewer's concern regarding overinterpretation.

Comment 4

SHAP Figure Uses Generic Feature Labels

Fig 10 displays only "Feature 0," "Feature 1," through "Feature 10" instead of actual feature names. This indicates the SHAP analysis was run on a preprocessed numpy array where column names were lost after encoding or standardization. The figure directly contradicts the text claiming Chlorosis Intensity, Green Yellow Ratio, and Necrotic Area are the most influential predictors. The authors must use a named pandas DataFrame as input to the SHAP explainer and regenerate Fig 10 with correct feature labels.

Response

We appreciate the reviewer identifying this issue.

The SHAP analysis has been repeated using the original feature-labelled dataset to preserve feature names throughout the explainability pipeline.

Figure 10 has been regenerated and now displays the actual feature names rather than generic labels. The revised figure clearly identifies biologically meaningful predictors including Chlorosis Intensity, Green–Yellow Ratio, Necrotic Area, Infected Distribution, and related visual descriptors.

The accompanying Results and Discussion sections have also been revised to ensure complete consistency between the figures and the textual interpretation.

Comment 5

Fig 11 Was Not Submitted

The SHAP beeswarm plot is referenced and described in detail but was not included in the submission. It must be provided.

Response

We thank the reviewer for noting this omission.

The missing SHAP beeswarm figure has now been included in the revised manuscript.

The associated Results section has been revised to correspond directly with the submitted figure, and the figure caption has been expanded to improve interpretation.

Comment 6

Partial Dependence Plot Text Does Not Match Fig 9

Lines 326–328 state that Fig 9 illustrates the relationship between Greenness Intensity and predicted infection probability. The actual Fig 9 shows PDPs for Chlorosis Intensity, Green Yellow Ratio, and Necrotic Area. The feature "Greenness Intensity" does not exist in the dataset. The text must be corrected.

Response

We appreciate the reviewer identifying this inconsistency.

The text describing the partial dependence plots has been corrected to accurately reflect the features presented in Figure 9.

The revised manuscript now correctly discusses the partial dependence relationships for Chlorosis Intensity, Green–Yellow Ratio, and Necrotic Area, and removes all incorrect references to "Greenness Intensity."

Comment 7

Contradiction Between PDP and SHAP for Green Yellow Ratio

The SHAP description states that lower values of Green Yellow Ratio are associated with higher predicted risk, whereas the PDP shows a monotonically increasing relationship. These two results directly contradict each other. The authors must identify and resolve the source of this contradiction before publication.

Response

We thank the reviewer for this careful observation.

We revisited the explainability analysis and identified that the inconsistency arose from an incorrect interpretation of the SHAP outputs in the original manuscript rather than from the underlying model.

The SHAP analysis, PDP interpretation, and accompanying discussion have all been carefully re-evaluated and rewritten to ensure consistency across all explainability methods.

Furthermore, we substantially expanded the explainability section by incorporating:

• a revised SHAP global importance analysis;

• SHAP interaction analysis;

• SHAP waterfall explanations for representative healthy and infected leaves;

• improved LIME interpretations;

• and stronger biological interpretation linking the identified features to established MLND symptom progression reported in the plant pathology literature.

These revisions provide a consistent and biologically meaningful interpretation of feature effects across SHAP, PDP, and LIME analyses.

Comment 8. No Ablation Study for Class Imbalance Handling

The claim that SMOTE and class weighting contributed to improved minority class detection requires a controlled comparison. No baseline results without imbalance handling are presented. Without this, the contribution of these techniques cannot be quantified. The authors should add baseline experiments for each model without imbalance handling and report the difference in recall and F1 for the minority class.

Response:

Thank you for this valuable suggestion. We have incorporated a comprehensive ablation study comparing model performance with and without class imbalance mitigation. Specifically, we evaluated all classifiers under three settings: (i) no imbalance handling, (ii) class weighting only, and (iii) SMOTE combined with class weighting. The revised Results section now reports the corresponding changes in minority-class Recall, F1-score, Balanced Accuracy, and AUC. The findings demonstrate that class imbalance mitigation consistently improved minority-class detection, particularly for ensemble-based models, thereby providing quantitative evidence supporting our methodological choice.

Comment 9. Hardware Specifications Absent from Scalability Analysis

Table 2 reports training time and inference latency without stating the CPU model, RAM, operating system, or library versions used.

Response:

We appreciate this observation. A dedicated Computational Environment subsection has been added to the Methods section. The revised manuscript now reports the processor specifications, available RAM, operating system, Python version, and major software libraries used during model development and scalability experiments. This information improves reproducibility and enables fair comparison with future studies.

Comment 10. Hyperparameter Values for Final Models Not Reported

Grid search with 5-fold cross-validation is described but the final selected hyperparameter values for all nine models are never reported.

Response:

Thank you for highlighting this reproducibility issue. We have substantially expanded the Hyperparameter Optimization subsection by reporting the search ranges, optimization strategy, cross-validation procedure, random seed, stopping criteria where applicable, and the final optimized hyperparameter values for all evaluated models. In addition, a supplementary table summarizing the optimized hyperparameters has been included to facilitate reproducibility.

Comment 11. Brier Score Misinterpreted

The authors attribute low Brier scores to better calibration. Calibration should be assessed using Expected Calibration Error (ECE) or the Hosmer-Lemeshow test.

Response:

We appreciate this important clarification. The calibration analysis has been revised to distinguish between overall probabilistic accuracy (Brier Score) and probability calibration. In addition to calibration curves, we now report Expected Calibration Error (ECE). The discussion has been revised accordingly to avoid overstating the interpretation of Brier scores and to provide a more rigorous assessment of model calibration.

Comment 12. Comparison with Prior MLND Studies Lacks Specificity

The discussion should include direct numerical comparisons with previous MLND studies and explain why publicly available datasets were not used.

Response:

Thank you for this suggestion. The Comparison with Existing Studies subsection has been substantially expanded. The revised manuscript now includes direct comparisons with recent MLND studies employing deep learning approaches, highlighting methodological differences between image-based deep learning and the structured feature-based machine learning framework adopted in this study. We also clarify that the present study utilizes a secondary dataset obtained from KALRO, which provides structured field-derived observations appropriate for evaluating interpretable machine learning models under realistic agricultural conditions.

Comment 13. Limitations Section is Inadequate

The limitations section should address dataset characteristics, absence of external validation, lack of field deployment testing, practical implications of the reported accuracy, and institutional constraints.

Response:

We thank the reviewer for this recommendation. The Limitations section has been substantially expanded. It now discusses the use of secondary field-derived data, the absence of independent external validation, the lack of real-world deployment experiments, the implications of the observed classification accuracy, and directions for future research, including multi-institutional validation and prospective field evaluation.

Comment 14. Conclusion Overstates Findings

The conclusion describes a system with 0.753 accuracy as reliable and deployable. At this accuracy level approximately one in four samples is misclassified.

Response:

We appreciate this observation. The Conclusion has been carefully revised to present a more balanced interpretation of the findings. Statements implying immediate deployment have been moderated, and the manuscript now emphasizes that the proposed framework represents a promising decision-support approach that requires further external validation before operational deployment. The discussion also explicitly acknowledges the trade-off between predictive performance, interpretability, and computational efficiency.

Minor Comments

Minor Comment 1. Figure 15 LIME has truncated feature labels

Response:

Thank you for identifying this formatting issue. The Figure has been regenerated at higher resolution with enlarged margins and complete feature labels to improve readability.

Minor Comment 2. Figure 8 Learning Curve lacks x-axis label

Response:

The learning curves results were removed.

Minor Comment 3. Model size units are inconsistent

Response:

Thank you for noting this inconsistency. Model sizes are now reported consistently in megabytes (MB) throughout the manuscript, including the scalability table and accompanying discussion.

Minor Comment 4. Grammatical error

"The author declare no conflicts of interest."

Response:

This grammatical error has been corrected to:

"The author declares no conflicts of interest."

Minor Comment 5. Overuse of Reference [3]

Response:

We appreciate this observation. The literature review has been revised by replacing several general citations with more specific primary references that directly support the corresponding methodological and application-related statements.

Minor Comment 6. Inappropriate citation supporting SMOTE

Reference [21], a coronary heart disease prediction paper, is cited to support SMOTE in agricultural disease detection.

Response:

Thank you for identifying this issue. The inappropriate citation has been replaced with the original foundational SMOTE paper by Chawla et al. (2002) together with additional domain-relevant references on imbalanced learning in agricultural machine learning.

Response to Reviewer #2

We sincerely thank the reviewer for the careful assessment of our manuscript and for the constructive comments. We appreciate the recognition of the relevance of the study to precision agriculture and food security. We have carefully revised the manuscript to address all concerns raised. Our point-by-point responses are provided below.

Comment 1. Dataset description is unclear and inconsistent

The largest problem is the unclear nature of the dataset. The abstract and methods say the dataset is from real observations and from KALRO, but the introduction also says the study uses a "large-scale synthetic dataset designed to mimic real world MLND characteristics". This is a major contradiction. The paper must clearly state if the data are real, synthetic, simulated, or partly synthetic.

Response:

We thank the reviewer for identifying this important inconsistency. The contradiction arose from an error in the original Introduction. The manuscript has been thoroughly revised to ensure consistency across all sections. We now clearly state that the study utilizes secondary field-derived data obtained from the Kenya Agricultural and Livestock Research Organization (KALRO) comprising 7,892 observations collected from major maize-growing counties in Kenya. All references describing the dataset as synthetic have been removed. In addition, the Study Design and Data Source subsection has been expanded to clearly describe the data source, study design, variables, and the nature of the structured feature dataset.

Comment 2. The class imbalance is only moderate

The dataset has 5,400 healthy samples and 2,492 infected samples, with a ratio of about 2.17:1. This is only moderate imbalance. The title gives strong emphasis to class imbalance, but the paper does not show clearly how imbalance affects the models. There is no clear comparison between models trained with and without SMOTE or class weighting.

Response:

We appreciate this observation. Although the imbalance ratio is moderate, minority-class misclassification remains a practical concern in disease detection because failing to identify infected plants has significant agricultural consequences. To address this concern, we have added an ablation study comparing classifier performance under three experimental settings: (i) no imbalance handling, (ii) class weighting only, and (iii) SMOTE combined with class weighting. The revised Results section now quantitatively demonstrates the impact of imbalance mitigation on Recall, F1-score, Balanced Accuracy, and AUC, thereby providing evidence of the contribution of these techniques.

Comment 3. Methodology lacks sufficient implementation details

Many models are tested, including Logistic Regression, SVM, Random Forest, XGBoost, LightGBM, CatBoost, and MLP. But the paper does not give enough detail about feature extraction, parameter grids, SMOTE application, leakage control, or statistical testing. The paper says SMOTE and class weighting were used, but it does not clearly state whether SMOTE was applied only to the training data and inside each cross-validation fold.

Response:

We thank the reviewer for this valuable recommendation. The Methods section has been substantially expanded to improve transparency and reproducibility. Specifically, we now provide:

• a clearer description of the structured feature dataset and variables;

• detailed information on the SMOTE implementation, including the variant used, oversampling parameters, and the application of SMOTE exclusively to the training data within each cross-validation fold to prevent data leakage;

• comprehensive descriptions of the hyperparameter search space, optimized hyperparameter values, random seed, software versions, and computational environment; and

• statistical significance analyses, including McNemar's test, DeLong's test, Wilcoxon signed-rank test, and Friedman/Nemenyi comparisons, together with confidence intervals for the reported performance metrics.

These additions substantially improve the reproducibility and methodological rigor of the study.

Comment 4. The results are useful but over-interpreted

CatBoost has the best accuracy, while Logistic Regression has the best AUC and F1 score. This is interesting, but the authors describe many results as "strong" although the performance is moderate. An AUC around 0.80 and accuracy around 0.75 are promising, but not enough to claim reliable field deployment without external validation.

Response:

We appreciate this thoughtful comment and agree that our original interpretation was overly optimistic. The Abstract, Discussion, and Conclusion have been carefully revised to provide a more balanced interpretation of the findings. Rather than describing the models as "reliable" or immediately suitable for deployment, we now characterize the reported performance as promising within the context of structured feature-based machine learning. We explicitly acknowledge that external validation and prospective field evaluation remain necessary before operational deployment. The Discussion also emphasizes the trade-offs between predictive performance, interpretability, calibration, and computational efficiency, particularly for resource-constrained agricultural settings.

Response to Reviewer #3

We sincerely thank the reviewer for the insightful and constructive comments. We appreciate the positive assessment of the importance of the research topic and the valuable suggestions for strengthening the manuscript. We have carefully revised the manuscript to address all major and minor concerns. Our detailed responses are provided below.

Major Comments

Comment 1. Novelty should be better justified

The manuscript highlights novelty in three key aspects: (1) dataset imbalance, (2) comparative study with numerous ML models, and (3) utilization of large-scale synthetic data to mimic MLND characteristics. There is a need to address the following points which will surely highlight the novelty of the manuscript in a much clearer manner.

Firstly, majority of real-life datasets such as medical diagnostics or agriculture are normally imbalanced. There is a need to include examples of publicly available balanced datasets existing in this research domain and studies with comparable performances.

Secondly, the manuscript states that rigorous comparison of ML algorithms was conducted "bridging the gap between classical and modern approaches." However, CatBoost achieved only 75.30% accuracy. The manuscript should clearly identify the factors contributing to the gap between classical and modern approaches. Moreover, comparing these machine learning models with recent deep learning approaches would enhance the scientific rigor.

Thirdly, there is a stronger need to elaborate the protocol followed for obtaining the "large-scale synthetic data" and clarify whether the dataset is publicly available or was generated as part of this study.

Response:

We sincerely thank the reviewer for these valuable observations. The Introduction has been substantially revised to better justify the novelty and positioning of the study.

First, we now discuss the prevalence of class imbalance in agricultural disease datasets while acknowledging that moderate imbalance is common in real-world applications. We also include recent literature to contextualize the problem and clarify the motivation for evaluating imbalance mitigation techniques.

Second, the comparison with existing studies has been significantly expanded. The revised Discussion now directly compares the performance of the evaluated machine learning models with recent deep learning approaches for MLND detection. We explain that the objective of this study was not to outperform deep learning methods in classification accuracy, but rather to evaluate the trade-offs between predictive performance, interpretability, computational efficiency, calibration, and deployment suitability using structured feature-based machine learning. This discussion clarifies the practical gap addressed between classical and modern machine learning approaches.

Third, we have corrected the dataset description throughout the manuscript. The dataset is not synthetic. All references to synthetic data have been removed, and the manuscript now consistently states that the study uses secondary field-derived data obtained from the Kenya Agricultural and Livestock Research Organization (KALRO). The Study Design and Data Source section has been expanded accordingly.

Comment 2. The manuscript states that the study develops a structured and scalable dataset but does not explain the interpretable visual features.

There is a stronger need to provide detailed insights into the term "interpretable visual features", clearly elaborating the source of these visual features such as images or videos.

Response:

Thank you for this important suggestion. The Study Design and Data Source and Variables and Feature Description sections have been expanded to provide a clearer description of the structured dataset and the engineered visual descriptors. We now explicitly state that the dataset consists of structured quantitative variables representing colour, texture, lesion morphology, and spatial characteristics of maize leaves obtained from secondary KALRO data. We further clarify that the study evaluates machine learning models using these structured descriptors rather than performing image processing or feature extraction within the present work.

Comment 3. Resource-constrained environments should be better contextualized

Including a few examples of devices with specifications will establish the context properly.

Response:

We appreciate this recommendation. The manuscript has been revised to better contextualize resource-constrained deployment scenarios by discussing lightweight computing platforms typically used in precision agriculture, including mobile devices, edge computing platforms, and single-board computers such as Raspberry Pi-class hardware. The scalability analysis now better explains how computational efficiency, memory usage, and inference latency influence practical deployment in such environments.

Comment 4. The heading "Model Development" is misleading

The manuscript includes a heading "Model Development" whereas the study only includes comparative analysis of ML models.

Response:

We thank the reviewer for this observation. The section has been renamed "Machine Learning Model Implementation" to more accurately reflect the scope of the study. The revised section now explains the rationale for selecting the different machine learning algorithms and clarifies that the objective was a comparative evaluation using a common structured feature representation rather than proposing a novel machine learning model.

Comment 5. Hyperparameters should be fully reported

Hyperparameters for MLP, RF, XGBoost, and SVM need to be elaborated together with empirical reasons for the selected values.

Response:

Thank you for this valuable suggestion. The hyperparameter optimization section has been substantially expanded. The revised manuscript now reports the parameter search ranges, optimization strategy, final optimized hyperparameter values, random seed, stopping criteria where applicable, and the rationale for selecting these parameters.

Comment 6. Recommend one best model for resource-constrained settings

Considering recall, F1-score, training time, and model size, suggesting one best-fit model with logical reasoning for resource-constrained settings would be extremely valuable.

Response:

We appreciate this excellent suggestion. The Abstract, Discussion, and Scalability Analysis have been revised accordingly. While CatBoost achieved the highest classification accuracy, the revised manuscript now concludes that LightGBM provides the best overall balance between predictive performance, computational efficiency, model size, and inference speed, making it the most suitable candidate for deployment in resource-constrained agricultural environments. This recommendation is supported by the combined evaluation of predictive accuracy, calibration, scalability, and computational cost rather than classification performance alone.

Minor Comments

Comment 1. Line #85: "algorithms was implemented" should be corrected grammatically.

Response:

Thank you for identifying this error. The grammatical mistake has been corrected throughout the manuscript.

Comment 2. Mathematical definitions of CatBoost and LightGBM should be added.

Response:

We appreciate this recommendation. Mathematical formulations and accompanying descriptions for both CatBoost and LightGBM have been added to the Machine Learning Model Implementation section to improve methodological completeness and enhance reader understanding of the best-performing models.

Comment 3. Include collective inference time for the entire test set

Including inference time for the whole test set would provide additional insights.

Response:

We thank the reviewer for this useful suggestion. In addition to reporting per-sample inference latency, the scalability analysis has been expanded to discuss the computational implications for batch prediction and practical deployment scenarios. This provides a more comprehensive evaluation of model efficiency during large-scale inference.

Comment 4. Explain why these specific ML models were selected

Highlighting the reasons for choosing these specific ML models will enhance understanding.

Response:

Thank you for this suggestion. The Machine Learning Model Implementation section has been revised to explain the rationale for selecting each class of algorithms. The evaluated models were chosen to represent diverse machine learning paradigms—including linear, kernel-based, tree-based, ensemble, and neural network methods—allowing a comprehensive comparison of predictive performance, interpretability, computational efficiency, and robustness using a common structured feature representation.

Comment 5. Resolution of figures should be improved.

Response:

We appreciate this recommendation. All figures have been regenerated at higher resolution to improve readability and comply with the journal's figure quality requirements.

Comment 6. Some figures are cropped from the left side.

Response:

Thank you for identifying this formatting issue. The affected figures have been resized and reformatted to ensure that all labels, axes, and annotations are fully visible in the revised manuscript.

We sincerely thank Reviewer #3 for the constructive comments and recommendations. Addressing these suggestions has substantially strengthened the manuscript by improving its methodological clarity, novelty justification, reproducibility, interpretability, and practical relevance.

Response to Reviewer #4

We sincerely thank the reviewer for the thorough and constructive assessment of our manuscript. We appreciate the recognition of the relevance of the research problem and the positive comments regarding the comprehensive evaluation of machine learning models, explainable AI, calibration analysis, and computational efficiency. We have carefully revised the manuscript to address all concerns raised. Our detailed responses are provided below.

Major Comments

Comment 1. Dataset description is inconsistent and unclear

The manuscript states that the dataset consists of 7,892 observations collected from KALRO, while the Introduction later states that the study utilizes a large-scale synthetic dataset designed to mimic real-world MLND characteristics. These two descriptions contradict each other and create confusion regarding the actual data source. The authors should clearly explain whether the dataset is entirely real, entirely synthetic, or partially synthetic. The manuscript should describe exactly how the data were collected or generated.

Response:

We thank the reviewer for identifying this important inconsistency. The contradiction resulted from an inadvertent statement retained in the Introduction. The manuscript has been comprehensively revised to ensure consistency across all sections. We now clearly state that the study uses secondary field-derived data obtained from the Kenya Agricultural and Livestock Research Organization (KALRO) comprising 7,892 observations collected from major maize-growing counties in Kenya. All references describing the dataset as synthetic have been removed. In addition, the Study Design and Data Source section has been substantially expanded to describe the study design, data source, variables, and characteristics of the structured feature dataset.

Comment 2. Lack of detailed feature extraction methodology

The manuscript refers to features such as Chlorosis Intensity, Green-Yellow Ratio, Necrotic Area, Lesion Distribution, and Texture Roughness but never explains how these variables were computed. The paper should specify image acquisition, segmentation, preprocessing, mathematical definitions, software used, and whether feature extraction was manual or automated.

Response:

We appreciate the reviewer's concern. The present study utilizes secondary structured data obtained from KALRO, and the feature extraction process was performed prior to data acquisition. Consequently, the original image acquisition, segmentation pipeline, preprocessing procedures, and feature engineering algorithms are beyond the scope of this study and were not available to the authors. To avoid any ambiguity, the manuscript now explicitly states that the study evaluates machine learning models using pre-extracted structured visual, texture, lesion, and morphological descriptors provided within the secondary dataset rather than performing feature extraction from raw images. The Study Design and Data Source and Variables and Feature Description sections have been revised accordingly to clearly describe the available variables and their role in model development.

Comment 3. Insufficient information about class imbalance handling

The authors mention SMOTE and class weighting but omit critical implementation details.

Response:

Thank you for this important observation. The Methods section has been substantially expanded to provide complete implementation details. The revised manuscript now specifies the SMOTE variant employed, oversampling parameters, nearest-neighbor settings, application of SMOTE exclusively to the training data within each cross-validation fold to prevent data leakage, and the complete cross-validation workflow. These additions substantially improve methodological transparency and reproducibility.

Comment 4. Missing external validation

All results appear to be obtained from a single dataset using an 80/20 split. There is no validation using an external dataset, independent field data, or temporal validation.

Response:

We appreciate this valuable suggestion. To strengthen the assessment of model generalizability, we incorporated Leave-One-County-Out Cross-Validation (LOCO-CV), whereby models were trained using data from all counties except one and subsequently evaluated on the held-out county. This geographic validation provides a more rigorous assessment of spatial generalizability across maize-growing regions in Kenya. We have also explicitly acknowledged the absence of an independent external dataset as a limitation and recommend external validation using datasets collected from different geographical regions and growing seasons in future work.

Comment 5. Statistical significance is missing

Although many algorithms are compared, no statistical tests are reported.

Response:

We thank the reviewer for this important recommendation. The statistical analysis has been substantially strengthened. The revised manuscript now includes McNemar's test, DeLong's test for AUC comparisons, Wilcoxon signed-rank tests, and Friedman tests with Nemenyi post-hoc comparisons where appropriate. In addition, 95% confidence intervals are reported for the principal performance metrics. These additions provide statistical evidence supporting the comparative conclusions presented in the manuscript.

Comment 6. Hyperparameter tuning lacks reproducibility

The manuscript only mentions Grid Search.

Response:

We appreciate this recommendation. The hyperparameter optimization procedure has been comprehensively expanded to include the search ranges, optimization strategy, final optimized hyperparameter values, random seed, stopping criteria, software versions, and computational environment. Furthermore, a supplementary table containing the optimized hyperparameters for all evaluated models has been included to improve reproducibility.

Comment 7. Explainability analysis is largely descriptive

The SHAP and LIME analyses simply identify important variables. The discussion should better connect these findings to plant pathology, MLND symptoms, biological interpretation, and previous agronomic studies.

Response:

We thank the reviewer for this valuable suggestion. The explainability section has been substantially revised to provide deeper biological interpretation of the SHAP, LIME, Partial Dependence Plot, and SHAP interaction analyses. The revised discussion now explicitly relates the identified predictors—including chlorosis intensity, green-yellow ratio, necrotic area, lesion distribution, and texture characteristics—to the known progression and symptomatology of MLND, supported by recent plant pathology and agricultural literature. These revisions considerably strengthen the scientific interpretation of the explainability results.

Comment 8. Novelty should be better justified

The manuscript claims novelty through comparison of ML algorithms, class imbalance, and explainable AI. However, many recent papers already compare classical ML algorithms and apply SHAP/LIME. The authors should clearly explain what methodological innovation this work introduces.

Response:

We appreciate this important observation. The Introduction has been extensively revised to better articulate the contribution of the study. The manuscript now clarifies that the novelty lies not in the individual machine learning algorithms or explainability techniques, but in their integrated application within a unified evaluation framework for MLND detection. Specifically, the revised manuscript highlights the comprehensive evaluation of diverse machine learning paradigms using structured field-derived features, incorporation of class imbalance mitigation, calibration analysis, complementary explainability methods, geographic validation through LOCO-CV, and scalability assessment within a single reproducible framework. This clarification better positions the study relative to existing literature.

Minor Comments

Comment 1. Several grammatical issues remain throughout the manuscript.

Response:

Thank you for this recommendation. The entire manuscript has undergone comprehensive language editing to improve grammar, consistency, and readability. Hyphenation and terminology (e.g., feature-based, real-world, Multi-Layer Perceptron) have been standardized throughout.

Comment 2. Figure captions should be more informative.

Response:

We appreciate this suggestion. All figure captions have been revised to provide additional methodological context, including descriptions of the displayed analyses, principal observations, and interpretation of the results where appropriate.

Comment 3. The computational environment should be reported.

Response:

Thank you for this recommendation. A dedicated Computational Environment subsection has been added to the Methods section, reporting the processor, memory specifications, operating system, Python version, and software library versions used for model development and evaluation.

Comment 4. Report confidence intervals.

Response:

I appreciate this suggestion. The revised manuscript now reports 95% confidence intervals for the principal performance metrics, including Accuracy, AUC, Precision, Recall, and F1-score, providing a more rigorous assessment of model uncertainty.

Comment 5. The Discussion repeats the Results.

Response:

Thank you for this constructive observation. The Discussion has been substantially revised to focus on critical interpretation rather than repetition of numerical results. The revised section now emphasizes the practical implications of the findings, biological interpretation of the explainability analyses, comparison with recent literature, deployment considerations, and study limitations.

Comment 6. Source code and trained models should be shared.

Response: We appreciate the reviewer's recommendation. To further improve the reproducibility and transparency of this study, the complete implementation has been made publicly available. The source code, preprocessing scripts, statistical analysis scripts, explainable AI (SHAP, LIME, and PDP) implementations, and trained machine learning models have been deposited in a publicly accessible Zenodo repository, available at https://doi.org/10.5281/zenodo.21473059.

Closing Statement

I sincerely thank the Academic Editor and all four reviewers for their thorough, thoughtful, and constructive comments. Their feedback has substantially strengthened the manuscript by improving its methodological rigor, reproducibility, statistical analysis, scientific interpretation, clarity, and practical relevance.

In response to the reviewers' recommendations, we have comprehensively revised the manuscript by clarifying the data source, expanding the methodological descriptions, strengthening the statistical analyses, improving the explainability and discussion sections, enhancing the novelty statement, incorporating geographic validation, reporting confidence intervals and optimized hyperparameters, expanding the scalability analysis, and improving the overall presentation of the manuscript.

I believe that these extensive revisions have addressed the reviewers' concerns and have significantly improved the quality and scientific contribution of the manuscript. I am grateful for the opportunity to revise my work and respectfully submit the revised manuscript for your consideration.

Attachment Submitted filename: Response to Reviewer Comments.docx

# Peer review: Decision Letter 1

**DOI:** 10.1371/journal.pone.0349738.r003

**Authors:** Hilary Izuchukwu Okagbue

4 Sep 2026

Dear Dr. Wanyonyi,

Thank you for submitting your manuscript to PLOS One. After careful consideration, we feel that it has merit but does not fully meet PLOS One’s publication criteria as it currently stands. Therefore, we invite you to submit a revised version of the manuscript that addresses the points raised during the review process.

==============================

Please submit your revised manuscript by Nov 03 2026 11:59PM. If you will need more time than this to complete your revisions, please reply to this message or contact the journal office at plosone@plos.org. When you're ready to submit your revision, log on to https://www.editorialmanager.com/pone/ and select the 'Submissions Needing Revision' folder to locate your manuscript file.

- A letter that responds to each point raised by the academic editor and reviewer(s). You should upload this letter as a separate file labeled 'Response to Reviewers'.
- A marked-up copy of your manuscript that highlights changes made to the original version. You should upload this as a separate file labeled 'Revised Manuscript with Track Changes'.
- An unmarked version of your revised paper without tracked changes. You should upload this as a separate file labeled 'Manuscript'.

If you would like to make changes to your financial disclosure, please include your updated statement in your cover letter. Guidelines for resubmitting your figure files are available below the reviewer comments at the end of this letter.

If applicable, we recommend that you deposit your laboratory protocols in protocols.io to enhance the reproducibility of your results. Protocols.io assigns your protocol its own identifier (DOI) so that it can be cited independently in the future. For instructions see: https://journals.plos.org/plosone/s/submission-guidelines#loc-laboratory-protocols. Additionally, PLOS One offers an option for publishing peer-reviewed Lab Protocol articles, which describe protocols hosted on protocols.io. Read more information on sharing protocols at https://plos.org/protocols?utm_medium=editorial-email&utm_source=authorletters&utm_campaign=protocols.

As the corresponding author, your ORCID iD is verified in the submission system and will appear in the published article. PLOS supports the use of ORCID, and we encourage all coauthors to register for an ORCID iD and use it as well. Please encourage your coauthors to verify their ORCID iD within the submission system before final acceptance, as unverified ORCID iDs will not appear in the published article. *Only*  the individual author can complete the verification step; PLOS staff *cannot*  verify ORCID iDs on behalf of authors.

We look forward to receiving your revised manuscript.

Kind regards,

Hilary Izuchukwu Okagbue, Ph.D

Academic Editor

PLOS One

Journal Requirements:

If the reviewer comments include a recommendation to cite specific previously published works, please review and evaluate these publications to determine whether they are relevant and should be cited. There is no requirement to cite these works unless the editor has indicated otherwise.

[Note: HTML markup is below. Please do not edit.]

Reviewers' comments:

Reviewer's Responses to Questions

**Comments to the Author**

Reviewer #1: (No Response)

Reviewer #3: (No Response)

**********

2. Is the manuscript technically sound, and do the data support the conclusions?

Reviewer #1: Partly

Reviewer #3: Yes

**********

3. Has the statistical analysis been performed appropriately and rigorously?

Reviewer #1: Yes

Reviewer #3: Yes

**********

4. Have the authors made all data underlying the findings in their manuscript fully available?

The PLOS Data policy

Reviewer #1: No

Reviewer #3: Yes

**********

5. Is the manuscript presented in an intelligible fashion and written in standard English?

Reviewer #1: Yes

Reviewer #3: Yes

**********

Reviewer #1: Manuscript: PONE-D-26-21835R1

Title: Comparative Evaluation of Machine Learning Algorithms for Maize Lethal Necrosis Disease Detection Under Class Imbalance

OVERVIEW

The authors have undertaken a substantial and genuine revision of the manuscript. The majority of the critical and moderate concerns raised in the first round of review have been addressed effectively. The revised manuscript represents a considerably stronger piece of work. However, two unresolved issues require correction before the manuscript can be accepted, and one moderate concern warrants a response from the authors.

ISSUES RESOLVED IN THIS REVISION

The following concerns from Round 1 have been satisfactorily addressed:

1. Dataset origin: The manuscript now consistently describes the dataset as secondary field-derived data obtained from KALRO across all sections. All references to synthetic data have been removed. The description is clear and consistent throughout.

2. Statistical significance testing: The revised manuscript includes McNemar's test, bootstrap AUC comparison, Wilcoxon signed-rank test, and the Friedman test with Nemenyi post-hoc correction. Table 4 summarizes these results clearly. Importantly, the statistical tests confirm that the top-performing models (Logistic Regression, Random Forest, LightGBM, CatBoost) do not differ significantly, and the authors now interpret this correctly by recommending model selection based on deployment objectives rather than small differences in point estimates. This is a substantial improvement.

3. Confidence intervals: Table 2 now reports 95% confidence intervals for all performance metrics across all nine models. This is the appropriate standard for comparative machine learning studies.

4. SHAP figures: Fig 10 has been regenerated with actual feature names. The missing beeswarm plot (Fig 11) has been included. The PDP text now correctly describes Chlorosis Intensity, Green-Yellow Ratio, and Necrotic Area. The Green Yellow Ratio contradiction between PDP and SHAP has been resolved and the biological interpretation has been substantially expanded.

5. Ablation study: Table 5 quantifies the impact of SMOTE and class weighting versus no imbalance handling for all nine models. The gains in minority class recall are clearly reported, with Logistic Regression showing +16.3% and KNN showing +17.5%. This directly addresses the previously unsubstantiated claim about the contribution of these techniques.

6. Calibration: Table 6 now reports Expected Calibration Error (ECE) before and after Platt scaling and isotonic regression for all models. The distinction between the Brier score as a measure of probabilistic accuracy and ECE as a calibration metric is now correctly maintained throughout the manuscript.

7. Hardware specifications: The computational environment subsection now reports the Intel Core i5-7Y54 CPU at 1.20 GHz, 8 GB RAM, Windows 11 Pro, Python 3.13.5, and exact library versions for all packages used.

8. Hyperparameter values: Table 1 reports the full hyperparameter search ranges for all nine models. A supplementary table of final optimized values has been included. The random seed (42) and stopping criteria are explicitly stated.

9. Geographic validation: Leave-One-County-Out Cross-Validation (LOCO-CV) has been added. Table 3 reports mean and standard deviation of performance across counties. The top models show AUC standard deviations below 0.01 across counties, indicating consistent geographic generalizability within the dataset.

10. Limitations section: The limitations section has been substantially expanded to address the single-institution constraint, absence of external validation, lack of field deployment, the practical implications of 75.3% accuracy, and the need for multi-institutional future work. This is now appropriate in scope and honesty.

11. Conclusion: The conclusion has been rewritten with appropriate qualification. It explicitly states that models should be regarded as decision-support tools rather than standalone diagnostic systems, and that model predictions should complement expert assessment. The word "reliable" is no longer used without qualification.

12. Fig 15 LIME labels, grammar, and minor issues: The LIME figure has been regenerated with complete feature labels. The grammatical error in the Ethical Statement has been corrected. Model size units are now consistent in MB. Reference [21] has been replaced with the foundational Chawla et al. (2002) SMOTE citation.

REMAINING ISSUES REQUIRING CORRECTION

Issue 1: SMOTE-ENN Reference Persists in the Revised Manuscript Body

The methods section now explicitly states: "No Edited Nearest Neighbors (ENN) undersampling or SMOTE-ENN hybrid resampling was performed in this study." This is correct. However, the revised results section still contains the following sentence: "These findings support the use of imbalance handling techniques such as SMOTE-ENN and provide a strong foundation for subsequent comparative machine learning analyses."

This sentence directly contradicts the methods statement above. The authors explicitly state SMOTE-ENN was not used, yet recommend it in the results section. This is the same inconsistency from Round 1, relocated rather than removed. The phrase "such as SMOTE-ENN" must be deleted or replaced with the technique that was actually used (SMOTE with class weighting). This is a straightforward one-sentence correction.

Issue 2: Data Availability Statement Still References the Original Unresolved DOI

The Data Availability Statement in the revised submission still points to https://doi.org/10.5281/zenodo.19997730, which was identified in Round 1 as non-resolving. In the response to reviewers, the author states that source code and trained models have been deposited at a new Zenodo repository: https://doi.org/10.5281/zenodo.21473059. However, this new DOI appears only in the response letter and not in the manuscript's Data Availability Statement.

The Data Availability Statement must be updated to include the correct and verified DOI for the dataset. If the dataset and code are at different DOIs, both must be listed explicitly. PLOS One requires that all data underlying the findings be accessible via a working link before publication. The current Data Availability Statement does not meet this requirement as submitted.

MODERATE CONCERN: Feature Extraction Methodology Remains Unverifiable

Reviewer 4 raised the concern that the manuscript does not explain how the features (Chlorosis Intensity, Green-Yellow Ratio, Necrotic Area) were computed from images. The authors' response states that feature extraction was performed by KALRO prior to data acquisition and is therefore beyond the scope of this study. This is an acceptable position for secondary data. However, users who wish to apply this framework to new images would need to either obtain KALRO's feature extraction pipeline or develop their own. One sentence acknowledging this in the Limitations section would complete the transparency of the manuscript.

SUMMARY

This revision demonstrates that the authors engaged seriously and constructively with the Round 1 review. The statistical analysis is now rigorous, the XAI analysis is verifiable and biologically grounded, the calibration analysis is correctly framed, the ablation study provides genuine evidence for the imbalance handling contribution, and the geographic validation through LOCO-CV adds meaningful value. The conclusions are now appropriately qualified.

Two corrections are required before acceptance: the SMOTE-ENN sentence in the results section must be corrected to reflect what was actually done, and the Data Availability Statement must be updated with a working, verified DOI. Both are straightforward editorial corrections that do not require re-analysis.

Reviewer #3: The revised manuscript has been improved considerably. However, there are still few concerns:

Major Concerns

1. The manuscript mentions “resource-constrained environments” in various sections. Although the detailed specifications of hardware used for the experimental study is included. Still, adding a few examples of platforms suitable for real-time operations will establish the context properly.

2. The manuscript mentions “secondary dataset” multiple times in the entire manuscript. (line # 100, 106, 344 etc). However, the repository records (https://zenodo.org/records/19997730) again create certain confusions about the data source. As the entire study is based on it, so the authors should provide more details on it.

Minor Concerns

1. The same abbreviations such as MLND (Line # 99, Line # 4, Line # 358) has been defined multiple times.

2. Providing access link of the repository

Rest of the manuscript has been improved. My recommendation is a minor revision at the moment before moving forward.

**********

what does this mean?). If published, this will include your full peer review and any attached files.

If you choose “no”, your identity will remain anonymous but your review may still be made public.

**Do you want your identity to be public for this peer review?**  For information about this choice, including consent withdrawal, please see our Privacy Policy

Reviewer #1: No

Reviewer #3: No

**********

[NOTE: If reviewer comments were submitted as an attachment file, they will be attached to this email and accessible via the submission site. Please log into your account, locate the manuscript record, and check for the action link "View Attachments". If this link does not appear, there are no attachment files.]

To ensure your figures meet our technical requirements, please review our figure guidelines: https://journals.plos.org/plosone/s/figures

You may also use PLOS’s free figure tool, NAAS, to help you prepare publication quality figures: https://journals.plos.org/plosone/s/figures#loc-tools-for-figure-preparation.

NAAS will assess whether your figures meet our technical requirements by comparing each figure against our figure specifications.

Attachment Submitted filename: Comments on PONE-D-26-21835_R1.docx

# Author comment: Author response to Decision Letter 2

**DOI:** 10.1371/journal.pone.0349738.r004

7 Sep 2026

Response to Reviewers

Manuscript ID: PONE-D-26-21835R1

Title: Comparative Evaluation of Machine Learning Algorithms for Maize Lethal Necrosis Disease Detection Under Class Imbalance

Dear Dr. Okagbue and Reviewers,

We thank the Academic Editor and the reviewers for their thoughtful and constructive comments on my manuscript. I am grateful for the detailed feedback, which has helped me substantially improve the quality of my work. I have carefully addressed all remaining concerns raised by the reviewers. Below, I provide a point-by-point response to each comment.

Reviewer #1

I thank Reviewer #1 for the thorough and encouraging evaluation of our revised manuscript. I am pleased that the reviewer acknowledged the substantial improvements made in response to the first round of reviews. I have carefully addressed the two remaining issues and the moderate concern as detailed below.

Issue 1: SMOTE-ENN Reference in the Results Section

Reviewer's Comment: The methods section correctly states that no SMOTE-ENN hybrid resampling was performed. However, the results section still contains the sentence: "These findings support the use of imbalance handling techniques such as SMOTE-ENN and provide a strong foundation for subsequent comparative machine learning analyses." This contradicts the methods statement and must be corrected.

Author's Response: I thank the reviewer for identifying this inconsistency. I have corrected the sentence in the Exploratory Data Analysis subsection of the Results section. The phrase "SMOTE-ENN" has been replaced with "SMOTE," which is the technique actually used in this study.

Issue 2: Data Availability Statement

Reviewer's Comment: The Data Availability Statement still points to https://doi.org/10.5281/zenodo.19997730, which the reviewer identified as non-resolving. The new DOI for code and models (https://doi.org/10.5281/zenodo.21473059) appears only in the response letter and not in the manuscript. Both DOIs must be listed explicitly if the data and code are in different locations.

Author's Response: I thank the reviewer for this important clarification. Upon re-verification, I confirm that the dataset DOI (https://doi.org/10.5281/zenodo.19997730) is active and correctly points to the dataset used in this study. I have updated the Data Availability Statement to explicitly list both the dataset DOI and the separate DOI for the source code and trained models. The statement now clearly informs readers where to access both the data and the code.

Revised Text in the Manuscript

"The dataset used in this study is publicly available in the Zenodo repository at (https://doi.org/10.5281/zenodo.19997730). The source code and trained machine-learning models used for the analysis are also publicly available in a separate Zenodo repository at (https://doi.org/10.5281/zenodo.21473059)."

Moderate Concern: Feature Extraction Methodology

Reviewer's Comment: The manuscript does not explain how the features (Chlorosis Intensity, Green-Yellow Ratio, Necrotic Area) were computed from images. While the authors state that feature extraction was performed by KALRO prior to data acquisition and is beyond the scope of this study, one sentence acknowledging this limitation would complete the transparency of the manuscript.

Author's Response: I thank the reviewer for this important suggestion. I have added a new point to the Limitations section acknowledging that the feature extraction procedure was performed by KALRO prior to data acquisition and is not publicly available. I explicitly note that researchers seeking to apply this framework to new image collections would need to either obtain KALRO's feature extraction pipeline or develop their own equivalent feature engineering workflow.

Reviewer #3

I thank Reviewer #3 for acknowledging the improvements made to the manuscript. I have carefully addressed all remaining concerns as detailed below.

Major Concern 1: Resource-Constrained Environments

Reviewer's Comment: The manuscript mentions "resource-constrained environments" in various sections. Although the detailed specifications of hardware used for the experimental study are included, adding a few examples of platforms suitable for real-time operations will establish the context properly.

Author's Response: I thank the reviewer for this valuable suggestion. I have added two sentences to the "Scalability and Practical Implications" subsection of the Discussion to provide specific recommendations for real-world deployment platforms. Based on the scalability analysis, I now suggest that lightweight models such as Logistic Regression and LightGBM are suitable for mid-range smartphones and Raspberry Pi 4 devices, while CatBoost and Random Forest may be better suited for cloud-based virtual machines or edge servers with moderate computational resources.

Major Concern 2: Data Source Confusion

Reviewer's Comment: The manuscript mentions "secondary dataset" multiple times. However, the repository records (https://zenodo.org/records/19997730) create certain confusions about the data source. As the entire study is based on it, the authors should provide more details on it.

Author's Response: I thank the reviewer for this important clarification. I have substantially expanded the "Study Design and Data Source" subsection in the Materials and Methods to provide comprehensive details about the dataset's origin. I now explicitly describe: (1) that the original images were acquired from farmers' fields across multiple maize-growing counties in Kenya under varying field conditions, (2) that KALRO plant pathologists visually confirmed MLND infection status based on established symptom criteria, (3) that the structured features were extracted from the images using KALRO's proprietary image analysis pipeline, (4) that the resulting dataset was provided to the author in an anonymized, tabular format containing only the engineered features and associated labels, with no raw images or identifiable farmer information included, and (5) that this secondary dataset formed the basis for all analyses in the present study. I also clarify that the study focuses on comparative algorithm evaluation using these provided features, not on developing new feature extraction methods.

Minor Concern 1: Duplicate MLND Definitions

Reviewer's Comment: The same abbreviations such as MLND (Line # 99, Line # 4, Line # 358) have been defined multiple times.

Author's Response: I thank the reviewer for this suggestion. I have verified that "Maize Lethal Necrosis Disease (MLND)" is defined once in the Abstract and once at its first mention in the Introduction. All subsequent occurrences throughout the manuscript use only the abbreviation "MLND." No duplicate full definitions remain.

Minor Concern 2: Repository Access Link

Reviewer's Comment: Provide an access link to the repository.

Author's Response: I thank the reviewer for this suggestion. I have ensured that the repository links are prominently and correctly provided in the manuscript. A dedicated Data Availability Statement has been added after the Conclusion section, which includes the dataset DOI (https://doi.org/10.5281/zenodo.19997730) and the separate DOI for the source code and trained models (https://doi.org/10.5281/zenodo.21473059). Both links have been verified and are active.

I believe the revised manuscript now fully addresses all remaining concerns raised by the reviewers. I am grateful for the time and effort the reviewers and editors have invested in improving my work. I look forward to your favorable decision.

Yours sincerely,

Maurice Wanyonyi

Corresponding Author

University of Embu, Kenya

Email: iamwanyonyi93@gmail.com

Attachment Submitted filename: Response to Reviewers.docx

# Peer review: Decision Letter 2

**DOI:** 10.1371/journal.pone.0349738.r005

**Authors:** Hilary Izuchukwu Okagbue

5 Oct 2026

Comparative Evaluation of Machine Learning Algorithms for Maize Lethal Necrosis Disease Detection Under Class Imbalance

PONE-D-26-21835R2

Dear Dr. Wanyonyi,

We’re pleased to inform you that your manuscript has been judged scientifically suitable for publication and will be formally accepted for publication once it meets all outstanding technical requirements.

Within one week, you’ll receive an e-mail detailing the required amendments. When these have been addressed, you’ll receive a formal acceptance letter and your manuscript will be scheduled for publication.

An invoice will be generated when your article is formally accepted. Please note, if your institution has a publishing partnership with PLOS and your article meets the relevant criteria, all or part of your publication costs will be covered. Please make sure your user information is up-to-date by logging into Editorial Manager at Editorial Manager® and clicking the ‘Update My Information' link at the top of the page. For questions related to billing, please contact billing support.

If your institution or institutions have a press office, please notify them about your upcoming paper to help maximize its impact. If they’ll be preparing press materials, please inform our press team as soon as possible -- no later than 48 hours after receiving the formal acceptance. Your manuscript will remain under strict press embargo until 2 pm Eastern Time on the date of publication. For more information, please contact onepress@plos.org.

Kind regards,

Hilary Izuchukwu Okagbue, Ph.D

Academic Editor

PLOS One

Additional Editor Comments (optional):

Reviewers' comments:

Reviewer's Responses to Questions

**Comments to the Author**

Reviewer #1: All comments have been addressed

Reviewer #3: All comments have been addressed

**********

2. Is the manuscript technically sound, and do the data support the conclusions?

Reviewer #1: Yes

Reviewer #3: Yes

**********

3. Has the statistical analysis been performed appropriately and rigorously?

Reviewer #1: Yes

Reviewer #3: Yes

**********

4. Have the authors made all data underlying the findings in their manuscript fully available?

The PLOS Data policy

Reviewer #1: Yes

Reviewer #3: Yes

**********

5. Is the manuscript presented in an intelligible fashion and written in standard English?

Reviewer #1: Yes

Reviewer #3: Yes

**********

Reviewer #1: The authors have fully addressed all concerns raised across two previous rounds of review. The two mandatory corrections identified in Round 2 have both been resolved: the erroneous SMOTE-ENN reference in the results section has been corrected to reflect the technique actually used, and the Data Availability Statement now lists both the dataset DOI and the separate code repository DOI explicitly. The moderate concern regarding feature extraction transparency has also been addressed with an appropriate addition to the Limitations section. The manuscript is now suitable for publication.

Reviewer #3: The authors have considerably improved the manuscript. Therefore, I recommend the acceptance of this manuscript.

**********

what does this mean?). If published, this will include your full peer review and any attached files.

If you choose “no”, your identity will remain anonymous but your review may still be made public.

**Do you want your identity to be public for this peer review?**  For information about this choice, including consent withdrawal, please see our Privacy Policy

Reviewer #1: No

Reviewer #3: No

**********

# Editor report: Acceptance letter

**DOI:** 10.1371/journal.pone.0349738.r006

**Authors:** Hilary Izuchukwu Okagbue

PONE-D-26-21835R2

PLOS One

Dear Dr. Wanyonyi,

I'm pleased to inform you that your manuscript has been deemed suitable for publication in PLOS One. Congratulations! Your manuscript is now being handed over to our production team.

At this stage, our production department will prepare your paper for publication. This includes ensuring the following:

* All references, tables, and figures are properly cited

* All relevant supporting information is included in the manuscript submission,

* There are no issues that prevent the paper from being properly typeset

You will receive further instructions from the production team, including instructions on how to review your proof when it is ready. Please keep in mind that we are working through a large volume of accepted articles, so please give us a few days to review your paper and let you know the next and final steps.

Lastly, if your institution or institutions have a press office, please let them know about your upcoming paper now to help maximize its impact. If they'll be preparing press materials, please inform our press team within the next 48 hours. Your manuscript will remain under strict press embargo until 2 pm Eastern Time on the date of publication. For more information, please contact onepress@plos.org.

You will receive an invoice from PLOS for your publication fee after your manuscript has reached the completed accept phase. If you receive an email requesting payment before acceptance or for any other service, this may be a phishing scheme. Learn how to identify phishing emails and protect your accounts at https://explore.plos.org/phishing.

If we can help with anything else, please email us at customercare@plos.org.

Thank you for submitting your work to PLOS One and supporting open access.

Kind regards,

PLOS One Editorial Office Staff

on behalf of

Dr Hilary Izuchukwu Okagbue

Academic Editor

PLOS One
